# The NORMAN Suspect List Exchange (NORMAN-SLE): facilitating European and worldwide collaboration on suspect screening in high resolution mass spectrometry

**DOI:** 10.1186/s12302-022-00680-6

**Published:** 2022-10-21

**Authors:** Hiba Mohammed Taha, Reza Aalizadeh, Nikiforos Alygizakis, Jean-Philippe Antignac, Hans Peter H. Arp, Richard Bade, Nancy Baker, Lidia Belova, Lubertus Bijlsma, Evan E. Bolton, Werner Brack, Alberto Celma, Wen-Ling Chen, Tiejun Cheng, Parviel Chirsir, Ľuboš Čirka, Lisa A. D’Agostino, Yannick Djoumbou Feunang, Valeria Dulio, Stellan Fischer, Pablo Gago-Ferrero, Aikaterini Galani, Birgit Geueke, Natalia Głowacka, Juliane Glüge, Ksenia Groh, Sylvia Grosse, Peter Haglund, Pertti J. Hakkinen, Sarah E. Hale, Felix Hernandez, Elisabeth M.-L. Janssen, Tim Jonkers, Karin Kiefer, Michal Kirchner, Jan Koschorreck, Martin Krauss, Jessy Krier, Marja H. Lamoree, Marion Letzel, Thomas Letzel, Qingliang Li, James Little, Yanna Liu, David M. Lunderberg, Jonathan W. Martin, Andrew D. McEachran, John A. McLean, Christiane Meier, Jeroen Meijer, Frank Menger, Carla Merino, Jane Muncke, Matthias Muschket, Michael Neumann, Vanessa Neveu, Kelsey Ng, Herbert Oberacher, Jake O’Brien, Peter Oswald, Martina Oswaldova, Jaqueline A. Picache, Cristina Postigo, Noelia Ramirez, Thorsten Reemtsma, Justin Renaud, Pawel Rostkowski, Heinz Rüdel, Reza M. Salek, Saer Samanipour, Martin Scheringer, Ivo Schliebner, Wolfgang Schulz, Tobias Schulze, Manfred Sengl, Benjamin A. Shoemaker, Kerry Sims, Heinz Singer, Randolph R. Singh, Mark Sumarah, Paul A. Thiessen, Kevin V. Thomas, Sonia Torres, Xenia Trier, Annemarie P. van Wezel, Roel C. H. Vermeulen, Jelle J. Vlaanderen, Peter C. von der Ohe, Zhanyun Wang, Antony J. Williams, Egon L. Willighagen, David S. Wishart, Jian Zhang, Nikolaos S. Thomaidis, Juliane Hollender, Jaroslav Slobodnik, Emma L. Schymanski

**Affiliations:** 1 Luxembourg Centre for Systems Biomedicine (LCSB), University of Luxembourg, 6 Avenue du Swing, 4367 Belvaux, Luxembourg; 2 Laboratory of Analytical Chemistry, Department of Chemistry, National and Kapodistrian University of Athens, Panepistimiopolis Zografou, 15771 Athens, Greece; 3 Environmental Institute, Okružná 784/42, 972 41 Koš, Slovak Republic; 4 Oniris, INRAE, LABERCA, 44307 Nantes, France; 5 Norwegian Geotechnical Institute (NGI), Ullevål Stadion, P.O. Box 3930, 0806 Oslo, Norway; 6 Department of Chemistry, Norwegian University of Science and Technology (NTNU), 7491 Trondheim, Norway; 7 Queensland Alliance for Environmental Health Sciences (QAEHS), The University of Queensland, Woolloongabba, QLD 4102 Australia; 8 Leidos, Research Triangle Park, NC USA; 9 Toxicological Centre, University of Antwerp, Antwerp, Belgium; 10 Environmental and Public Health Analytical Chemistry, Research Institute for Pesticides and Water, University Jaume I, Castelló, Spain; 11 National Center for Biotechnology Information, National Library of Medicine, National Institutes of Health, 8600 Rockville Pike, Bethesda, MD 20894 USA; 12 UFZ, Helmholtz Centre for Environmental Research, Leipzig, Germany; 13 Institute of Ecology, Evolution and Diversity, Goethe University, Frankfurt Am Main, Germany; 14 Swedish University of Agricultural Sciences (SLU), Uppsala, Sweden; 15 Institute of Food Safety and Health, College of Public Health, National Taiwan University, 17 Xuzhou Rd., Zhongzheng Dist., Taipei, Taiwan; 16 Faculty of Chemical and Food Technology, Institute of Information Engineering, Automation, and Mathematics, Slovak University of Technology in Bratislava (STU), Radlinského 9, 812 37 Bratislava, Slovak Republic; 17 Science for Life Laboratory, Department of Environmental Science, Stockholm University, 10691 Stockholm, Sweden; 18 Corteva Agriscience, Indianapolis, IN USA; 19 INERIS, National Institute for Environment and Industrial Risks, Verneuil en Halatte, France; 20 Swedish Chemicals Agency (KEMI), P.O. Box 2, 172 13 Sundbyberg, Sweden; 21 Institute of Environmental Assessment and Water Research-Severo Ochoa Excellence Center (IDAEA), Spanish Council of Scientific Research (CSIC), Barcelona, Spain; 22 Food Packaging Forum Foundation, Staffelstrasse 10, 8045 Zurich, Switzerland; 23 Institute of Biogeochemistry and Pollutant Dynamics, ETH Zurich, 8092 Zurich, Switzerland; 24 Eawag, Swiss Federal Institute for Aquatic Science and Technology, Überlandstrasse 133, 8600 Dübendorf, Switzerland; 25 Thermo Fisher Scientific, Dornierstrasse 4, 82110 Germering, Germany; 26 Department of Chemistry, Chemical Biological Centre (KBC), Umeå University, Linnaeus Väg 6, 901 87 Umeå, Sweden; 27 Department Environment and Health, Amsterdam Institute for Life and Environment, Vrije Universiteit, Amsterdam, The Netherlands; 28 Water Research Institute (WRI), Nábr. Arm. Gen. L. Svobodu 5, 81249 Bratislava, Slovak Republic; 29 German Environment Agency (UBA), Wörlitzer Platz 1, Dessau-Roßlau, Germany; 30 Bavarian Environment Agency, 86179 Augsburg, Germany; 31 Analytisches Forschungsinstitut Für Non-Target Screening GmbH (AFIN-TS), Am Mittleren Moos 48, 86167 Augsburg, Germany; 32 Mass Spec Interpretation Services, 3612 Hemlock Park Drive, Kingsport, TN 37663 USA; 33 State Key Laboratory of Environmental Chemistry and Ecotoxicology, Research Center for Eco-Environmental Sciences, Chinese Academy of Sciences (SKLECE, RCEES, CAS), No. 18 Shuangqing Road, Haidian District, Beijing, 100086 China; 34 Hope College, Holland, MI 49422 USA; 35 University of California, Berkeley, CA USA; 36 Agilent Technologies, Inc., 5301 Stevens Creek Blvd, Santa Clara, CA 95051 USA; 37 Department of Chemistry, Center for Innovative Technology, Vanderbilt-Ingram Cancer Center, Vanderbilt Institute of Chemical Biology, Vanderbilt Institute for Integrative Biosystems Research and Education, Vanderbilt University, Nashville, TN 37235 USA; 38 Institute for Risk Assessment Sciences (IRAS), Utrecht University, Utrecht, The Netherlands; 39 University Rovira i Virgili, Tarragona, Spain; 40 Biosfer Teslab, Reus, Spain; 41 Nutrition and Metabolism Branch, International Agency for Research On Cancer (IARC), 150 Cours Albert Thomas, 69372 Lyon Cedex 08, France; 42 RECETOX, Faculty of Science, Masaryk University, Kotlářská 2, Brno, Czech Republic; 43 Institute of Legal Medicine and Core Facility Metabolomics, Medical University of Innsbruck, Muellerstrasse 44, Innsbruck, Austria; 44 Technologies for Water Management and Treatment Research Group, Department of Civil Engineering, University of Granada, Campus de Fuentenueva S/N, 18071 Granada, Spain; 45 Institute of Health Research Pere Virgili, Tarragona, Spain; 46 Agriculture and Agri-Food Canada/Agriculture et Agroalimentaire Canada, 1391 Sandford Street, London, ON N5V 4T3 Canada; 47 NILU, Norwegian Institute for Air Research, Kjeller, Norway; 48 Fraunhofer Institute for Molecular Biology and Applied Ecology (Fraunhofer IME), Schmallenberg, Germany; 49 Van’t Hoff Institute for Molecular Sciences, University of Amsterdam, P.O. Box 94157, Amsterdam, 1090 GD The Netherlands; 50 Laboratory for Operation Control and Research, Zweckverband Landeswasserversorgung, Am Spitzigen Berg 1, 89129 Langenau, Germany; 51 Environment Agency, Horizon House, Deanery Road, Bristol, BS1 5AH UK; 52 Chemical Contamination of Marine Ecosystems (CCEM) Unit, Institut Français de Recherche pour l’Exploitation de la Mer (IFREMER), Rue de l’Ile d’Yeu, BP 21105, 44311 Cedex 3, Nantes France; 53 Section for Environmental Chemistry and Physics, Plant and Environmental Sciences, University of Copenhagen, Thorvaldsensvej 40, 1871 Frederiksberg C, Denmark; 54 Institute for Biodiversity and Ecosystem Dynamics, University of Amsterdam, Amsterdam, The Netherlands; 55 Technology and Society Laboratory, Empa-Swiss Federal Laboratories for Materials Science and Technology, Lerchenfeldstrasse 5, 9014 St. Gallen, Switzerland; 56 Computational Chemistry and Cheminformatics Branch (CCCB), Chemical Characterization and Exposure Division (CCED), Center for Computational Toxicology and Exposure (CCTE), United States Environmental Protection Agency, 109 T.W. Alexander Drive, Research Triangle Park, NC 27711 USA; 57 Department of Bioinformatics-BiGCaT, NUTRIM, Maastricht University, Maastricht, The Netherlands; 58 University of Alberta, Edmonton, AB T6G 2G3 Canada

**Keywords:** Suspect screening, High resolution mass spectrometry, Non-target screening, Open science, FAIR (Findable Accessible Interoperable Reusable) data, Data exchange, Cheminformatics, Exposomics, Environmental contaminants, Chemicals of emerging concern

## Abstract

**Background:**

The NORMAN Association (https://www.norman-network.com/) initiated the NORMAN Suspect List Exchange (NORMAN-SLE; https://www.norman-network.com/nds/SLE/) in 2015, following the NORMAN collaborative trial on non-target screening of environmental water samples by mass spectrometry. Since then, this exchange of information on chemicals that are expected to occur in the environment, along with the accompanying expert knowledge and references, has become a valuable knowledge base for “suspect screening” lists. The NORMAN-SLE now serves as a FAIR (Findable, Accessible, Interoperable, Reusable) chemical information resource worldwide.

**Results:**

The NORMAN-SLE contains 99 separate suspect list collections (as of May 2022) from over 70 contributors around the world, totalling over 100,000 unique substances. The substance classes include per- and polyfluoroalkyl substances (PFAS), pharmaceuticals, pesticides, natural toxins, high production volume substances covered under the European REACH regulation (EC: 1272/2008), priority contaminants of emerging concern (CECs) and regulatory lists from NORMAN partners. Several lists focus on transformation products (TPs) and complex features detected in the environment with various levels of provenance and structural information. Each list is available for separate download. The merged, curated collection is also available as the NORMAN Substance Database (NORMAN SusDat). Both the NORMAN-SLE and NORMAN SusDat are integrated within the NORMAN Database System (NDS). The individual NORMAN-SLE lists receive digital object identifiers (DOIs) and traceable versioning via a Zenodo community (https://zenodo.org/communities/norman-sle), with a total of > 40,000 unique views, > 50,000 unique downloads and 40 citations (May 2022). NORMAN-SLE content is progressively integrated into large open chemical databases such as PubChem (https://pubchem.ncbi.nlm.nih.gov/) and the US EPA’s CompTox Chemicals Dashboard (https://comptox.epa.gov/dashboard/), enabling further access to these lists, along with the additional functionality and calculated properties these resources offer. PubChem has also integrated significant annotation content from the NORMAN-SLE, including a classification browser (https://pubchem.ncbi.nlm.nih.gov/classification/#hid=101).

**Conclusions:**

The NORMAN-SLE offers a specialized service for hosting suspect screening lists of relevance for the environmental community in an open, FAIR manner that allows integration with other major chemical resources. These efforts foster the exchange of information between scientists and regulators, supporting the paradigm shift to the “one substance, one assessment” approach. New submissions are welcome via the contacts provided on the NORMAN-SLE website (https://www.norman-network.com/nds/SLE/).

**Supplementary Information:**

The online version contains supplementary material available at 10.1186/s12302-022-00680-6.

## Background

In environmental analytical chemistry, suspect screening typically involves the use of high resolution mass spectrometry (HRMS) to search for the presence of chemicals in environmental samples based on suspect lists, using the exact mass as a first step in the annotation of detected features [1, 2]. Suspect screening has grown in popularity over the last few years as an efficient way to complement traditional target analysis approaches, where a reference standard is required, without performing a time-intensive non-target screening of the tens of thousands of unknown features typical in environmental samples using extensive compound databases. Several publications describe these approaches in greater detail [1–4]. The NORMAN Association (a network of reference laboratories for monitoring of contaminants of emerging concern (CECs) in the environment—hereafter “NORMAN”) [5] ran the first non-target screening (NTS) collaborative trial on river water in 2013/2014 [4]. The results showed that participants tentatively identified roughly as many chemicals via both suspect and target screening methods, but very few via NTS [4]. This early effort demonstrated that suspect screening approaches were more efficient and popular across the 19 participating institutes, offering a much higher annotation rate than non-target identification. Since then, NORMAN has run further collaborative trials involving suspect screening, including dust [6], passive samplers [7] and biota [8]. Suspect screening has also gained popularity beyond environmental studies and matrices, expanding recently to biomonitoring (e.g., [9, 10]).

One major outcome of the 2013/2014 NORMAN NTS collaborative trial was the clear need for a better exchange of chemical information both among and beyond NORMAN members [4], since the 2013/2014 collaborative trial participants used an incredibly wide variety of data sources during the trial (shown in Table 3 of [4]). This need had already been identified earlier, for example in the MODELKEY project [11] that included several NORMAN members, but the right implementation strategy remained elusive. A second NTS collaborative trial outcome, discussed in subsequent workshops, was a debate between “screen smart”, versus “screen big”. At the time, the “screen smart” strategy had been employed, for example, to study pesticides [12], pharmaceuticals [13] and surfactants [14] using relatively small lists (185, 980 and 394 entries, respectively), to support focussed research questions. In contrast, the “screen big” strategy used very large lists containing thousands of chemicals (e.g., lists of high production volume chemicals registered under the European Registration, Evaluation, Authorisation and Restriction of Chemicals (REACH) regulation (EC No 1272/2008)) to find more hits—with the accompanying risk of many more false positives (see e.g., [15, 16]). Naturally, the boundary between these two strategies blurred over time, as some “smart” suspect lists also became quite “big”. For instance, the STOFF-IDENT (https://water.for-ident.org/#!home) compilation of water-relevant contaminants such as pesticides, pharmaceuticals and industrial chemicals [17] includes over 10,500 substances. This list is “smart” with respect to the relevance to the water compartment, but with many pollutant classes and a large proportion of REACH chemicals, the overall number of chemicals is large enough to increase the probability of generating many false-positive results. In the extreme, “screen big” could be extended to candidates from even larger compound databases with millions of entries, which are commonly used in NTS approaches—with the lower success rates (i.e., more false positives) as mentioned above. Since suspect screening approaches typically start with only an exact mass of the expected adduct(s) of the suspects, there is a large burden of proof to confirm that the “suspect hit” is actually present, as discussed elsewhere [2–4].

The exchange of and access to chemical information in an open (i.e., free to access, publicly available) manner [18] has not always been as easy as it appears today. A key breakthrough was achieved in 2004 with the launch of PubChem (https://pubchem.ncbi.nlm.nih.gov/) [19], currently one of the largest open chemical knowledge bases with extensive information on over 111 million chemicals (July 2022). The ChemSpider collection was released a few years later (http://www.chemspider.com/) [20] and now contains 114 million chemicals (July 2022). The United States Environmental Protection Agency (US EPA) released the CompTox Chemicals Dashboard (https://comptox.epa.gov/dashboard/) [21] (hereafter “CompTox”) in 2016 as a smaller collection, currently of 906,511 chemicals (July 2022) related to environmental and toxicology questions. Likewise, in 2016 the term “*FAIR*” was coined, describing how to make research more *F*indable, *A*ccessible, *I*nteroperable and *R*eusable [22, 23]. Together, ensuring that data is both Open and FAIR is a powerful combination [24]. The European Union (EU) is also embracing Open and FAIR principles. The European Chemicals Agency (ECHA) [25] and the European Food and Safety Authority (EFSA) [26] are transitioning their information to be more Open and FAIR, while Joint Research Centre (JRC) has released the Information Portal for Chemical Monitoring (IPCHEM) for the exchange of monitoring data in Europe [27]. Recent initiatives such as the European Partnership for Chemicals Risk Assessment (PARC) [28, 29] and the Environmental Exposure Assessment Research Infrastructure (EIRENE) [30] will strengthen this into the future.

In response to the NORMAN NTS collaborative trial outcomes, NORMAN initiated the NORMAN Suspect List Exchange (NORMAN-SLE, https://www.norman-network.com/nds/SLE/) in 2015 as part of the NORMAN Database System (NDS, https://www.norman-network.com/nds/) [29, 31] to facilitate the open access exchange of various suspect lists within and beyond Europe. This FAIR, open access, whole community initiative is not limited to NORMAN members. The primary aim of the NORMAN-SLE is to provide a location where suspect lists are publicly accessible, together with appropriate reference information, for interested parties to browse and select as desired (facilitating the “screen smart” approach). The NORMAN-SLE forms the basis for the NORMAN Substance Database (NORMAN SusDat, https://www.norman-network.com/nds/susdat/), a merged and curated data table with additional parameters for use in NORMAN activities (to facilitate the “screen big” approach), which will be described in more detail in a separate article. The present article covers the creation and implementation of the NORMAN-SLE as an Open and FAIR data resource, along with its integration with major open chemistry resources (PubChem, CompTox) as described below in the methods section, followed by an overview of the current state, implications and outlook in the results and discussion sections.

## Methods

### NORMAN Suspect List Exchange (NORMAN-SLE) website

**Fig. 1:**
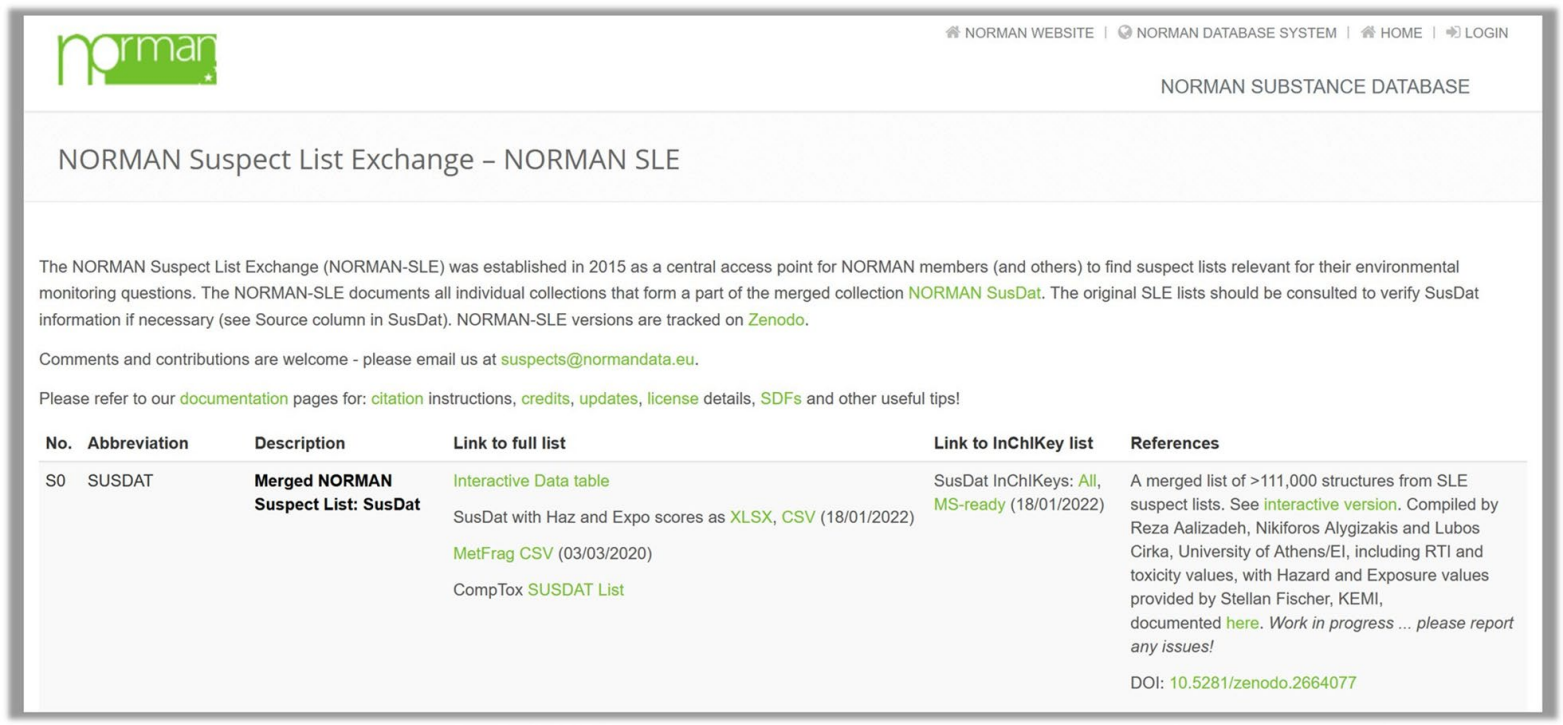
Screenshot of the NORMAN Suspect List Exchange (https://www.norman-network.com/nds/SLE/) [32]

The principle behind the NORMAN-SLE is simple: facilitating the exchange of chemical information to support the suspect screening of primarily organic contaminants amenable to liquid or gas chromatography (LC or GC) coupled to mass spectrometry. The website itself (https://www.norman-network.com/nds/SLE/) contains a simple overview of the background behind the NORMAN-SLE and a table containing the suspect lists themselves (with the fields “Number”, “Abbreviation”, “Description”, “Link to full list”, “Link to InChIKey list” and “References”), as shown in Fig. 1 and explained further below. Each list has a number (starting with S0 for SUSDAT, the merged collection), increasing sequentially with every contribution, along with an abbreviation for easier integration, access, and recognition.

The idea behind the simplicity of this website is to enable public access to various suspect lists as close as possible to the lists used in original publications, but with a reasonable degree of standardization and, where possible, added value to enhance and FAIRify these lists for future use (see below). If major adjustments were made to a submitted list, the original list is provided along with modified versions, so that both sets of information are available.

### Information content and preparation of suspect lists hosted on the NORMAN-SLE

The minimum information available in most lists is a name and at least one additional identifier, although in most lists, far more information is available. At least one chemical name (plus other synonyms if available) should be included. The preferred formats for structural information are the simplified molecular-input line-entry system (SMILES) [33] plus the International Chemical Identifier (InChI) in the form of standard InChI and InChIKey [34]. Common database identifiers provided typically include one (or more) of either Chemical Abstract Service (CAS) number(s) [35], EC number [36], PubChem Compound Identifier (CID) [19], ChemSpider identifier (CSID) [20] and/or the Distributed Structure-Searchable Toxicity (DSSTox) substance identifier (DTXSID) used in CompTox [21]. To support suspect screening, the (neutral) monoisotopic masses and molecular formulae are included in many of the lists. This information, along with several other predicted values, is also included in the merged NORMAN SusDat. Several other fields may be present, depending on the context of the suspect list, and are included where available. More details on the chemical structure identifiers and recommended chemical structural data templates are provided elsewhere [24, 37].

**Fig. 2:**
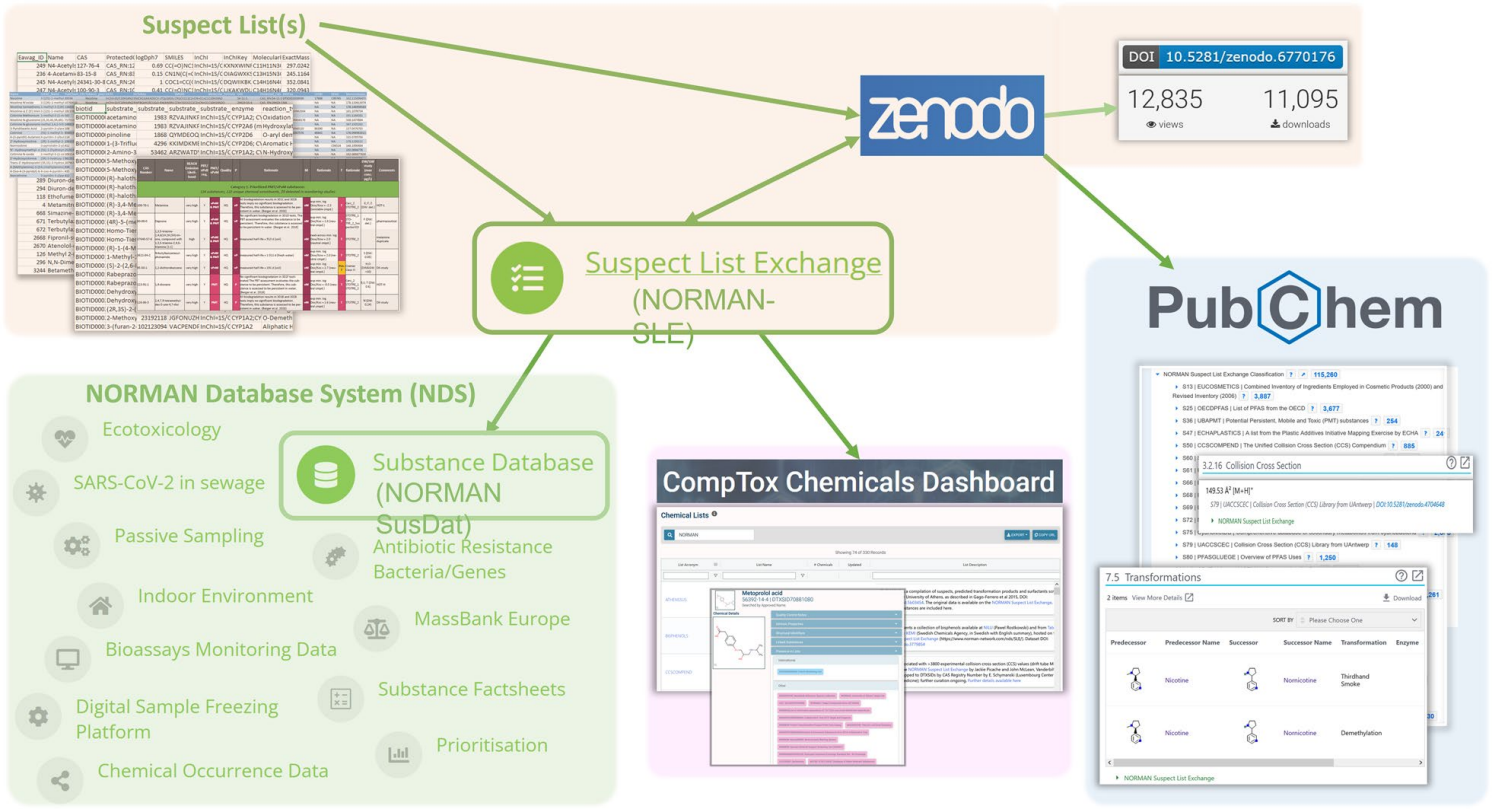
Schematic showing the relationships between submitted suspect lists, the NORMAN-SLE and downstream resources. Top (orange shading): suspect lists submitted in various formats are curated, then added to the NORMAN-SLE website (centre) and archived on the NORMAN-SLE Zenodo community (top right), yielding a DOI and use statistics. Bottom left (green shading): the NORMAN-SLE serves as an information source for NORMAN SusDat and the NORMAN Database System (NDS). Bottom middle (pink shading): NORMAN-SLE lists are integrated in CompTox manually. Bottom right (blue shading): NORMAN-SLE content is harvested from Zenodo via mapping files and integrated into PubChem in an automated workflow

The suspect lists (commonly submitted via email to NORMAN contact points, see Fig. 2, top left) are processed upon submission, with the subsequent processing steps highly dependent on both the type of submission and the size of the list. While the suspect list number is assigned sequentially, the abbreviation, name and description are assigned following pre-defined conventions, and in discussion with authors. Where necessary, curation is performed on these lists to fill in missing values where at least a chemical identifier and/or structural information and/or (correct) name was provided. For some lists, the missing values are filled using automated workflows covering a variety of web services (depending on the list and contributor) from PubChem [19], ChemSpider [20] and CACTUS (https://cactus.nci.nih.gov/), typically via RMassBank [38], RChemMass [39] and other related packages in the R programming language. Other lists are processed with batch services offered through PubChem [19, 40] and CompTox [21, 41]. Additional chemical structure interconversions (e.g., SMILES to InChI) are performed with OpenBabel (http://openbabel.org/) [42] or the Chemistry Development Kit (CDK) (usually via R) [43] where necessary. Note that the curation performed on the individual suspect lists is independent of the curation and merging to form the NORMAN SusDat collection (see Fig. 2, bottom left), which will be detailed in a separate publication. The processes evolve over time as new technical possibilities arise (e.g., batch searching). The resulting suspect lists are generally provided as Excel (XLSX) and comma separated values (CSV) formats, as standardized as reasonably possible, on the website. The CSV format provides greater interoperability, including allowing import into various libraries, vendor and open software, as well as PubChem (described below). A separate InChIKey file is also provided, as this allows fast screening of suspects within the in silico fragmenter MetFrag [44] and other approaches. For some of the lists, additional files are provided, to disseminate all the relevant details. Finally, references and additional information are given, to acknowledge contributors, but also to provide users quick access to the rationale behind each individual suspect list. Further details on the NORMAN-SLE contents, including references, are given in the Results section.

Several suspect lists contain partial, incomplete, or even no structural information, such as the per- and polyfluoroalkyl substances (PFAS) lists S9 PFASTRIER [45] (e.g., elemental compositions retrieved from patents where no structural or isomer information was available) and S46 PFASNTREV19 [46, 47] (a compilation of PFAS identification efforts in non-target screening studies), as well as the surfactant isomer list S18 TSCASURF [48]. Nevertheless, these lists still provide vital information for identification by mass and/or molecular formula (see e.g., [14, 49], where whole surfactant classes can be identified via the general formula of a homologous series of several structural isomers). For those lists with partial information, missing values were filled in, where possible, as described above, and were saved in separate files or as multiple sheets in one file. Associated InChIKey lists were only generated for known structures. Dealing with partially characterized molecular features or chemical substances of Unknown or Variable Composition, Complex Reaction Products or Biological Materials (UVCB substances, UVCBs) is a subject of future collaborations beyond the scope of the current article (see e.g., [50, 51]), as discussed further below.

### NORMAN-SLE on Zenodo

**Fig. 3:**
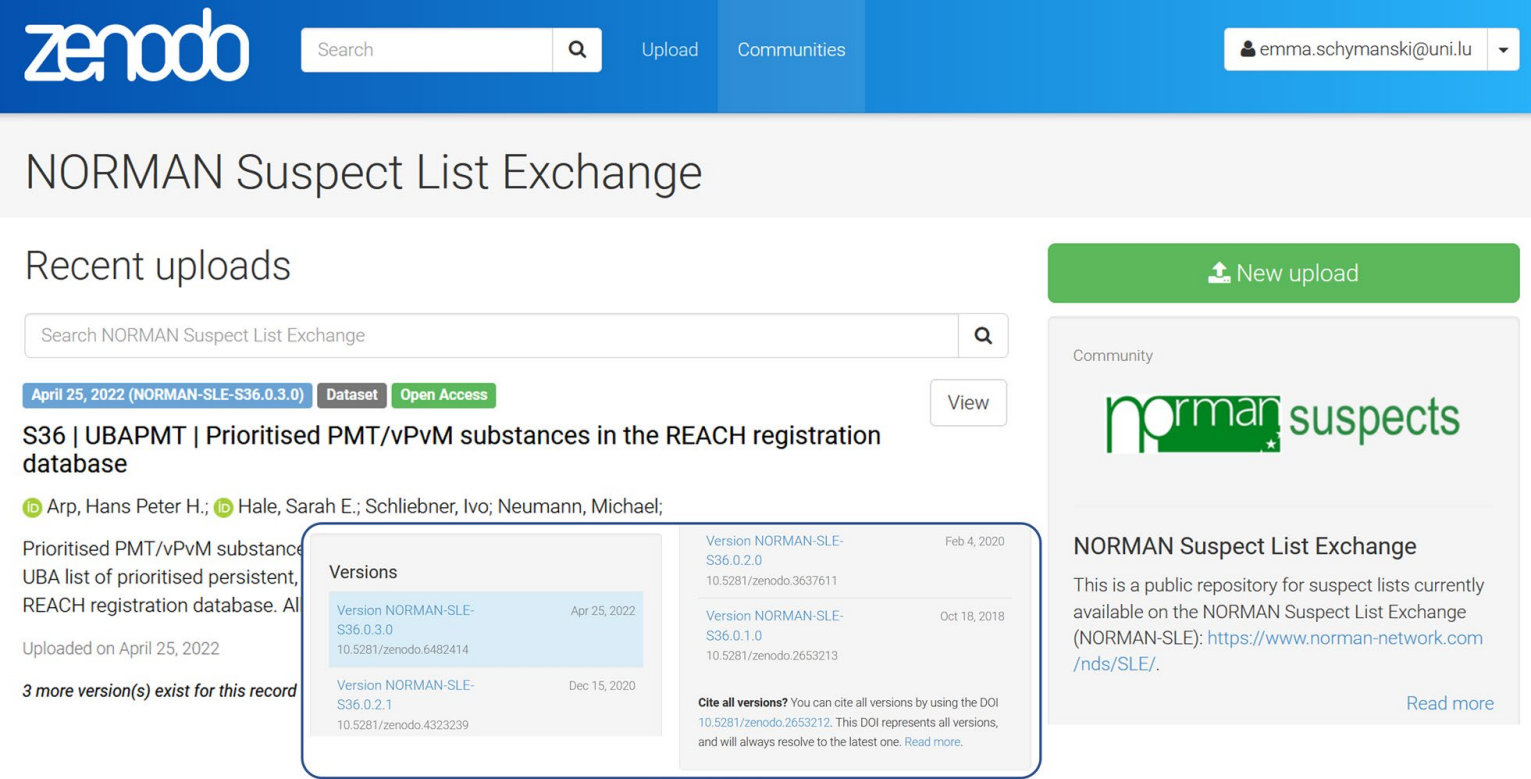
The NORMAN Suspect List Exchange Zenodo community (https://zenodo.org/communities/norman-sle) with inset showing the versioning history of S36 UBAPMT (https://doi.org/10.5281/zenodo.2653212) [53, 54]

The development of the Zenodo repository [52] enabled public archiving, versioning and generation of a Digital Object Identifier (DOI) for each NORMAN-SLE list. Thus, since 2019, the NORMAN-SLE content has been uploaded to and archived on the Zenodo repository [52], gathered under the NORMAN-SLE community (https://zenodo.org/communities/norman-sle/) [53]. Each individual NORMAN-SLE collection has its own Zenodo record and thus a dataset DOI, allowing users to cite the individual lists directly, including specific versions, or all versions. Updates to lists can thus be tracked under the Zenodo version control system, with the master DOI always redirecting to the latest version. The lists are tracked under a versioning system following the pattern NORMAN-SLE-SXX-0.Y.Z, where SXX refers to the list number (as on the NORMAN-SLE website and as described below) and the 0.Y.Z pattern records whether it was a major update (Y is increased incrementally by 1) or minor update (Z is increased incrementally by 1). The leading “0” is currently a buffer. Major updates constitute new entries (e.g., new chemicals, rows, information, updates) to the lists, while minor updates are corrections or adjustments to the current contents without adding major new content (e.g*.*, correcting names, identifiers, typographical errors). The presence on Zenodo has enabled better citation, the tracking of use statistics at an individual list level and additional possibilities for the integration with external resources such as PubChem, as shown in Fig. 2 (right) and discussed further below. Figure 3 shows the presence of the NORMAN-SLE on Zenodo, including versioning in the inset.

### NORMAN-SLE and CompTox Chemicals Dashboard ﻿integration

Since CompTox [21] is a highly relevant resource for environmental and toxicological information, integration of NORMAN-SLE content is of interest to both parties and is achieved via the “Chemical Lists” functionality (https://comptox.epa.gov/dashboard/chemical-lists/). The integration started in 2017 and is performed through the upload of the DTXSIDs associated with the individual NORMAN-SLE lists to the DSSTox database [55] that underlies CompTox. Most lists have the NORMAN keyword associated with it, such that they are accessible through the URL https://comptox.epa.gov/dashboard/chemical-lists?search=NORMAN, or through a direct URL composed of the list code (e.g*.*, https://comptox.epa.gov/dashboard/chemical-lists/BISPHENOLS for the S20 BISPHENOLS list). Several lists on the NORMAN-SLE were produced in a collaborative curation effort (e.g*.,* S24 HUMANNEUROTOX [56], S37 LITMINEDNEURO [57] and S43 NEUROTOXINS [58], as part of [59]), or were curated and registered by the DSSTox curation team before uploading to the SLE (e.g*.,* S25 OECDPFAS [60–62]). Some other lists on the NORMAN-SLE were sourced directly from CompTox as they contained entries highly relevant for the NORMAN Database System (e.g*.,* S45 SYNTHCANNAB [63] and S58 PSYCHOCANNAB [64]). For recent lists, generally the CompTox batch search (https://comptox.epa.gov/dashboard/batch-search) [65] is used to retrieve DTXSIDs on the basis of the user-provided information, which are then provided directly to CompTox along with the list code, name and description for upload. The presence of compounds in NORMAN-SLE lists appear on the individual chemical records in CompTox (see pink entries in the inset in Fig. 2) and can also be identified by prefiltering in the CompTox batch search interface and including flags in the export files.

Due to the infrequent release of updates to CompTox, it may be many weeks or months before new NORMAN-SLE lists are available publicly on CompTox. Currently, 88 of the 99 NORMAN-SLE lists are on CompTox (see Additional file 1), with 74 listed under the “NORMAN” URL above. Since not all substances in the NORMAN-SLE are currently present in CompTox, the mapping of NORMAN-SLE lists in CompTox is often incomplete, i.e., the lists on CompTox contain only entries for which DTXSIDs currently exist (further details are provided in Additional file 1).

### NORMAN-SLE and PubChem integration

As one of the largest open chemical databases with millions of monthly users, integration of NORMAN-SLE content in PubChem has great potential to increase the visibility of this community effort. The NORMAN-SLE integration with PubChem [19] (https://pubchem.ncbi.nlm.nih.gov/) commenced in 2019. The first substance deposition was processed on November 22, 2019. The deposition file is compiled from all lists by the PubChem team, via a mapping file hosted on the Environmental Cheminformatics (ECI) group (University of Luxembourg) GitLab pages [66]. This mapping file contains a link to the latest version of each suspect list (CSV file) on Zenodo, the list details and version, the dataset DOI, extra DOIs (to include related publications), mappings to the columns containing the chemical identifiers (SMILES, InChIKey, InChI, Synonym), the NORMAN-SLE URL and a comment field. The compiled deposition file is mapped to PubChem Substance Identifiers (SIDs) and PubChem Compound Identifiers (CIDs) via the PubChem deposition system. While SIDs are available for all substances deposited to PubChem (including those with undefined structures), CIDs are only available for all unique chemical structures (i.e., defined chemical structures) extracted from substance depositions via the PubChem standardization process [67]. As a result, the number of compounds (CIDs) will generally be less than the number of substances (SIDs). Any SMILES errors found during deposition are debugged in collaboration with the PubChem team and any dataset-specific causes are fixed in the corresponding NORMAN-SLE datasets by releasing new minor versions on Zenodo (see e.g., descriptions in [68, 69]). Synonyms are currently provided as a small, manually curated file containing the columns CID, InChIKey, Synonym, Reference DOI and Dataset information (114 entries on 30 April 2022, see [70]) to specifically add missing synonyms to PubChem [70]. These are primarily newly deposited structures (i.e., structures not yet in PubChem) associated with S74 REFTPS [71] and S96 ECIPFAS [72]. The PubChem/NORMAN-SLE deposition is re-run once updates are available and takes minutes to run. The updated data are live on the public PubChem website within hours to days (newly added structures can take longer to index fully). The latest deposition and number of live substances (i.e., the number of substances currently available on the public website) can be retrieved from the NORMAN-SLE data source page in PubChem [73].

The contents of individual NORMAN-SLE lists are available interactively in PubChem via the NORMAN Suspect List Exchange Tree (https://pubchem.ncbi.nlm.nih.gov/classification/#hid=101, hereafter “PubChem NORMAN-SLE Tree”) on the PubChem Classification Browser [74]. This is compiled by PubChem from a second mapping file, also hosted on the ECI GitLab pages [75]. For each dataset, this mapping file contains a link to the latest InChIKey file on Zenodo, the list title as it should appear in the tree (e.g*.,* “S00 | SUSDAT | Merged NORMAN Suspect List: SusDat”) and a tool tip, i.e., further details about the list that displays when users click the “?” icon on the Classification Browser (see figure in Results section). The mapping file also contains additional fields defining the content of interest (keywords, annotations) and other information for internal housekeeping. All lists (except S18 TSCASURF, for which no InChIKeys exist) are listed in numerical order in the PubChem NORMAN-SLE Tree. In addition, certain lists with detailed classification content appear again at the top of the browser. These are mapped via structural information in the CSVs (not the InChIKey files) to profit from the detailed additional information available in these lists. The PubChem Classification Browser can also be accessed programmatically (i.e., in an automated manner), with documentation available on PubChem [67] and the ECI GitLab pages [76]. The PubChem NORMAN-SLE Tree also enables users to download individual lists (or even various combinations thereof via advanced queries) in the variety of formats offered by PubChem, including the structure data format (SDF) not currently offered on the NORMAN-SLE website, see documentation available in e.g., [77].

PubChem has also integrated several categories of annotation content, i.e., detailed information about individual chemicals, into the compound records in PubChem. As of 30 April 2022, a total of 17 annotation categories, which equate to headers in the Table of Contents entries in PubChem [78], were integrated. Many relate to the chemical role or use (e.g*.,* the Anatomical Therapeutic Chemical (ATC) Code for pharmaceuticals, Agrochemical Category, Chemical Classes, Use Classifications and Uses) and transformation information (e.g*.,* included in the Transformations, Metabolism/Metabolites, Drug Transformations and Agrochemical Transformations headers). Others relate to chemical information (e.g*.,* molecular formula) and measurement data, such as nuclear magnetic resonance (NMR—^13^C, ^19^F, ^1^H, and ^31^P), tandem MS (MS/MS) data and collision cross section (CCS) data from ion mobility experiments. Finally, taxonomy information (functionality recently added to PubChem [79] for organisms) has been included for some lists. All files necessary for the integration of the annotation content within PubChem are present in the Zenodo repository for the respective list, supported by additional mapping or annotation files either added in Zenodo, or hosted on the ECI GitLab pages in the “annotations” subfolder [80] where necessary. The latest overview and the entire content integrated in PubChem (in JSON, XML and ASNT formats, accessible programmatically or for download) is available from the NORMAN-SLE data source page in PubChem [73].

### Results

### Overview of NORMAN-SLE

**Fig. 4:**
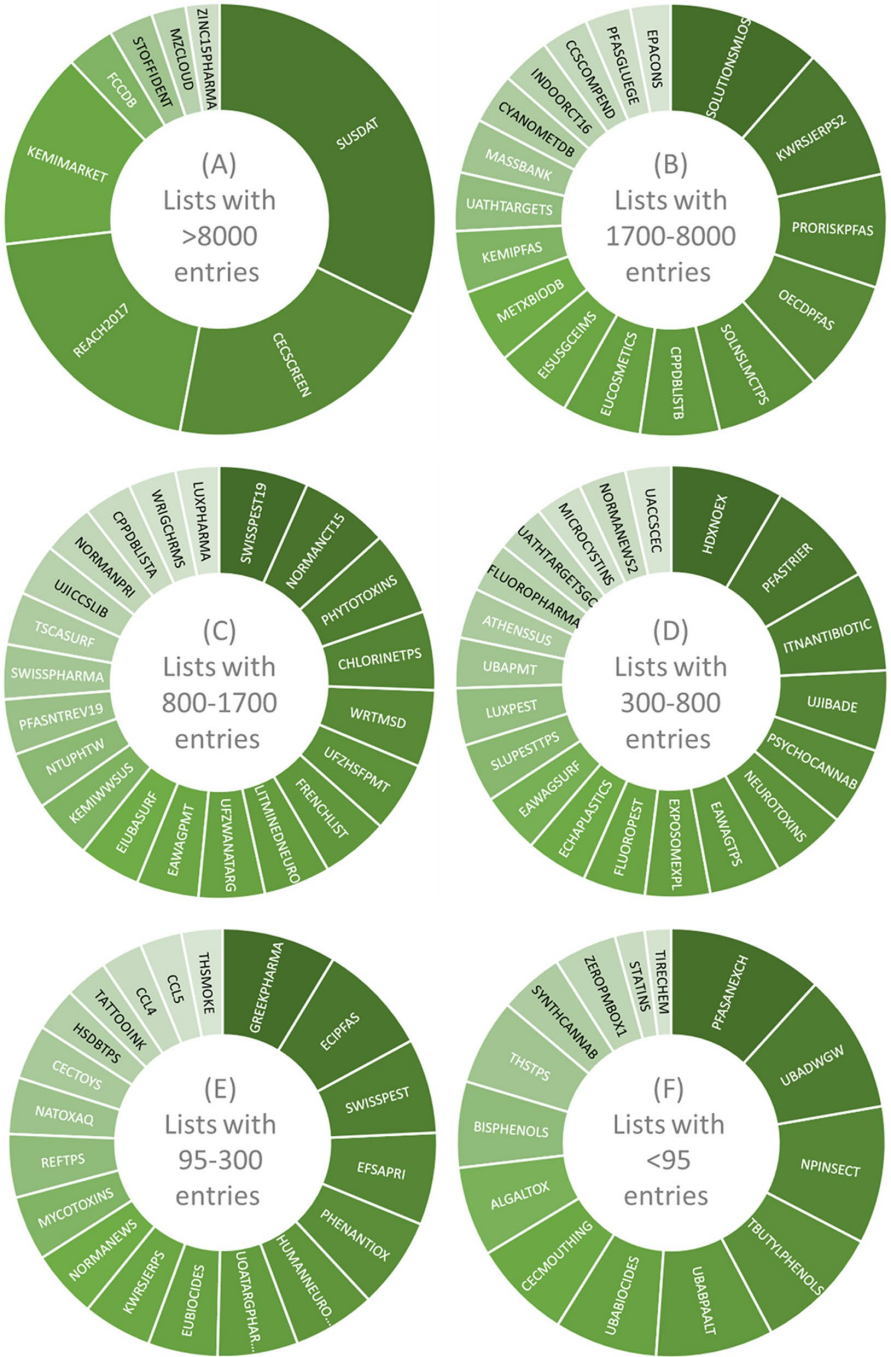
Starburst plots of the 99 suspect lists forming the NORMAN-SLE contents. Lists with: (**A**) > 8000 entries; (**B**) 1700–8000 entries; (**C**) 800–1700 entries; (**D**) 300–800 entries; (**E**) 95–300 entries and (**F**) < 95 entries (ranges chosen to optimize plotting). The list codes, numbers of chemical entries and references are summarized in Table 1 according to the same groups, with full details in Additional file 1

The NORMAN-SLE includes 99 contributions (starting at S0 SUSDAT, the compilation of all NORMAN-SLE lists, to S98 TIRECHEM) from over 70 contributors as of May 2022, summarized in Fig. 4 and Table 1. Full details on all lists are available in Additional file 1 [81], including list details and chemical numbers across the resources in CSV format, and Additional file 2 [82], a May 2022 copy of the NORMAN-SLE website contents.

**Table 1:** Summary of the NORMAN-SLE datasets, split by the groups shown in Fig. 4, with suspect list number (S), code, number of entries (lines in the file, in italics) and the accompanying references

Group	List number, Code, Entries (total lines) and References
(A) > 8000	S0 SUSDAT *109,631* [69, 83]; S71 CECSCREEN *70,397* [84, 85]; S32 REACH2017 *68,679* [86]; S17 KEMIMARKET *50,308* [68]; S77 FCCDB *12,285* [87–89]; S2 STOFFIDENT *11,289* [17, 90]; S19 MZCLOUD *8742* [91]; S55 ZINC15PHARMA *8646* [92–94]
(B) 1700–8000	S33 SOLUTIONSMLOS *6463* [95–97]; S27 KWRSJERPS2 *5702* [98, 99]; S89 PRORISKPFAS *4777* [100, 101]; S25 OECDPFAS *4725* [60–62]; S38 SOLNSLMCTPS *4465* [96, 97, 102]; S49 CPPDBLISTB *3353* [103–105]; S13 EUCOSMETICS *3333* [106–108]; S70 EISUSGCEIMS *3266* [109]; S73 METXBIODB *3148* [110, 111]; S14 KEMIPFAS *2602* [112, 113]; S21 UATHTARGETS *2466* [114, 115]; S1 MASSBANK *2305* [38, 116, 117]; S75 CYANOMETDB *2124* [118, 119]; S35 INDOORCT16 *2056* [6, 120]; S50 CCSCOMPEND *1983* [121–123]; S80 PFASGLUEGE *1926* [124, 125]; S22 EPACONS *1705* [126, 127]
(C) 800–1700	S60 SWISSPEST19 *1664* [128, 129]; S3 NORMANCT15 *1662* [4, 130]; S29 PHYTOTOXINS *1586* [131, 132]; S87 CHLORINETPS *1470* [133, 134]; S31 WRTMSD *1429* [135, 136]; S84 UFZHSFPMT *1310* [137–139]; S16 FRENCHLIST *1256* [140]; S37 LITMINEDNEURO *1243* [57, 59]; S53 UFZWANATARG *1235* [141]; S82 EAWAGPMT *1162* [142, 143]; S23 EIUBASURF *1154* [144]; S39 KEMIWWSUS *1123* [145]; S72 NTUPHTW *1068* [146, 147]; S46 PFASNTREV19 *1030* [46, 47]; S10 SWISSPHARMA *1020* [13, 148]; S18 TSCASURF *985* [48]; S61 UJICCSLIB *970* [149, 150]; S15 NORMANPRI *967* [151]; S48 CPPDBLISTA *902* [103, 105, 152]; S51 WRIGCHRMS *892* [153]; S76 LUXPHARMA *816* [154, 155]
(D) 300–800	S42 HDXNOEX *765* [156, 157]; S9 PFASTRIER *746* [45]; S6 ITNANTIBIOTIC *676* [158, 159]; S4 UJIBADE *544* [160, 161]; S58 PSYCHOCANNAB *531* [64]; S43 NEUROTOXINS *511* [58, 59]; S66 EAWAGTPS *486* [162, 163]; S34 EXPOSOMEXPL *440* [164–166]; S94 FLUOROPEST *423* [167, 168]; S47 ECHAPLASTICS *418* [169, 170]; S7 EAWAGSURF *410* [14, 171]; S78 SLUPESTTPS *400* [172, 173]; S69 LUXPEST *386* [174, 175]; S36 UBAPMT *341* [54, 176, 177]; S8 ATHENSSUS *340* [49, 178]; S92 FLUOROPHARMA *340* [179, 180]; S65 UATHTARGETSGC *334* [181, 182]; S62 NORMANEWS2 *321* [183, 184]; S85 MICROCYSTINS *321* [118, 185]; S79 UACCSCEC *311* [186, 187]
(E) 95–300	S57 GREEKPHARMA *263* [188]; S96 ECIPFAS *258* [72]; S11 SWISSPEST *218* [12, 189]; S54 EFSAPRI *212* [190, 191]; S30 PHENANTIOX *209* [192]; S24 HUMANNEUROTOX *190* [56, 59]; S56 UOATARGPHARMA *185* [193–195]; S28 EUBIOCIDES *160* [196]; S5 KWRSJERPS *159* [99, 197]; S12 NORMANEWS *156* [198, 199]; S26 MYCOTOXINS *149* [200]; S74 REFTPS *146* [71]; S64 NATOXAQ *130* [201, 202]; S91 CECTOYS *126* [203, 204]; S68 HSDBTPS *101* [174, 205]; S86 TATTOOINK *98* [206–208]; S41 CCL4 *96* [209, 210]; S83 CCL5 *96* [211, 212]; S52 THSMOKE *95* [213]
(F) < 95	S95 PFASANEXCH *94* [214, 215]; S63 UBADWGW *84* [176, 216]; S59 NPINSECT *83* [217]; S67 TBUTYLPHENOLS *77* [218]; S97 UBABPAALT *71* [219, 220]; S88 UBABIOCIDES *62* [221–223]; S93 CECMOUTHING *60* [203, 204]; S40 ALGALTOX *54* [224]; S20 BISPHENOLS *52* [225, 226]; S81 THSTPS *52* [227]; S45 SYNTHCANNAB *39* [63]; S90 ZEROPMBOX1 *38* [228, 229]; S44 STATINS *18* [230]; S98 TIRECHEM *16* [231]

Full details given in Additional file 1 and Additional file 2 [81, 82]

Figure 4 and Table 1 show the number of entries in each NORMAN-SLE list as present on the NORMAN-SLE website and in the latest versions on the NORMAN-SLE Zenodo collection as of May 2022. The number of InChIKeys associated with these lists (as of May 2022) is available in Additional file 1 [81]. Additional file 1 also includes the number of entries included in PubChem (obtained via the PubChem NORMAN-SLE Tree [74]) and CompTox (via both the CompTox Chemical Lists [232] website as well as via the PubChem EPA DSSTox Tree [233], since the latter can be automated). These statistics were compiled on 4 May 2022. The corresponding files and code are available at the ECI NORMAN-SLE GitLab repository [234] in the “stats” subfolder. Note that the addition of new content to the NORMAN-SLE was put on hold during compilation of this manuscript (May and June 2022), to ensure that the results included here are internally consistent. All statistics presented here reflect the data in this state. Updates resumed 28 June 2022 and will be described in later efforts (see “Future updates” below).

### Summary statistics of the NORMAN-SLE

A selection of summary statistics and facts for the NORMAN-SLE is given in Table 2. Both the list and citation information were summarized on 4 May 2022 and the NORMAN-SLE PubChem numbers on 12 May 2022. The (cumulative) numbers of unique views and downloads collected from the NORMAN-SLE Zenodo community on 28 April 2022 are summarized in Table 3, along with the citation numbers for all lists and for the 5 most popular lists according to unique views. The “total unique compounds” number indicates how many entries have a defined chemical structure in PubChem, i.e., a PubChem CID. The “total live substances” number indicates how many entries are deposited, i.e., with a PubChem SID. The total number of unique compounds in PubChem is currently larger than S0 SUSDAT due to the different timing associated with the release cycle of NORMAN SusDat (the basis for S0 SUSDAT), as well as differences in the mappings of structures to unique identifiers. Future efforts will aim to close this time gap between NORMAN-SLE and NORMAN SusDat (see “Future updates” below). The data files supporting these statistics, including a breakdown of the DOIs of the citing articles, are archived on the ECI NORMAN-SLE GitLab pages [234] (“stats” subfolder) and are available as Additional file 3 [235] (views, downloads, citations per list) and Additional file 4 [236] (more detailed citation breakdown).

**Table 2:** Selected overall summary statistics for the NORMAN-SLE, compiled in May 2022

Category	Number	Comment
Total number of lists	99	S0 to S98
Total unique compounds	115,248	From PubChem NORMAN-SLE Tree [74]
Total live substances	117,071	From PubChem NORMAN-SLE Data Source Page [73]
Total live annotations	21,114	From PubChem NORMAN-SLE Data Source Page [73]
Largest list (# entries)	109,631	S0 SUSDAT
Smallest list (# entries)	16	S98 TIRECHEM
Total list citations	40	From NORMAN-SLE Zenodo Community [53]

Further details are given in the “stats” subfolder of the ECI NORMAN-SLE GitLab repository [234]. Total unique compounds = CID count; total live substances = SID count, # entries = number of entries (i.e., rows) in the SLE lists

**Table 3:** Unique views, downloads and citations for all NORMAN-SLE lists and the Top 5 lists (by unique views), according to the NORMAN-SLE Zenodo Community [53]

List	Code	Unique views	Unique downloads	Citations
Top 5 Lists (sorted by unique views)				
S13	EUCOSMETICS [108]: Cosmetics	10,429	9088	2
S60	SWISSPEST19 [129]: Pesticides	2440	2316	3
S72	NTUPHTW [147]: Pharmaceuticals	2278	2083	0
S73	METXBIODB [110]: BioTransformer data	2043	503	2
S0	SUSDAT [69]: Merged database	1625	1858	6
Total values				
All	Totals over all lists	42,358	53,651	40

Statistics compiled on 28 April (views/downloads) and 5 May (citations) 2022. The corresponding raw data are given in Additional file 3: Table S3 [235] and on the ECI NORMAN-SLE GitLab pages [234]

In total, 24 of the SLE lists have citations listed in Zenodo, with 40 citations from 19 articles. A full breakdown is given in Additional file 4 [236]. Of these 19 articles, 12 can be considered “internal”, i.e., articles written by authors involved with the NORMAN-SLE, including 5 articles describing SLE datasets [59, 118, 149, 154, 174] and 7 others citing SLE lists [24, 142, 237–241], while 7 articles are external [242–248]. Of the 24 lists cited, 6 lists are cited by external authors: S0 SUSDAT, S13 EUCOSMETICS, S14 KEMIPFAS, S25 OECDPFAS, S46 NTPFASREV19 and S75 CyanoMetDB.

### NORMAN-SLE PubChem integration

**Fig. 5:**
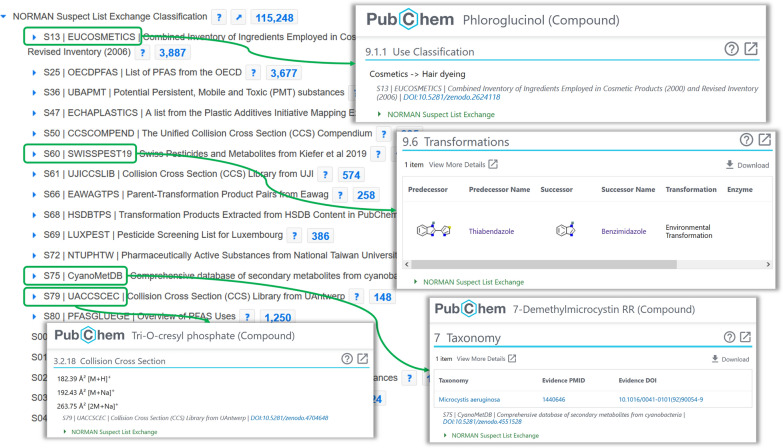
A collage of NORMAN-SLE content in PubChem. Left/back: the PubChem NORMAN-SLE Tree, with entries containing detailed classifications at the top, indicated by the blue arrows. Insets: selected annotation content (Use Classification, Transformations, Taxonomy and Collision Cross Section), linked to the corresponding source list via the green boxes and arrows. Screenshots taken 30 May 2022 (taxonomy on 16 June 2022)

As described above, the NORMAN-SLE content has been integrated into PubChem in a variety of ways. The basis of all further integration is the substance depositions, formed from the compilation of all lists as described in the Methods section. As of 12 May 2022, the substance deposition in PubChem included 117,071 substances (i.e., with PubChem SIDs), mapping to 115,248 unique PubChem CIDs according to the compiled CIDs at the top of the PubChem NORMAN-SLE Tree [74] (see also Table 2). All lists except S18 TSCASURF (for which no InChIKeys are available) are included in the numerically ordered set of lists on the PubChem NORMAN-SLE Tree. As of 30 April 2022, additional detailed classification breakdowns were available for S13 EUCOSMETICS [108], S25 OECDPFAS [60], S36 UBAPMT [54], S47 ECHAPLASTICS [170], S50 CCSCOMPEND [121], S60 SWISSPEST19 [129], S61 UJICCSLIB [150], S66 EAWAGTPS [163], S68 HSDBTPS [205], S69 LUXPEST [175], S72 NTUPHTW [147], S75 CYANOMETDB [119], S79 UACCSCEC [187] and S80 PFASGLUEGE [124]. Detailed classification content for S77 FCCDB [89] is already drafted on the test site. A screenshot of the top portion of the PubChem NORMAN-SLE Tree is shown on the left in Fig. 5. The collision cross section (CCS) content (S50 CCSCOMPEND [121], S61 UJICCSLIB [150] and S79 UACCSCEC [187]) has also been merged and extended in the “Aggregated CCS Classification” tree on PubChem to combine this with the data from CCSbase [249, 250] and to allow browsing by adduct categories across all datasets [251]. All datasets mentioned here can be accessed via hyperlinks available at the NORMAN-SLE Data Source page on PubChem [73]. Documentation on how to access the data integrated within PubChem is provided on the ECI GitLab pages, including how to find MS [252] and CCS [253] data for NORMAN-SLE lists via PubChem. This also includes code to retrieve the CCS data [254], along with a compiled archive of all CCS values in PubChem (7 June 2022) on Zenodo [255].

In addition to the deposition and classification, extensive annotation content (i.e., expert knowledge) provided within the NORMAN-SLE lists has been integrated within PubChem. Various pieces of information from NORMAN-SLE lists now appear on the individual compound records for 21,114 compounds (12 May 2022), with several examples shown as insets in Fig. 5. While the presence of this annotation information in text form in individual PubChem records is useful for readers of the individual chemical records, it also helps in search engine optimization (SEO), i.e., the discovery of this information in generalized search engines, beyond the original database. Some categories (PubChem headings indicated in italics) relate to the chemical role, e.g*.,* the “*ATC Code*” for pharmaceuticals (from S66 EAWAGTPS [163] and S76 LUXPHARMA [155]), “*Agrochemical Category*” (S66 EAWAGTPS [163] and S69 LUXPEST [175]), or “*Chemical Classes*” (S75 CyanoMetDB [119]). Information in the “*Use Classifications*” and “*Uses*” categories come from S13 EUCOSMETICS [108], S25 OECDPFAS [60], S47 ECHAPLASTICS [170], S60 SWISSPEST19 [129], S66 EAWAGTPS [163], S69 LUXPEST [175], S72 NTUPHTW [147], S79 UACCSCEC [187] and S80 PFASGLUEGE [124]. The composite “*Molecular Formula*” representation in S80 PFASGLUEGE [124] is also integrated. Taxonomy information (for organisms) has been included under the “*Taxonomy*” heading for compounds present in S75 CyanoMetDB [118, 119] and S29 PHYTOTOXINS [132] from the Toxic Plants-Phytotoxins database [131], and also appears on the individual taxa pages.

*Transformations* for 5135 CIDs have been added from the datasets S60 SWISSPEST19 [129], S66 EAWAGTPS [163], S68 HSDBTPS [205], S73 METXBIODB [110], S74 REFTPS [71], S78 SLUPESTTPS [173] and S79 UACCSCEC [187], as described in some of the articles mentioned above [24, 174, 241]. As a part of this, SEO text snippets describing these relationships have been added to the following headings: *Metabolism/Metabolites* (S73 METXBIODB [110] and S82 THSTPS [227]), *Drug Transformations* (S66 EAWAGTPS [163]) and *Agrochemical Transformations* (S60 SWISSPEST19 [129], S66 EAWAGTPS [163] and S78 SLUPESTTPS [173]). An example *Transformations* entry is provided in the middle right inset in Fig. 5. The *Transformations* data are compiled and archived on GitLab [80] and Zenodo [256], and is integrated in patRoon 2.0 [257], an open source software for mass spectrometry based non-target analysis that includes suspect and transformation product screening workflows.

Finally, a significant amount of experimental data has also been included in PubChem from NORMAN-SLE contributors. MS/MS and NMR data have been included from several transformation products (TPs) and/or parent compounds of contaminants of emerging concern, including: ^13^C NMR, ^19^F NMR, ^1^H NMR, ^31^P NMR, MS/MS (all from S74 REFTPS [71] containing MS/MS data extracted from 4 articles [258–262] and NMR data from 1 article [258]). Many of these CIDs were not available in PubChem previously. Measured CCS values (often for multiple adducts) associated with 1579 CIDs are included in PubChem, from the datasets S50 CCSCOMPEND [121], S61 UJICCSLIB [150] and S79 UACCSCEC [187] (see also inset at the bottom left in Fig. 5). As mentioned above, this data can be retrieved from PubChem, with documentation provided on the ECI GitLab pages [252–254], along with an archive of the CCS data on Zenodo [255].

## Discussion

### NORMAN-SLE coverage

The NORMAN-SLE (https://www.norman-network.com/nds/SLE/) provides users with simple access to suspect lists. These lists are then integrated into the merged NORMAN SusDat collection (https://www.norman-network.com/nds/susdat) in the so-called “MS-ready” [263] form (ready for mass spectral screening, i.e., desalted, neutralized, etc*.*) with a searchable summary table containing NORMAN-relevant additional properties such as fragmentation information, retention time indices [238] and predicted toxicity values [264]. Over the seven years since the launch of the NORMAN-SLE, the website has grown from hosting a handful of lists to now hosting 99 formal referenced collections, amounting to information on 117,071 substances and 115,248 unique compounds (see Table 2). While these total numbers represent only 0.1% of PubChem contents, it is approximately 12% the size of CompTox, i.e., a significant portion of openly available data on environmentally relevant chemicals. Approximately 43,300 CIDs associated with the NORMAN-SLE lists are not yet available in CompTox lists (calculated by overlapping the PubChem NORMAN-SLE and US EPA DSSTox trees on 31 May 2022; documented here [265]). A large proportion of these CIDs missing in CompTox come from the European market lists S32 REACH2017 [86] from the REACH regulation and S17 KEMIMARKET from the Swedish Chemicals Agency (KEMI) [68], as well as from S71 CECSCREEN [85]. It is important to note the discrepancy between the NORMAN-SLE and CompTox versions of NORMAN-SLE lists, especially if the European-relevant chemicals are the focus of suspect screening efforts. This discrepancy results, in part, from the fact that it has been challenging to verify the identities of a large number of the REACH chemicals; many of these are also missing from the PubChemLite collection due to a lack of additional annotation content [241]. Of the 115,248 CIDs integrated in PubChem, 6275 CIDs come exclusively from the NORMAN-SLE (31 May 2022). This highlights that several NORMAN-SLE lists provide valuable data that is not otherwise available in the open domain, including, e.g*.,* mycotoxins that are not commercially available, but have been isolated via fungal fermentation and purification (S26 MYCOTOXINS [200]), as well as newly published PFAS and TPs added via the S46 PFASNTREV19 [46], S74 REFTPS [71] and S96 ECIPFAS [72] lists (among others).

An overview of the number of regulatory lists and major topics is given in Table 4. Key topics include pharmaceuticals, toxins, pesticides, PFAS, TPs, plastics, priority lists, surfactants, and suspect lists for water, with 16 lists coming from European regulatory authorities. Future topics are discussed below.

**Table 4:** NORMAN-SLE lists (given by suspect list “S” number only for readability) associated with various topics and sources

Topic	Total	Lists	Notes
Regulatory	16	S14, S17, S20, S23, S28, S30, S32, S36, S39, S47, S54, S63, S67, S86, S88, S97	Includes data from ECHA, EFSA, KEMI, REACH, UBA
Pharmaceuticals	11	S6, S10, S44, S45, S55, S56, S57, S58, S72, S76, S92	Excludes personal care products (S13)
Toxins	11	S24^, S26*, S29*, S37^, S40*, S43^, S52, S58, S64*, S75*, S85*	Includes *natural toxins, ^neurotoxins and others
PFAS	10	S9, S14, S25, S46, S80, S89, S92, S94, S95, S96	
TPs	10	S8, S11, S38, S66, S68, S73, S74, S78, S79, S81	
Water	9	S2, S5, S36*, S39, S41, S63, S64, S82*, S84*	Includes *PMT lists
Pesticides	7	S11, S28, S59, S60, S69, S88, S94	
Nat. products	7	S26, S29, S40, S59, S64, S75, S85	
Plastics	6	S20, S47, S48, S49, S67, S97	Plastics/chemicals associated with plastics
Priority	5	S15, S16, S36, S54, S63	Priority monitoring lists
Surfactants	4	S7, S8, S18, S23	

PMT: persistent, mobile, toxic substances. Nat. products: natural products. Other abbreviations: see abbreviation listing

### Recognition, role and use of the NORMAN-SLE

The collection of download and view statistics on Zenodo, along with citation tracking, has helped track the impact of the NORMAN-SLE on the community, as shown in Tables 2 and 3. Since the Zenodo integration only commenced in 2019, these statistics only cover a fraction of the real-world use of the NORMAN-SLE. Several efforts known to the authors that build on NORMAN-SLE information are not captured within these statistics, including for instance CECSCREEN [84], which retrieved much of the NORMAN-SLE data that was integrated into CECSCREEN via CompTox. While a PubMed query on the NORMAN-SLE and the sub-collections was attempted to discover more citing articles, this did not return sufficiently reliable results for further interpretation (various text queries generated large numbers of false positives without finding true positives); it seems that environmental literature is not sufficiently covered in PubMed. Guidance is now provided on the NORMAN-SLE website to help users correctly cite the works; it is hoped that this publication will also help to raise awareness of the resource for the wider scientific community—and will highlight the necessity to cite contributions, so that the level of community adoption becomes more visible over time.

The unique views, downloads, and citations available on Zenodo revealed some surprising results. While in NORMAN much focus was given to pesticides, pharmaceuticals, REACH registered chemicals and TP lists due to popular demand, the most popular list by far (see Table 3) proved to be S13 EUCOSMETICS [108], a collection of chemicals employed in cosmetics from EU regulations [106, 107]. The second most viewed list was a Swiss pesticide and metabolite list, S60 SWISSPEST19 [129], a quite recent collection by Kiefer et al*.* [128] from Eawag, which was expected to gain significant attention. This was an updated version of S11 SWISSPEST [189] from Moschet et al. [12]. While the NORMAN-SLE has several pharmaceuticals lists, the third most viewed list—a pharmaceuticals list, S72 NTUPHTW—was in fact a 2021 contribution from the National Taiwan University (Chen et al*.* [146]), which was received following a peer-review recommendation for submission to the NORMAN-SLE during manuscript revisions. This was the first such external contribution and marks a milestone in the NORMAN-SLE development. While S0 SUSDAT only appeared in 5th place according to views/downloads, these numbers are only a small fraction of the real statistics, since NORMAN SusDat is also available on a dedicated interactive website. This is also reflected in the relatively high citation count for SusDat compared with other lists. The NORMAN SusDat website (https://www.norman-network.com/nds/susdat/) was visited 120,221 times (20,258 times counting unique IP addresses per day) between 27 Feb. 2020 and 13 July 2022, compared with 26,318 visits to the NORMAN-SLE website (https://www.norman-network.com/nds/SLE/). The original versions of two highly popular lists, the Food Contact Chemicals database (FCCdb) and the database of Chemicals associated with Plastic Packaging (CPPdb) are also available on Zenodo. These have much higher views and (for FCCdb only) download statistics associated with their original depositions compared with the NORMAN-SLE version (which directs viewers back to the original resource with a request to cite the original dataset). The numbers (10 July 2022) are (unique views/downloads): CPPdb [103] (2,082/659), S48/S49 CPPDBLISTA/B [104, 152] (594/1041), FCCdb [88] (8,612/3,703), S77 FCCDB [89] (410/398). Neither of these original depositions have any citations. The reason for the parallel integration of these lists (i.e., an original version plus NORMAN-SLE version) is to ensure the maintenance of the full integration with the NORMAN-SLE website, PubChem and CompTox (as these require the preparation and archive of additional files, as well as the ability to edit the depositions and make any necessary adjustments).

All NORMAN-SLE lists feed into the merged collection NORMAN SusDat, which forms the basis of the NORMAN Database System (NDS) [29, 31] and integration into other NORMAN initiatives such as the Digital Sample Freezing Platform (DSFP) [266] and prioritization efforts (see Fig. 2). Several NORMAN-SLE lists are associated with NORMAN activities such as collaborative trials [4, 6] and NormaNEWS [184, 199]. NORMAN SusDat and the DSFP are used extensively in many studies in Europe (e.g., [142, 237, 240]*,*), many of which are still in the process of being published. Beyond NORMAN activities and the statistics presented above, gauging the impact of the NORMAN-SLE remains rather intangible at present, since much of it also relates to the use of NORMAN SusDat. Anecdotally, the efforts behind the S11 SWISSPEST and S60 SWISSPEST19 lists have led to the inclusion of more compounds in the (Swiss) national monitoring program [267, 268], while the efforts related to S2 STOFFIDENT have resulted in the discovery of new P-containing compounds (unpublished results).

### FAIR data and chemical curation

The decision to deposit the NORMAN-SLE collections on Zenodo helped “FAIRify” [22, 23, 269] the NORMAN-SLE via the provision of DOIs and versioning control. This helps trace updates and provide static URLs to data files, enabling powerful automatic integration such as that currently performed with PubChem (see Fig. 2), as well as providing the citation possibilities and statistics presented above. These are all features that are not currently possible via the infrastructure supporting the NORMAN-SLE website. Version control is important to track changes to the lists; not only in terms of fixing errors (i.e., curation), but also to keep historical records of lists as they change, since some chemicals that have, e.g*.,* been phased out in the EU or changed in relevance may still occur in imported products and the environment. Overall, the data in the NORMAN-SLE is currently reasonably FAIR: *Findable* via the DOI and InChIKey for deep indexing; *Accessible* via the download options of Zenodo; *Interoperable* via the use of SMILES and InChI; and *Reusable* via the open license (CC-BY 4.0) and the use of community standards where feasible, exemplified by the PubChem integration. A transition to the standardized templates proposed recently [24, 37, 270] will help FAIRify the NORMAN-SLE further; these templates could also form the basis to help propose a set of chemical identifiers needed to establish unique (chemical) identifiers for the future European Open Data Platform.

While best efforts are made to map NORMAN-SLE contributions to identifiers correctly, the resources are not available for extensive curation efforts such as those performed by CompTox. This is coupled with the current “as is” philosophy, where lists are processed to best represent the data as provided. The versioning offered by Zenodo opens options for quality control and updating of lists, however this is still a very manual process and currently decoupled from updates to NORMAN SusDat—workflow and infrastructure upgrades to resolve this are underway. Since NORMAN-SLE lists are both sourced from and deposited to third party systems, and due to the different release cycles (PubChem updates daily, CompTox approximately annually), different versions of the data result—which can cause confusion. A coherent collaborative and timely process to update and circulate updated lists across the various systems would be beneficial; while this currently works well with the automated updates between PubChem and the NORMAN-SLE, it is not yet possible with CompTox.

As mentioned above, the NORMAN-SLE hosts 99 suspect lists, which are then integrated into the merged NORMAN SusDat collection in the so-called “MS-ready” [263] form (ready for mass spectral screening). Access to “MS-ready” suspect lists [263] is urgently needed to reduce the number of trivial mistakes in suspect screening (such as searching for the exact masses of salts or polymers). However, the fact that many NORMAN-SLE lists contained both the original substances and their MS-ready form caused several problems with the PubChem integration and the subsequent mapping of structures to the expert knowledge contained within the lists (e.g*.,* it is unclear to an automated method which structure is associated with the metadata: the original SMILES, or the MS-ready SMILES form). The integration of NORMAN-SLE content in PubChem and CompTox, along with discussions with developers, contributors and users is helping to develop better solutions to some of the challenges associated with the mapping of various chemical forms over time.

Basic cheminformatics limitations still prevent the complete integration of suspect information, such as dealing with undefined structures for which no InChI or InChIKey exists (e.g*.,* isomeric mixes such as surfactants, where several structures are hidden behind one detected “mass”). Taking examples from biocides, UVCBs of interest include: creosote; reaction products of 5,5-dimethylhydantoin, 5-ethyl-5-methylhydantoin with bromine and chlorine (DCDMH); reaction products of paraformaldehyde and 2-hydroxypropylamine (ratio 1:1); or reaction products of: glutamic acid and N-(C12-C14-alkyl)propylenediamine (Glucoprotamin). For those examples, mixture indicators or marker compounds associated with the UVCB may help evaluate these compounds. Biocidal polymers include “polyhexamethylene biguanide hydrochloride with a mean number-average molecular weight (Mn) of 1415 and a mean polydispersity (PDI) of 4.7 (PHMB(1415;4.7))” or “Polymer of formaldehyde and acrolein” or “Polymer of NMethylmethanamine (EINECS 204-697-4 with (chloromethyl) oxirane (EINECS 203-439-8)/Polymeric quaternary ammonium chloride (PQ Polymer)”, where pyrolysis GC–MS may assist analysis (not yet an explicit focus of the NORMAN-SLE lists). The CompTox team has made some efforts to address cases such as these through the definition of “related structures” and PubChem have released “concepts” to group several compounds related to substances under a given concept name, a topic that will be explored further at the upcoming BioHackathon [271]. The definition of chemical identifiers such as an InChI(Key) describing UVCB substances is highly desirable to ensure that these efforts can be automated. While initial efforts such as the mixture InChI (MInChI) show promise (see e.g*.,* Fig. 3 in [51]), there is room for further developments. Organometallic compounds (e.g., methylmercury compounds, organolead/organotin compounds, cyclic volatile methylsiloxanes, gadolinium compounds used as contrast agents) are cases that can be handled to an extent with the current approaches (although not in an “MS-ready” form). Upcoming InChI developments will hopefully improve the handling of organometallic species in databases in the near future [272]. Further examples related to biocides that are currently beyond the scope of the NORMAN-SLE (but are in part covered by the NDS) include microbial preparations or strains used as biocidal products, where metabarcoding or proteomics (peptide biomarkers) could be used for characterization, along with nanomaterials/nanoplastics.

### Future updates: new submissions

As described above, submissions and updates to the NORMAN-SLE were frozen during preparation of this manuscript. In the meantime, both new submissions and expressions of interest to update existing lists have been registered, partially stimulated by reaching out to all contributors during the writing of this work. Updates have been suggested for S17 KEMIMARKET [68], S28 EUBIOCIDES [196] with information from ECHA [273], S34 EXPOSOMEXPL [165, 166] with new data from [274] plus new microbial metabolites [275, 276] and S75 CyanoMetDB [118, 119] (next release due early 2023). Suggestions for new contributions include a list of endocrine disruptors within the activities of PARC, the Proposition 65 (Prop-65) list of chemicals from the California EPA [277], Phenol-Explorer [278–280], the Database on Migrating and Extractable Food Contact Chemicals (FCCmigex) [281], and finally a shale gas suspect list [282] that has been applied in other studies: [283, 284] and will fill a long-identified gap with respect to fracking-related content.

Beyond these new suggested submissions, future developments involve improving the current submission system to the NORMAN-SLE. The current submissions generally rely on personal contacts, with only one submission recommended externally so far (S72 NTUPHTW [147]). Manual work for the NORMAN-SLE team would be reduced if contributors would consider using a template, as described recently [24, 37, 270]. While the evolution of openly available batch services offered by PubChem [40] and CompTox [41] have greatly eased the mapping of contributed lists to include the required information for upload, a further semi-automation of this workflow would ease matters further and is already in planning. However, extensive curation based on CAS as performed by CompTox is currently out of scope of the NORMAN-SLE, which is based on fully open access resources. While a feedback loop between CompTox and the NORMAN-SLE would help the NORMAN-SLE benefit from the CompTox curation, this is not currently possible. A submission system such as that offered by PubChem could be considered in the future, but is currently beyond reach of the resources available for the NORMAN-SLE. While these enhancements would be desirable, overall the current system has held up well for 99 lists so far and more contributions are welcomed by emailing the NORMAN-SLE team as detailed on the website: https://www.norman-network.com/nds/SLE/.

### Future updates: potential new features

Beyond the new submissions and processing updates mentioned in the previous section, several new features have been suggested (and are being considered) for the NORMAN-SLE and/or the broader NORMAN Database System. These can be grouped into four major areas reflected in the following paragraphs: experimental, TPs, categorization/use and regulatory.

On the experimental side, additional functionality to account for physical chemical properties such as mass, polarity, likely ionization mode and amenability to either GC or LC would be beneficial, along with the link to available MS/MS data and/or reference standards for further confirmation. This information is included to a large extent in NORMAN SusDat, which provides a centralized access point for this information, along with predicted toxicity values [264] and retention indices [238], but will be streamlined and automated further, also to account for possibilities arising from the PubChem integration. Documentation on how to obtain some of this information via PubChem is also available, e.g., for MS/MS [252] and CCS values [253–255]. Advanced Entrez queries (via PubChem) can be used to limit this to certain measurement modes. Another suggested enhancement related to UVCBs would be to include important substructures such as the head group of surfactants or repeating unit of polymers, which could be linked to MS/MS fragments.

A large focus has been placed on TPs over the recent years. A continuation of ongoing efforts will include adding more TPs, including the extraction of data from literature to fill data gaps [71, 174, 205] and the integration of workflows in patRoon [257] in a manner compatible with other NTS workflows. Over the years, there has been increasing interest to add lists of predicted TPs to the NORMAN-SLE, with submissions including predicted TPs for S6 ITNANTIBIOTICS [159], S71 CECSCREEN [85] (both generated with BioTransformer [111]) and S38 SOLNSLMCTPS [102]. While such lists are valuable for researchers performing NTS, these can cause problems with downstream integration with the NDS, CompTox and PubChem as these predicted structures are not necessarily observed and verified, while the number of entries can be an order of magnitude higher (or more) than the source list. These datasets are generally decoupled from the cross-integration at present. A future discussion for NORMAN will be how best to integrate predicted TP data, with the possibility of a “Transformations” module to be added—potentially to represent both documented transformations (e.g., similarly as shown in the insets in Figs. 2 and 5) and predicted transformations.

As the NORMAN-SLE list numbers climb, and with several contributions covering related topics (see Table 4), further refinements will be needed to group lists together and allow the selection of certain subsets for different use cases, or the sorting of lists by categories. The extensive integration with PubChem and the resulting need for organization of NORMAN-SLE content in both CompTox and PubChem has given rise to categorization and classification efforts, and preliminary functionality allowing this is already integrated into NORMAN SusDat. Since there is great interest in the gathering of “Use” information and categorization in general, NORMAN has already initiated activities within the Prioritization working group [285] to define and collect relevant use information and categories from members. These activities will feed into subsequent future developments within NORMAN, PARC [28, 29], EU projects such as ZeroPM [229] and beyond.

The NORMAN-SLE is a community resource built on an incredible amount of volunteer effort and rather limited financial resources. The entire NDS is supported through the NORMAN Association and project funding obtained by individual contributors. The integration with external resources such as PubChem, CompTox and Zenodo provides significant added value beyond the capabilities available to NORMAN. This approach is key to foster cooperation among existing regulatory frameworks, helping to share data and improve chemical risk assessment in the shift towards a “one substance, one assessment” paradigm [286]. With the EU strongly supporting Open and FAIR data, including large initiatives such as PARC [28, 29] and EIRENE [30], along with Green Deal projects such as ZeroPM [229], opportunities for further developments, consolidation and harmonization with broader EU efforts, including the future Open Data Platform appear promising. While the idea behind the NORMAN-SLE has broad support, the current infrastructure and personnel could not currently support, for instance, a requirement to host and thus make all European environmental research data Open and FAIR. If, however, the experiences in building the NORMAN-SLE could help contribute towards establishing such a platform (to which the NORMAN-SLE could contribute), this would be a huge benefit for research and researchers.

## Conclusions

The NORMAN Suspect List Exchange (NORMAN-SLE) was created to provide a service to NORMAN members and the greater scientific community, in response to a clear need identified in the NORMAN Non-target Collaborative Screening Trial [4]. Through the provision of a centralized website to collect various suspect lists and references, information exchange is ensured to apply the “screen smart” strategy on specific scientific questions. This FAIRified resource is archived on Zenodo to give DOIs for each set, allowing the cross-integration with other resources and formal citation of datasets, raising the profile of the research of various contributors. The combined list formed from all NORMAN-SLE contributions, NORMAN SusDat, serves as a basis for chemical management for the entire NORMAN Database System (NDS), including the NORMAN Digital Sample Freezing Platform (DSFP) [266].

The NORMAN-SLE is not intended to replace major open compound databases such as ChemSpider, PubChem or CompTox, but rather offers a specialized, complementary service targeted to the environmental science community, particularly in relation to suspect screening, for integration within these larger resources, as done with CompTox and PubChem. Raising the awareness about relevant suspect screening lists and the quality issues surrounding suspect screening is vital for improving the identification of contaminants of emerging concern in the environment, biota, and products, thereby helping to reduce the number of molecular unknowns in mass spectrometry analyses and to facilitate more comprehensive chemicals assessments. The NORMAN-SLE welcomes new submissions of suspect lists within the scope, along with other ideas and feedback, as described on the NORMAN-SLE website (https://www.norman-network.com/nds/SLE/).

## Supplementary Information

**Additional file 1:** Summary of the NORMAN-SLE datasets (CSV format) as of 4 May 2022 [81].

**Additional file 2:** Overview of the NORMAN-SLE website (DOCX format) as of 30 May 2022 [82].

**Additional file 3:** Summary of Zenodo view and download statistics, plus citations (CSV format) as of 28 April 2022 [235].

**Additional file 4:** Summary of Zenodo citations plus DOIs per list (CSV format) as of 1 May 2022 [236].

**Additional file 5:** Authorship contributions and acknowledgements mapped to NORMAN-SLE lists (XLSX format).

## Data Availability

All data integrated in the NORMAN Suspect List Exchange are available from the NORMAN-SLE website (https://www.norman-network.com/nds/SLE/) and on the Zenodo NORMAN-SLE community website (https://zenodo.org/communities/norman-sle) or via the individual DOIs (see Table 1). The merged NORMAN SusDat collection is also available (https://www.norman-network.com/nds/susdat/). Individual lists can be accessed by their code on CompTox, the collection can be found under this search URL (https://comptox.epa.gov/dashboard/chemical-lists?search=NORMAN) or on the NORMAN-SLE website (https://www.norman-network.com/nds/SLE/). The NORMAN-SLE is available as data source in PubChem (https://pubchem.ncbi.nlm.nih.gov/source/23819) and browsable as a classification tree (https://pubchem.ncbi.nlm.nih.gov/classification/#hid=101). Detailed annotation content is available in several PubChem compound records, with an overview on the Data Source page (https://pubchem.ncbi.nlm.nih.gov/source/23819). The code supporting the NORMAN-SLE including documentation is available on GitLab (https://gitlab.lcsb.uni.lu/eci/NORMAN-SLE/), along with the code supporting the NORMAN-SLE/PubChem integration (https://gitlab.lcsb.uni.lu/eci/pubchem).

## Abbreviations

ASNT: Abstract Syntax Notation (ASN.1) Text format
ATC: Anatomical Therapeutic Chemical code
CAS: Chemical Abstract Service
CCS: Collision cross section (ion mobility experiments)
CDK: Chemistry Development Kit
CECs: Contaminants of Emerging Concern
CID: PubChem Compound Identifier
CPPdb: Chemicals associated with Plastic Packaging database
CSID: ChemSpider Identifier
CSV: Comma Separated Values
DOI: Digital Object Identifier
DSFP: Digital Sample Freezing Platform
DSSTox: Distributed Structure-Searchable Toxicity (database)
DTXSID: Distributed Structure-Searchable Toxicity (DSSTox) substance identifier
EC: European Commission
ECHA: European Chemicals Agency
ECI: Environmental Cheminformatics group, University of Luxembourg
EFSA: European Food Safety Authority
EIRENE: Environmental Exposure Assessment Research Infrastructure
EU: European Union
FAIR: Findable, Accessible, Interoperable, Reusable
FCCdb: Food Contact Chemicals database
FCCmigex: Database on Migrating and Extractable Food Contact Chemicals
GC: Gas chromatography
HRMS: High resolution mass spectrometry
InChI: International Chemical Identifier
InChIKey: Hashed form of the International Chemical Identifier
IP: Internet Protocol
JRC: Joint Research Centre
JSON: JavaScript Object Notation
KEMI: Swedish Chemicals Agency
LC: Liquid chromatography
MInChI: Mixture InChI
MS: Mass spectrometry
MS/MS: Tandem mass spectrometry
NDS: NORMAN Database System
NMR: Nuclear magnetic resonance
NORMAN: Network of reference laboratories, research centres and related organisations for monitoring of emerging environmental substances
NORMAN SusDat: NORMAN Substance Database
NORMAN-SLE: NORMAN Suspect List Exchange
NTS: Non-Target screening
PARC: European Partnership for Chemicals Risk Assessment
PFAS: Per- and polyfluoroalkyl substances
PMT: Persistent, mobile and toxic substances
REACH: Registration, Evaluation, Authorisation and Restriction of Chemicals (EU regulation)
SDF: Structure Data Format
SEO: Search Engine Optimization
SID: PubChem Substance Identifier
SMILES: Simplified Molecular-Input Line-Entry System
TPs: Transformation products
UBA: German Environment Agency (Umweltbundesamt)
US EPA: United States Environmental Protection Agency
UVCBs: Substances of Unknown or Variable Composition, Complex Reaction Products or Biological Materials
XML: Extensible Markup Language
ZeroPM: Zero Pollution of Persistent, Mobile Substances (EU project)
