# Supplementary material for: The NORMAN Suspect List Exchange (NORMAN-SLE): facilitating European and worldwide collaboration on suspect screening in high resolution mass spectrometry
Source: Environ Sci Eur. 2022 Oct 21;34(1):104. doi: 10.1186/s12302-022-00680-6 (PMC9587084; doi:10.1186/s12302-022-00680-6)
Supplement: Supplementary file 2 — Additional file 2: Overview of the NORMAN-SLE website (DOCX format) as of 30 May 2022 [82]. [file 12302_2022_680_MOESM2_ESM.docx]

| **No.** | **Abbreviation** | **Description** | **Link to full list** | **Link to InChIKey list** | **References** |
| --- | --- | --- | --- | --- | --- |
| S0 | SUSDAT | **Merged NORMAN Suspect List: SusDat** | [Interactive Data table](https://www.norman-network.com/nds/susdat/)  SusDat with Haz and Expo scores as [XLSX](https://www.norman-network.com/sites/default/files/files/suspectListExchange/010322Update/susdat_2022-01-18-104316.xlsx), [CSV](https://www.norman-network.com/sites/default/files/files/suspectListExchange/010322Update/susdat_2022-01-18-104316.csv) (18/01/2022)  [MetFrag CSV](https://www.norman-network.com/sites/default/files/files/suspectListExchange/030320Update/NORMANSusDat_20Nov2019_wExpoHaz.csv) (03/03/2020)  CompTox [SUSDAT List](https://comptox.epa.gov/dashboard/chemical_lists/susdat) | SusDat InChIKeys: [All](https://www.norman-network.com/sites/default/files/files/suspectListExchange/010322Update/SusDat_InChIKeys_20220118.txt), [MS-ready](https://www.norman-network.com/sites/default/files/files/suspectListExchange/010322Update/SusDat_MSready_InChIKeys_20221801.txt) (18/01/2022) | A merged list of >111,000 structures from SLE suspect lists. See [interactive version](https://www.norman-network.com/nds/susdat/). Compiled by Reza Aalizadeh, Nikiforos Alygizakis and Lubos Cirka, University of Athens/EI, including RTI and toxicity values, with Hazard and Exposure values provided by Stellan Fischer, KEMI, documented [here](https://www.norman-network.com/sites/default/files/files/suspectListExchange/170320Update/MarketList_Documentation_16March2020.docx). DOI: [10.5281/zenodo.2664077](https://doi.org/10.5281/zenodo.2664077) |
| S1 | MASSBANK | **NORMAN Compounds in MassBank** | [CSV](https://www.norman-network.com/sites/default/files/files/suspectListExchange/031017Update/MassBankEU_Cmpds_11042017_wMS_DTXSIDs_03102017.csv), [XLSX](https://www.norman-network.com/sites/default/files/files/suspectListExchange/031017Update/MassBankEU_Cmpds_11042017_wMS_DTXSIDs_03102017.xlsx) with Fragments (3/10/2017). CompTox [MassBank EU Reference List](https://comptox.epa.gov/dashboard/chemical_lists/massbankref), [MassBank EU Special Cases](https://comptox.epa.gov/dashboard/chemical_lists/massbankeusp), [Fragment Download](ftp://newftp.epa.gov/COMPTOX/Sustainable_Chemistry_Data/Chemistry_Dashboard/MassBank_FRAGMENT%20FILE.zip) | [MassBankEUInChIKeys](https://www.norman-network.com/sites/default/files/files/suspectListExchange/170619Update/MassBankEU_InChIKeys_17062019.txt) (17/06/2019) | [www.massbank.eu](http://massbank.eu/MassBank/) Stravs et al. 2013. DOI: [10.1002/jms.3131](http://onlinelibrary.wiley.com/doi/10.1002/jms.3131/full)  DOI: [10.5281/zenodo.2621390](https://doi.org/10.5281/zenodo.2621390) |
| S2 | STOFFIDENT | **HSWT/LfU STOFF-IDENT Database of Water-Relevant Substances** | STOFF-IDENT as [XLSX](https://www.norman-network.com/sites/default/files/files/suspectListExchange/180620Update/STOFF-IDENT_Cmpds_06092017.xlsx), [CSV](https://www.norman-network.com/sites/default/files/files/suspectListExchange/180620Update/STOFF-IDENT_Cmpds_06092017.csv) (18/06/2019)  CompTox [STOFF-IDENT List](https://comptox.epa.gov/dashboard/chemical_lists/stoffident) | [STOFF-IDENT InChIKeys](https://www.norman-network.com/sites/default/files/files/suspectListExchange/031017Update/STOFF-IDENT_06092017_InChIKeys.txt) (6/09/2017) | The database enables the search for exact masses from target or unknown lists and the automatic use of a Retention Time Index. See: <https://water.for-ident.org/#!home>. DOI: [10.5281/zenodo.2621451](https://doi.org/10.5281/zenodo.2621451) |
| S3 | NORMANCT15 | **NORMAN Collaborative Trial Targets and Suspects** | LC-MS: [CSV](https://www.norman-network.com/sites/default/files/files/suspectListExchange/031017Update/Targ_Sus_NT-wID_LC_final_31102016_wDTXSIDs.csv), [XLSX](https://www.norman-network.com/sites/default/files/files/suspectListExchange/031017Update/Targ_Sus_NT-wID_LC_final_31102016_wDTXSIDs.xlsx) (3/10/2017)  GC-MS: [CSV](https://www.norman-network.com/sites/default/files/files/suspectListExchange/031017Update/Targ_Sus_NT-wID_GC_final_31102016_wDTXSIDs.csv), [XLSX](https://www.norman-network.com/sites/default/files/files/suspectListExchange/031017Update/Targ_Sus_NT-wID_GC_final_31102016_wDTXSIDs.xlsx) (3/10/2017)  CompTox [NORMANCT15 List](https://comptox.epa.gov/dashboard/chemical_lists/normanct15) | [LC-MS InChIKeys](http://www.norman-network.com/sites/default/files/files/suspectListExchange/Targ_Sus_NT-wID_LC_final_InChIKeys_31102016.txt) (31/10/2016)  [GC-MS InChIKeys](http://www.norman-network.com/sites/default/files/files/suspectListExchange/Targ_Sus_NT-wID_GC_final_InChIKeys_31102016.txt) (31/10/2016) | Schymanski et al. 2015. DOI: [10.1007/s00216-015-8681-7](http://link.springer.com/article/10.1007/s00216-015-8681-7)  DOI: [10.5281/zenodo.2621478](https://doi.org/10.5281/zenodo.2621478) |
| S4 | UJIBADE | **University of Jaume I** | Bade et al [CSV](https://www.norman-network.com/sites/default/files/files/suspectListExchange/031017Update/Bade_etal_544Compounds_wInChIs_DTXSIDs.csv), [XLSX](https://www.norman-network.com/sites/default/files/files/suspectListExchange/031017Update/Bade_etal_544Compounds_wInChIs_DTXSIDs.xlsx) (3/10/2017)  CompTox [UJIBADE List](https://comptox.epa.gov/dashboard/chemical_lists/ujibade) | [Bade et al InChIKeys](https://www.norman-network.com/sites/default/files/files/suspectListExchange/Bade_etal_2015_544Compounds_InChIKeys.txt) (28/11/2015) | Bade et al 2015, Sci. Tot. Environ. 538: 934-941.  DOI: [10.1016/j.scitotenv.2015.08.078](http://dx.doi.org/10.1016/j.scitotenv.2015.08.078)  DOI: [10.5281/zenodo.2621916](https://doi.org/10.5281/zenodo.2621916) |
| S5 | KWRSJERPS | **KWR Drinking Water Suspect List** | KWR Suspects [CSV](https://www.norman-network.com/sites/default/files/files/suspectListExchange/031017Update/NormanTargetSuspects-KWR_withStruct_DTXSIDs.csv), [XLSX](https://www.norman-network.com/sites/default/files/files/suspectListExchange/031017Update/NormanTargetSuspects-KWR_withStruct_DTXSIDs.xlsx) (3/10/2017)  CompTox [KWRSJERPS List](https://comptox.epa.gov/dashboard/chemical_lists/kwrsjerps) | [KWR InChIKeys](https://www.norman-network.com/sites/default/files/files/suspectListExchange/NormanTargetSuspects-KWR_InChIKeys.txt) (15/03/2016) | Sjerps et al. 2016 Water Research 93: 254-264.  DOI: [10.1016/j.watres.2016.02.034](http://www.sciencedirect.com/science/article/pii/S0043135416300938)  DOI: [10.5281/zenodo.2621941](https://doi.org/10.5281/zenodo.2621941) |
| S6 | ITNANTIBIOTIC | **Antibiotic List: ITN MSCA ANSWER** | Antibiotic [CSV](https://www.norman-network.com/sites/default/files/files/suspectListExchange/031017Update/Antibiotics_ITN_MSCA_ANSWER_160616_wDTXSIDs.csv), [XLSX](https://www.norman-network.com/sites/default/files/files/suspectListExchange/031017Update/Antibiotics_ITN_MSCA_ANSWER_160616_wDTXSIDs.xlsx) (3/10/2017)  Antibiotics Step 1 CYP Metabolites [CSV](https://www.norman-network.com/sites/default/files/files/suspectListExchange/020522Update/ITNANTIBIOTIC_CYP_Metabolites_Step1.csv), [XLSX](https://www.norman-network.com/sites/default/files/files/suspectListExchange/020522Update/ITNANTIBIOTIC_CYP_Metabolites_Step1.xlsx) (02/05/2022)  CompTox [ITN ANTIBIOTIC List](https://comptox.epa.gov/dashboard/chemical_lists/itnantibiotic) | [Antibiotic InChIKeys](https://www.norman-network.com/sites/default/files/files/suspectListExchange/Antibiotic_ITN_MSCA_ANSWER_InChIKeys_160616.txt) (16/06/2016)  [Step 1 CYP Metabolites InChIKeys](https://www.norman-network.com/sites/default/files/files/suspectListExchange/020522Update/ITNANTIBIOTIC_CYP_Metabolites_InChIKeys.txt) (02/05/2022) | A list of antibiotics compiled by Nikiforos Alygizakis (EI/Uni Athens). See e.g. Paulus et al 2019 DOI: [10.1016/j.ijheh.2019.01.004](https://doi.org/10.1016/j.ijheh.2019.01.004)  Suspect list of human phase-I antibiotic metabolites, created with [BioTransformer](https://biotransformer.ca/) (v3.0.0) provided by Tim Jonkers (VU, NL). Jonkers et al (in prep.)  DOI: [10.5281/zenodo.2621956](https://doi.org/10.5281/zenodo.2621956) |
| S7 | EAWAGSURF | **Eawag Surfactants Suspect List** | Suspect formulas: [CSV](https://www.norman-network.com/sites/default/files/files/suspectListExchange/Surfactant_Suspects_Schymanski_etal_2014.csv), [XLSX](https://www.norman-network.com/sites/default/files/files/suspectListExchange/Surfactant_Suspects_Schymanski_etal_2014.xlsx)  Plus representative structures: [CSV](https://www.norman-network.com/sites/default/files/files/suspectListExchange/230120Update/Surfactant_Suspects_Schymanski_etal_2014_wStruct.csv), [XLSX](https://www.norman-network.com/sites/default/files/files/suspectListExchange/230120Update/Surfactant_Suspects_Schymanski_etal_2014_wStruct.xlsx) (21/11/2020)  CompTox [EAWAGSURF List](https://comptox.epa.gov/dashboard/chemical_lists/eawagsurf) | EAWAGSURF Representative [InChIKeys](https://www.norman-network.com/sites/default/files/files/suspectListExchange/230120Update/EAWAGSURF_InChIKeys_21112019.txt) (21/11/2020) | Schymanski et al. 2014. DOI: [10.1021/es4044374](http://pubs.acs.org/doi/abs/10.1021/es4044374)  DOI: [10.5281/zenodo.2621971](https://doi.org/10.5281/zenodo.2621971) |
| S8 | ATHENSSUS | **University of Athens Surfactants and Suspects List ​** | Gago Ferrero et al [CSV](https://www.norman-network.com/sites/default/files/files/suspectListExchange/031017Update/GagoFerrero_etal_2015_SuspectsNontargets_wDTXSIDs.csv), [XLSX](https://www.norman-network.com/sites/default/files/files/suspectListExchange/031017Update/GagoFerrero_etal_2015_SuspectsNontargets_wDTXSIDs.xlsx) (3/10/2017)  CompTox [ATHENSSUS List](https://comptox.epa.gov/dashboard/chemical_lists/athenssus) | [UniAthens InChIKeys](https://www.norman-network.com/sites/default/files/files/suspectListExchange/UniAthens_SuspectAndSurfactants_InChIKeys.txt) (28/01/2016) | Gago-Ferrero et al. 2015. DOI: [10.1021/acs.est.5b03454](http://pubs.acs.org/doi/abs/10.1021/acs.est.5b03454)  DOI: [10.5281/zenodo.2621979](https://doi.org/10.5281/zenodo.2621979) |
| S9 | PFASTRIER | **PFAS Suspect List: fluorinated substances** | [CSV](https://www.norman-network.com/sites/default/files/files/suspectListExchange/PFAS%20csv%20database%2011.26.15.csv) (MassHunter format; 26/11/2015), [XLSX](https://www.norman-network.com/sites/default/files/files/suspectListExchange/PFAS%20excel%20databse%2011.26.15.xlsx) (several sheets; 26/11/2015)  [Merged CSV](https://www.norman-network.com/sites/default/files/files/suspectListExchange/230120Update/PFASTRIER_26112015.csv) (14/11/2019)  CompTox [PFAS TRIER List](https://comptox.epa.gov/dashboard/chemical_lists/pfastrier) | [PFAS InChIKeys](https://www.norman-network.com/sites/default/files/files/suspectListExchange/PFAS_database_26112015_InChIKeys.txt) (26/11/2015) | Kindly supplied by Xenia Trier, David Lunderberg and colleagues. Reference information contained in files.  DOI: [10.5281/zenodo.2621988](https://doi.org/10.5281/zenodo.2621988) |
| S10 | SWISSPHARMA | **Pharmaceutical List with Consumption Data** | Swiss Pharma [CSV](https://www.norman-network.com/sites/default/files/files/suspectListExchange/031017Update/SwissPharma_TablesS2_CASfix_wDTXSIDs.csv), [XLSX](https://www.norman-network.com/sites/default/files/files/suspectListExchange/031017Update/SwissPharma_TablesS2_CASfix_wDTXSIDs.xlsx) (3/10/2017)  CompTox [SWISSPHARMA List](https://comptox.epa.gov/dashboard/chemical_lists/swisspharma) | [Pharma MS-ready InChIKeys](https://www.norman-network.com/sites/default/files/files/suspectListExchange/SwissPharma_TableS2_InChIKeys.txt) (02/05/2017) | Table S2 from Singer et al. 2016. DOI: [10.1021/acs.est.5b03332](http://pubs.acs.org/doi/abs/10.1021/acs.est.5b03332)  DOI: [10.5281/zenodo.2623484](https://doi.org/10.5281/zenodo.2623484) |
| S11 | SWISSPEST | **Swiss Insecticides, Fungicides and TPs** | Swiss Pesticides [CSV](https://www.norman-network.com/sites/default/files/files/suspectListExchange/031017Update/SwissPesticides_TableS1_CASfix_wDTXSIDs.csv), [XLSX](https://www.norman-network.com/sites/default/files/files/suspectListExchange/031017Update/SwissPesticides_TableS1_CASfix_wDTXSIDs.xlsx) (3/10/2017)  CompTox [SWISSPEST List](https://comptox.epa.gov/dashboard/chemical_lists/swisspest) | [Pesticide MS-ready InChIKeys](https://www.norman-network.com/sites/default/files/files/suspectListExchange/SwissPesticides_TableS1_InChIKeys.txt) (08/05/2017) | Table S1 from  Moschet et al. 2013. DOI: [10.1021/ac4021598](http://pubs.acs.org/doi/abs/10.1021/ac4021598)  DOI: [10.5281/zenodo.2623740](https://doi.org/10.5281/zenodo.2623740) |
| S12 | NORMANEWS | **NormaNEWS for Retrospective Screening of New Emerging Contaminants** | NormaNEWS [CSV](https://www.norman-network.com/sites/default/files/files/suspectListExchange/031017Update/NormaNEWS_V4_26042017_wDTXSIDs.csv), [XLSX](https://www.norman-network.com/sites/default/files/files/suspectListExchange/031017Update/NormaNEWS_V4_26042017_wDTXSIDs.xlsx) (3/10/2017)  CompTox [NORMANEWS List](https://comptox.epa.gov/dashboard/chemical_lists/normanews) | [NormaNEWS InChIKeys](https://www.norman-network.com/sites/default/files/files/suspectListExchange/NormaNEWS_V4_InChIKeys.txt) (8/05/2017) | [NormaNEWS](http://www.norman-network.com/?q=node/244) list provided by Nikiforos Alygizakis, Saer Samanipour and Kevin Thomas. See Alygizakis et al 2018 DOI: [10.1021/acs.est.8b00365](https://pubs.acs.org/doi/abs/10.1021/acs.est.8b00365)  DOI: [10.5281/zenodo.2623815](https://doi.org/10.5281/zenodo.2623815) |
| S13 | EUCOSMETICS | **Combined Inventory of Ingredients Employed in Cosmetic Products (2000) and Revised Inventory (2006)** | Merged Cosmetics [CSV](https://www.norman-network.com/sites/default/files/files/suspectListExchange/Merged_CosmeticProducts_04052017.csv) (4/05/2017)  CompTox [EU Cosmetics List](https://comptox.epa.gov/dashboard/chemical_lists/eucosmetics) | [Merged Cosmetics InChIKeys](https://www.norman-network.com/sites/default/files/files/suspectListExchange/Merged_CosmeticProducts_04052017_InChIKeys.txt) (4/05/2017) | The scientific committee on cosmetic products and non-food products Intended for consumers - [SCCNFP/0389/00 Final](https://www.norman-network.com/sites/default/files/files/suspectListExchange/SCCNFP038900_INCI-2000.pdf) and Commission [Decision 2006/257/EC](https://www.norman-network.com/sites/default/files/files/suspectListExchange/Decision_2006_257_EC.pdf) amending the Decision 96/335/EC. Provided by Peter von der Ohe, UBA, curated by Reza Aalizadeh, University of Athens.  DOI: [10.5281/zenodo.2624118](https://doi.org/10.5281/zenodo.2624118) |
| S14 | KEMIPFAS | **PFAS Highly Fluorinated Substances List: KEMI** | KEMI PFAS Full List [XLSX](https://www.norman-network.com/sites/default/files/files/suspectListExchange/PFAS_Market_KemI_EPA_1Feb2017.xlsx), [CSV](https://www.norman-network.com/sites/default/files/files/suspectListExchange/230120Update/PFAS_Market_KemI_EPA_1Feb2017.csv) (1/02/2017)  KEMI PFAS Structures [XLSX](https://www.norman-network.com/sites/default/files/files/suspectListExchange/PFAS_KEMI_StructOnly_25072017.xlsx), [CSV](https://www.norman-network.com/sites/default/files/files/suspectListExchange/230120Update/PFAS_KEMI_StructOnly_25072017.csv) (3/07/2017)  CompTox [KEMI PFAS List](https://comptox.epa.gov/dashboard/chemical_lists/pfaskemi) | [KEMI PFAS InChIKeys](https://www.norman-network.com/sites/default/files/files/suspectListExchange/PFAS_KEMI_25072017_InChIKeys.txt) (25/07/2017) | Appendix 2 from Swedish Chemicals Agency [KEMI Report 7/15](https://www.norman-network.com/sites/default/files/files/suspectListExchange/report-7-15-occurrence-and-use-of-highly-fluorinated-substances-and-alternatives.pdf). Provided by Stellan Fischer, KEMI. Registration and mapping to [CompTox Dashboard](https://comptox.epa.gov/dashboard/) by Antony Williams, US EPA.  DOI: [10.5281/zenodo.2621524](https://doi.org/10.5281/zenodo.2621524) |
| S15 | NORMANPRI | **NORMAN Priority List** | NORMAN Priority [CSV](https://www.norman-network.com/sites/default/files/files/suspectListExchange/NORMAN_PriorityList_2016.csv) (13/7/2017)  CompTox [NORMAN Priority List](https://comptox.epa.gov/dashboard/chemical_lists/normanpri) | [NORMAN Priority InChIKeys](https://www.norman-network.com/sites/default/files/files/suspectListExchange/NORMAN_PriorityList_2016_InChIKeys.txt) (16/05/2017) | Priority substances from NORMAN WG-1 (Prioritisation), provided by Valeria Dulio.  DOI: [10.5281/zenodo.2624272](https://doi.org/10.5281/zenodo.2624272) |
| S16 | FRENCHLIST | **French Monitoring List** | French List [CSV](https://www.norman-network.com/sites/default/files/files/suspectListExchange/French_List_08052017.csv) (8/05/2017)  CompTox [French Monitoring List](https://comptox.epa.gov/dashboard/chemical_lists/frenchlist) | [French List Unique InChIKeys](https://www.norman-network.com/sites/default/files/files/suspectListExchange/FrenchList_UniqueInChIKeys_08052017.txt) (8/05/2017) | Provided by Valeria Dulio, curated by Reza Aalizadeh, University of Athens.  DOI: [10.5281/zenodo.2624324](https://doi.org/10.5281/zenodo.2624324) |
| S17 | KEMIMARKET | **KEMI Market List** | KEMI Market List [MS-ready](https://www.norman-network.com/sites/default/files/files/suspectListExchange/KEMI_MarketList_12052017_MSready.xlsx) (12/05/2017)  KEMI Market List XLSX [with](https://www.norman-network.com/sites/default/files/files/suspectListExchange/210119Update/KEMI_MarketList_29122017_wCompToxInfo.xlsx) (12 MB) or [without](https://www.norman-network.com/sites/default/files/files/suspectListExchange/210119Update/KEMI_MarketList_29122017.xlsx) (1MB) structures (29/12/2017), as [CSV](https://www.norman-network.com/sites/default/files/files/suspectListExchange/030320Update/KEMI_MarketList_29122017_fixedIKs.csv) (06/02/2020)  CompTox [KEMI Market List](https://comptox.epa.gov/dashboard/chemical_lists/kemimarket) | [KEMI Market MS-ready InChIKeys](https://www.norman-network.com/sites/default/files/files/suspectListExchange/KEMI_MarketList_12052017_MSready_InChIKeys.txt) (12/05/2017)  KEMI Market Full [InChIKeys](https://www.norman-network.com/sites/default/files/files/suspectListExchange/230120Update/KEMIMARKET_29122017_InChIKeys_21112019.txt) (29/12/2017, updated 21/11/2019) | Provided by Stellan Fischer, KEMI including Hazard and Exposure scores, documented [here](https://www.norman-network.com/sites/default/files/files/suspectListExchange/170320Update/MarketList_Documentation_16March2020.docx). Curated by Reza Aalizadeh, University of Athens.  DOI: [10.5281/zenodo.2628786](https://doi.org/10.5281/zenodo.2628786) |
| S18 | TSCASURF | **TSCA Surfactants** | Surfactants [XLSX](https://www.norman-network.com/sites/default/files/files/suspectListExchange/surfactants2.xlsx), [CSV](https://www.norman-network.com/sites/default/files/files/suspectListExchange/230120Update/surfactants2_TSCA_PEG.csv) (25/07/2017)  TSCA PEG [XLSX](https://www.norman-network.com/sites/default/files/files/suspectListExchange/tsca_peg_questionable.xls), [CSV](https://www.norman-network.com/sites/default/files/files/suspectListExchange/230120Update/tsca_peg_questionable.csv) (25/07/2017)  CompTox [TSCA Surfactant List](https://comptox.epa.gov/dashboard/chemical_lists/tscasurf) (subset) | Not applicable | Surfactant information compiled from TSCA by James Little while at Eastman Chemical. More information [here](https://littlemsandsailing.wordpress.com/2011/05/01/identification-of-surfactants-in-commercial-products-by-mass-spectrometry/).  DOI: [10.5281/zenodo.2628791](https://doi.org/10.5281/zenodo.2628791) |
| S19 | MZCLOUD | **mzCloud Compounds** | mzCloud Compounds [XLSX](https://www.norman-network.com/sites/default/files/files/suspectListExchange/080419Update/mzCloud_Compounds_01042019.xlsx), [CSV](https://www.norman-network.com/sites/default/files/files/suspectListExchange/080419Update/mzCloud_Compounds_01042019.csv) (01/04/2019)  CompTox [mzCloud List](https://comptox.epa.gov/dashboard/chemical_lists/mzcloud) | [mzCloud InChIKeys](https://www.norman-network.com/sites/default/files/files/suspectListExchange/080419Update/mzCloud_InChIKeys_01042019.txt) (01/04/2019) | List of compounds on the [mzCloud](https://www.mzcloud.org/) mass spectral database, provided by Robert Mistrik, update by Nikiforos Alygizakis.  DOI: [10.5281/zenodo.2628860](https://doi.org/10.5281/zenodo.2628860) |
| S20 | BISPHENOLS | **Bisphenols** | Bisphenols [CSV](https://www.norman-network.com/sites/default/files/files/suspectListExchange/280520Update/S20_BISPHENOLS_KEMI_NILU_01052020.csv), [XLS](https://www.norman-network.com/sites/default/files/files/suspectListExchange/280520Update/S20_BISPHENOLS_KEMI_NILU_01052020.xlsx) (01/05/2020)  CompTox [Bisphenols List](https://comptox.epa.gov/dashboard/chemical_lists/bisphenols) | [Bisphenol InChIKeys](https://www.norman-network.com/sites/default/files/files/suspectListExchange/280520Update/S20_BISPHENOLS_InChIKeys_01052020.txt) (01/05/2020) | Bisphenols available at [NILU](http://www.nilu.no/) (Pawel Rostkowski) and from Table 3 of report 5/17 by [KEMI](https://www.kemi.se/en/publications/reports/2017/rapport-5-17-bisfenoler---en-kartlaggning-och-analys)(Swedish with English summary). Extended to include exposure scores Jan. 2019 and source information May 2020.  DOI: [10.5281/zenodo.2631744](https://doi.org/10.5281/zenodo.2631744) |
| S21 | UATHTARGETS | **University of Athens Target List** | Target List [CSV](https://www.norman-network.com/sites/default/files/files/suspectListExchange/010322Update/NKUA_LC_target_list_Dec2021.csv), [XLSX](https://www.norman-network.com/sites/default/files/files/suspectListExchange/010322Update/NKUA_LC_target_list_Dec2021.xlsx) (8/2/2022)  CompTox [UATHTARGETS List](https://comptox.epa.gov/dashboard/chemical_lists/UATHTARGETS) | [UoA Targets InChIKeys](https://www.norman-network.com/sites/default/files/files/suspectListExchange/010322Update/NKUA_LC_target_list_Dec2021_InChIKeys.txt) (8/02/2022) | List of target substances from University of Athens, provided by Nikiforos Alygizakis. For more see Alygizakis et al. 2019, DOI: [10.1016/j.envint.2019.03.060](https://doi.org/10.1016/j.envint.2019.03.060),  DOI: [10.5281/zenodo.2632410](https://doi.org/10.5281/zenodo.2632410) |
| S22 | EPACONS | **US EPA Consumer Product Suspect List** | [Original File XLSX](https://www.norman-network.com/sites/default/files/files/suspectListExchange/010318Update/es7b04781_si_002.xlsx) (1/03/2018) [Merged Suspects XLSX](https://www.norman-network.com/sites/default/files/files/suspectListExchange/010318Update/EPA_ConsumerProductSuspects_01032018.xlsx) (1/03/2018) [Merged Suspects CSV](https://www.norman-network.com/sites/default/files/files/suspectListExchange/010318Update/EPA_ConsumerProductSuspects_01032018.csv) (1/03/2018)  CompTox [EPACONS List](https://comptox.epa.gov/dashboard/chemical_lists/epacons) | [Merged Consumer Product InChIKeys](https://www.norman-network.com/sites/default/files/files/suspectListExchange/010318Update/EPA_ConsumerProductSuspects_InChIKeys_01032018.txt) (1/03/2018) | Suspects in supporting information from Phillips et al. 2018, DOI: [10.1021/acs.est.7b04781](https://pubs.acs.org/doi/abs/10.1021/acs.est.7b04781) - Suspect Screening Analysis of Chemicals in Consumer Products with GCxGC-TOF/MS matched with NIST.  DOI: [10.5281/zenodo.2648758](https://doi.org/10.5281/zenodo.2648758) |
| S23 | EIUBASURF | **Surfactant Suspect List from EI and UBA** | Surfactant List [XLSX](https://www.norman-network.com/sites/default/files/files/suspectListExchange/190618Update/SurfactantSuspects_EI_UBA_15032018_wDTXSIDs.xlsx) (19/06/2018) Surfactant List [CSV](https://www.norman-network.com/sites/default/files/files/suspectListExchange/190618Update/SurfactantSuspects_EI_UBA_15032018_wDTXSIDs.csv) (19/06/2018) CompTox [EIUBASURF List](https://comptox.epa.gov/dashboard/chemical_lists/eiubasurf) | EI UBA Surfactant [InChIKeys](https://www.norman-network.com/sites/default/files/files/suspectListExchange/190618Update/SurfactantSuspects_EI_UBA_15032018_InChIKeys.txt) (19/06/2018) | A compiled list of eco-labeled surfactants from Environmental Institute (EI, SK) and the German Federal Environmental Agency (UBA, DE) assigning chemical structures to UVCB chemicals based on names and prior knowledge. Provided by Nikiforos Alygizakis, EI.  DOI: [10.5281/zenodo.2648764](https://doi.org/10.5281/zenodo.2648764) |
| S24 | HUMANNEUROTOX | **List of Human Neurotoxins** | Human Neurotoxin List [XLS](https://www.norman-network.com/sites/default/files/files/suspectListExchange/190618Update/HUMANNEUROTOX-2018-06-19-15-34-35.xls), [CSV](https://www.norman-network.com/sites/default/files/files/suspectListExchange/230120Update/HUMANNEUROTOX-2018-06-19-15-34-35.csv) (19/06/2018) CompTox [HUMANNEUROTOX List](https://comptox.epa.gov/dashboard/chemical_lists/humanneurotox) | Human Neurotox [InChIKeys](https://www.norman-network.com/sites/default/files/files/suspectListExchange/190618Update/HUMANNEUROTOX-2018-06-19_InChIKeys.txt) (19/06/2018) | A set of chemicals listed as neurotoxicants by Grandjean and Landrigan, DOI: [10.1016/S0140-6736(06)69665-7](https://www.thelancet.com/journals/lancet/article/PIIS0140-6736(06)69665-7/fulltext). List provided by Emma Schymanski/Antony Williams.  DOI: [10.5281/zenodo.2648768](https://doi.org/10.5281/zenodo.2648768) |
| S25 | OECDPFAS | **List of PFAS from the OECD** | OECD PFAS [Original List](https://www.norman-network.com/sites/default/files/files/suspectListExchange/190618Update/global-database-of-per-and-polyfluoroalkyl-substances.xlsx) (20/06/2018) OECD PFAS CompTox [XLSX](https://www.norman-network.com/sites/default/files/files/suspectListExchange/210119Update/OECDPFAS_list_22012019.xlsx), [CSV](https://www.norman-network.com/sites/default/files/files/suspectListExchange/030320Update/OECDPFAS_list_22012019.csv) (22/01/2019)  OECD PFAS Life Apex [XLSX](https://www.norman-network.com/sites/default/files/files/suspectListExchange/030519Update/Perfluoroalkyl%20substances_curated%20OECDPFAS_wDTXSIDs.xlsx) (06/05/2019)  CompTox [PFASOECD List](https://comptox.epa.gov/dashboard/chemical_lists/pfasoecd)  CompTox [PFASOECDNA List](https://comptox.epa.gov/dashboard/chemical_lists/pfasoecdna) | OECD PFAS CompTox [InChIKeys](https://www.norman-network.com/sites/default/files/files/suspectListExchange/210119Update/OECDPFAS_InChIKeys_22012019.txt) (22/01/2019)  OECDPFASNA [InChIKeys](https://www.norman-network.com/sites/default/files/files/suspectListExchange/030519Update/OECDPFASNA_InChIKeys_06052019.txt) (06/05/2019) | A list of PFAS released by the OECD, provided by Zhanyun Wang. Details in this [OECD Monograph](https://www.norman-network.com/sites/default/files/files/suspectListExchange/190618Update/ENV-JM-MONO%282018%297.pdf). Extensive registration and curation performed by CompTox Dashboard team (see CompTox files). Curated version for Life Apex provided by Nikiforos Alygizakis (EI, UoA). DOI: [10.5281/zenodo.2648775](https://doi.org/10.5281/zenodo.2648775) |
| S26 | MYCOTOXINS | **List of Mycotoxins from AAFC** | Mycotoxins [CSV](https://www.norman-network.com/sites/default/files/files/suspectListExchange/210119Update/AAFC_Mycotoxins_Jan2019.csv), [XLSX](https://www.norman-network.com/sites/default/files/files/suspectListExchange/210119Update/AAFC_Mycotoxins_Jan2019.xlsx) (23/01/2019)  CompTox [MYCOTOXINS List](http://comptox.epa.gov/dashboard/chemical_lists/mycotoxins) | Mycotoxin [InChIKeys](https://www.norman-network.com/sites/default/files/files/suspectListExchange/190618Update/AAFC_Mycotoxins_Mar2018_InChIKeys.txt) (21/06/2018) | A list of mycotoxins and fungal secondary metabolites provided by Justin Renaud and Mark Sumarah (Agriculture and Agri-Food Canada / Government of Canada). DOI: [10.5281/zenodo.2648815](https://doi.org/10.5281/zenodo.2648815) |
| S27 | KWRSJERPS2 | **Extended Suspect List from Sjerps​ et al​ (KWRSJERPS)** | Full Suspect List [Original File](https://www.norman-network.com/sites/default/files/files/suspectListExchange/190618Update/Sjerp_2016_WatResManuscript_SI.docx), [CSV](https://www.norman-network.com/sites/default/files/files/suspectListExchange/230120Update/KWRSJERPS2_WatResSI.csv) (21/06/2018) CompTox [KWRSJERPS2 List](https://comptox.epa.gov/dashboard/chemical_lists/kwrsjerps2) | KWRSJERPS2 [InChIKeys](https://www.norman-network.com/sites/default/files/files/suspectListExchange/030519Update/KWRSJERPS2_InChIKeys_06052019.txt) (06/05/2019) | Table S4 from Sjerps et al. 2016 Water Research 93: 254-264.  DOI: [10.1016/j.watres.2016.02.034](http://www.sciencedirect.com/science/article/pii/S0043135416300938)  DOI: [10.5281/zenodo.2648817](https://doi.org/10.5281/zenodo.2648817) |
| S28 | EUBIOCIDES | **Biocides from the NORMAN Priority List** | EU Biocide List and data [XLSX](https://www.norman-network.com/sites/default/files/files/suspectListExchange/300718Update/EUBIOCIDES_30072018.xlsx), [CSV](https://www.norman-network.com/sites/default/files/files/suspectListExchange/230120Update/EUBIOCIDES_30072018.csv) (30/07/2018) CompTox [EUBIOCIDES List](https://comptox.epa.gov/dashboard/chemical_lists/eubiocides) | All EU Biocide (salts and MS-ready) [InChIKeys](https://www.norman-network.com/sites/default/files/files/suspectListExchange/300718Update/EUBIOCIDES_all_InChIKeys_30072018.txt) (30/07/2018) | Compounds currently used in the EU as biocides (and partly also as PPP or industrial chemicals) or compounds recently banned as biocides from the 2015 NORMAN priority list, which have been prioritized and assessed for exposure by NORMAN using data from ECHA and other sources. Provided by H. Rüdel, Fraunhofer IME. DOI: [10.5281/zenodo.2648819](https://doi.org/10.5281/zenodo.2648819) |
| S29 | PHYTOTOXINS | **Toxic Plant Phytotoxin (TPPT) Database** | TPPT [Original File](https://www.norman-network.com/sites/default/files/files/suspectListExchange/210119Update/TPPT_database.xlsx) (27/06/2018) TPPT Phytotoxins [CSV](https://www.norman-network.com/sites/default/files/files/suspectListExchange/230120Update/TPPT_Phytotoxins_wDTXSIDs_20112019.csv), [XLSX](https://www.norman-network.com/sites/default/files/files/suspectListExchange/230120Update/TPPT_Phytotoxins_wDTXSIDs_20112019.xlsx) (20/11/2019)  CompTox [PHYTOTOXINS List](https://comptox.epa.gov/dashboard/chemical_lists/phytotoxins) | TPPT [InChIKeys](https://www.norman-network.com/sites/default/files/files/suspectListExchange/230120Update/TPPT_Phytotoxin_InChIKeys_20112019.txt) (20/11/2019) | A comprehensive toxic plant-phytotoxin (TPPT) database provided by Günthardt et al 2018, DOI: [10.1021/acs.jafc.8b01639](https://pubs.acs.org/doi/10.1021/acs.jafc.8b01639).  More information on the [Agroscope TPPT website](https://www.agroscope.admin.ch/agroscope/en/home/publications/apps/tppt.html).  DOI: [10.5281/zenodo.2652993](https://doi.org/10.5281/zenodo.2652993) |
| S30 | PHENANTIOX | **A list of Phenolic Antioxidants from KEMI and NILU** | Phenantiox [CSV](https://www.norman-network.com/sites/default/files/files/suspectListExchange/210119Update/PhenolicAntioxidants_KEMI_NILU_Jan2019.csv), [XLSX](https://www.norman-network.com/sites/default/files/files/suspectListExchange/210119Update/PhenolicAntioxidants_KEMI_NILU_Jan2019.xlsx) (23/01/2019)  CompTox [PHENANTIOX List](https://comptox.epa.gov/dashboard/chemical_lists/phenantiox) | Phenantiox [InChIKeys](https://www.norman-network.com/sites/default/files/files/suspectListExchange/210119Update/PhenolicAntioxidants_Jan2019_InChIKeys.txt) (23/01/2019) | A list of possible phenolic antioxidants with exposure scores compiled by Stellan Fischer (KEMI) and Pawel Rostkowski (NILU). Mapped to CompTox information using CAS numbers.  DOI: [10.5281/zenodo.2653011](https://doi.org/10.5281/zenodo.2653011) |
| S31 | WRTMSD | **Wiley Registry of Tandem Mass Spectral Data, MSforID** | WRTMSD [CSV](https://www.norman-network.com/sites/default/files/files/suspectListExchange/210119Update/WRTMSD_wDTXSIDs_24012019.csv), [XLSX](https://www.norman-network.com/sites/default/files/files/suspectListExchange/210119Update/WRTMSD_wDTXSIDs_24012019.xlsx) (24/01/2019)  CompTox [WRTMSD List](https://comptox.epa.gov/dashboard/chemical_lists/wrtmsd) | WRTMSD [InChIKeys](https://www.norman-network.com/sites/default/files/files/suspectListExchange/210119Update/WRTMSD_InChIKeys.txt) (24/01/2019) | The "Wiley Registry of Tandem Mass Spectral Data, MSforID" contains high-quality tandem MSacquired on a QqTOF instrument, developed by Herbert Oberacher (Medical University of Innsbruck, Austria). More information at [www.msforid.com](http://www.msforid.com/)  DOI: [10.5281/zenodo.2653016](https://doi.org/10.5281/zenodo.2653016) |
| S32 | REACH2017 | **>68,600 REACH Chemicals** | REACH2017 [XLSX](https://www.norman-network.com/sites/default/files/files/suspectListExchange/061120Update/REACH2017.xlsx), [CSV](https://www.norman-network.com/sites/default/files/files/suspectListExchange/061120Update/REACH2017_fixedSMILES.csv) (06/11/2020)  CompTox [REACH2017 List](https://comptox.epa.gov/dashboard/chemical_lists/reach2017) | REACH2017 [InChIKeys](https://www.norman-network.com/sites/default/files/files/suspectListExchange/061120Update/REACH2017_InChIKeys.txt) (06/11/2020) | A list of >68,600 REACH chemicals including InChIKeys and spectral information, provided by N. Alygizakis and J. Slobodnik, EI.  DOI: [10.5281/zenodo.2653020](https://doi.org/10.5281/zenodo.2653020) |
| S33 | SOLUTIONSMLOS | **Chemicals used for Modelling in SOLUTIONS** | SOLUTIONSMLOS [XLSX](https://www.norman-network.com/sites/default/files/files/suspectListExchange/061120Update/SOLUTIONSMLOS.xlsx), [CSV](https://www.norman-network.com/sites/default/files/files/suspectListExchange/061120Update/SOLUTIONSMLOS.csv) (06/11/2020)  CompTox [SOLUTIONSMLOS List](https://comptox.epa.gov/dashboard/chemical_lists/solutionsmlos) | SOLUTIONSMLOS [InChIKeys](https://www.norman-network.com/sites/default/files/files/suspectListExchange/061120Update/SOLUTIONSMLOS_InChIKeys.txt) (06/11/2020) | SOLUTIONSMLOS contains the 6462 chemicals used for modelling in the SOLUTIONS project ([www.solutions-project.eu/](http://www.solutions-project.eu/)), provided by Jaroslav Slobodnik (EI).  DOI: [10.5281/zenodo.2653022](https://doi.org/10.5281/zenodo.2653022) |
| S34 | EXPOSOMEXPL | **Biomarkers from Exposome Explorer** | Exposome Explorer Biomarkers Download [XLSX](https://www.norman-network.com/sites/default/files/files/suspectListExchange/210119Update/ExposomeExplorer-biomarkers.xlsx) (24/01/2019) EXPOSOMEXPL Mapped [CSV](https://www.norman-network.com/sites/default/files/files/suspectListExchange/210119Update/EXPOSOMEXPL_wDTXSIDs_24012019.csv), [XLSX](https://www.norman-network.com/sites/default/files/files/suspectListExchange/210119Update/EXPOSOMEXPL_wDTXSIDs_24012019.xlsx) (24/01/2019) CompTox [EXPOSOMEXPL List](https://comptox.epa.gov/dashboard/chemical_lists/exposomexpl) | EXPOSOMEXPL [InChIKeys](https://www.norman-network.com/sites/default/files/files/suspectListExchange/210119Update/EXPOSOMEXPL_InChIKeys_24012019.txt) (24/01/2019) | The Exposome-Explorer (<http://exposome-explorer.iarc.fr/>) is dedicated to biomarkers of exposure to environmental risk factors for diseases (Neveu et al 2017, DOI: [10.1093/nar/gkw980](http://doi.org/10.1093/nar/gkw980)). Provided by Reza Salek and Vanessa Neveu (IARC), mapping files to all discrete chemicals by A. Williams/E. Schymanski. DOI: [10.5281/zenodo.2653031](https://doi.org/10.5281/zenodo.2653031) |
| S35 | INDOORCT16 | **Indoor Environment Substances from 2016 Collaborative Trial** | Indoor CT Lists [XLSX](https://www.norman-network.com/sites/default/files/files/suspectListExchange/120219Update/Merged%20list%20of%20compounds_CT_indoor_dust_14022019.xlsx) (14/02/2019)  INDOORCT16 Merged [CSV](https://www.norman-network.com/sites/default/files/files/suspectListExchange/030320Update/INDOORCT16_merged.csv), [XLSX](https://www.norman-network.com/sites/default/files/files/suspectListExchange/120219Update/INDOORCT16_merged_14022019.xlsx) (14/02/2019)  CompTox [INDOORCT16 List](https://comptox.epa.gov/dashboard/chemical_lists/indoorct16) | INDOORCT16 Merged [InChIKeys](https://www.norman-network.com/sites/default/files/files/suspectListExchange/120219Update/INDOORCT16_merged_InChIKeys_14022019.txt) (14/02/2019) | Lists of GC-MS and LC-MS compounds and DSFP output, plus merged files from the Indoor Dust Collaborative Trial, 2016 provided by Peter Haglund (UMU) and Pawel Rostkowski (NILU). Details in Rostkowski et al. 2019 DOI: [10.1007/s00216-019-01615-6](https://link.springer.com/article/10.1007/s00216-019-01615-6). DOI: [10.5281/zenodo.2653206](https://doi.org/10.5281/zenodo.2653206) |
| S36 | UBAPMT | **Potential Persistent, Mobile and Toxic (PMT) substances** | UBAPMT as [CSV](https://www.norman-network.com/sites/default/files/files/suspectListExchange/250422Update/S36_UBAPMT_April2022.csv), [XLSX](https://www.norman-network.com/sites/default/files/files/suspectListExchange/250422Update/S36_UBAPMT_April2022.xlsx) (25/04/2022)  CompTox [UBAPMT List](https://comptox.epa.gov/dashboard/chemical_lists/ubapmt) | UBAPMT [InChIKeys](https://www.norman-network.com/sites/default/files/files/suspectListExchange/250422Update/S36_UBAPMT_April2022_InChIKeys.txt) (25/04/2022) | A list of REACH substances that could fulfil proposed (very) Persistent, (very) Mobile and Toxic (PMT/vPvM) criteria according to [UBA](https://www.umweltbundesamt.de/en), Germany and also currently proposed by the European Commission (EC) for the [CLP](https://echa.europa.eu/guidance-documents/guidance-on-clp) criteria. Derived from a research project by Norwegian Geotechnical Institute (NGI). Updated version provided by Hans Peter Arp, details in [UBA Report 126/2019](https://www.umweltbundesamt.de/publikationen/reach-improvement-of-guidance-methods-for-the) and in an upcoming 2022 revised version. Funding was provided by the Federal Ministry for the Environment, Nature Conservation, Building and Nuclear Safety of Germany (FKZ3719654080). DOI: [10.5281/zenodo.2653212](https://doi.org/10.5281/zenodo.2653212) |
| S37 | LITMINEDNEURO | **Neurotoxicants from literature mining PubMed** | LITMINEDNEURO [CSV](https://www.norman-network.com/sites/default/files/files/suspectListExchange/210119Update/LITMINEDNEURO_25012019.csv), [XLSX](https://www.norman-network.com/sites/default/files/files/suspectListExchange/210119Update/LITMINEDNEURO_25012019.xlsx) (25/01/2019)  CompTox [LITMINEDNEURO List](https://comptox.epa.gov/dashboard/chemical_lists/LITMINEDNEURO) | LITMINEDNEURO [InChIKeys](https://www.norman-network.com/sites/default/files/files/suspectListExchange/210119Update/LITMINEDNEURO_InChIKeys.txt) (25/01/2019) | A list of chemicals associated with neurotoxicity compiled through systematic literature mining of PubMed using MeSH terms, compiled by Nancy Baker, Antony Williams (US EPA) and Emma Schymanski (LCSB), details in Schymanski et al (2019) DOI: [10.1039/C9EM00068B](https://doi.org/10.1039/C9EM00068B). DOI: [10.5281/zenodo.2653214](https://doi.org/10.5281/zenodo.2653214) |
| S38 | SOLNSLMCTPS | **SOLUTIONS Predicted Transformation Products by LMC** | Original File [XLSX](https://www.norman-network.com/sites/default/files/files/suspectListExchange/120219Update/modelsTransformationProducts.xlsx), [CSV](https://www.norman-network.com/sites/default/files/files/suspectListExchange/230120Update/modelsTransformationProducts.csv) (14/02/2019)  CompTox [SOLNSLMCTPS List](https://comptox.epa.gov/dashboard/chemical_lists/solnslmctps) | SOLNSLMCTPS [InChIKeys](https://www.norman-network.com/sites/default/files/files/suspectListExchange/120219Update/SOLNSLMCTPS_InChIKeys.txt) (14/02/2019) | Predicted Transformation Products calculated by LMC during the SOLUTIONS project, interactive table available [here](https://www.normandata.eu/solutions/modelsTransformationProducts.php).  DOI: [10.5281/zenodo.2653560](https://doi.org/10.5281/zenodo.2653560) |
| S39 | KEMIWWSUS | **Wastewater Suspect List based on Swedish Product Data** | Wastewater Suspect List [XLSX](https://www.norman-network.com/sites/default/files/files/suspectListExchange/230120Update/Suspects_WasteWater_Sweden_KEMI20190212_updated.xlsx), [CSV](https://www.norman-network.com/sites/default/files/files/suspectListExchange/230120Update/Suspects_Wastewater_Sweden_KEMI20190212_wDTXSIDs.csv) (14/11/2019)  CompTox [KEMIWWSUS List](https://comptox.epa.gov/dashboard/chemical_lists/kemiwwsus) | KEMIWWSUS [InChIKeys](https://www.norman-network.com/sites/default/files/files/suspectListExchange/120219Update/KEMIWWSUS_InChIKeys_12022019.txt) (12/02/2019) | A prioritized list of 1,123 substances relevant for wastewater based on Swedish product registry data, including scores. Provided by Stellan Fischer, KEMI.  DOI: [10.5281/zenodo.2653566](https://doi.org/10.5281/zenodo.2653566) |
| S40 | ALGALTOX | **Algal toxins list from CompTox** | ALGALTOX [XLSX](https://www.norman-network.com/sites/default/files/files/suspectListExchange/120219Update/ALGALTOX_14022019.xlsx), [CSV](https://www.norman-network.com/sites/default/files/files/suspectListExchange/120219Update/ALGALTOX_14022019.csv) (14/02/2019) CompTox [ALGALTOX List](https://comptox.epa.gov/dashboard/chemical_lists/algaltox) | ALGALTOX [InChIKeys](https://www.norman-network.com/sites/default/files/files/suspectListExchange/120219Update/ALGALTOX_InChIKeys_14022019.txt) (14/02/2019) | List of algal toxins (generated during blooms) from the CompTox Chemicals Dashboard.  DOI: [10.5281/zenodo.2656710](https://doi.org/10.5281/zenodo.2656710) |
| S41 | CCL4 | **CCL 4 Contaminant Candidate List** | CCL4 [XLSX](https://www.norman-network.com/sites/default/files/files/suspectListExchange/120219Update/CCL4_14022019.xlsx), [CSV](https://www.norman-network.com/sites/default/files/files/suspectListExchange/120219Update/CCL4_14022019.csv) (14/02/2019) CompTox [CCL4 List](https://comptox.epa.gov/dashboard/chemical_lists/ccl4) | CCL4 [InChIKeys](https://www.norman-network.com/sites/default/files/files/suspectListExchange/120219Update/CCL4_InChIKeys_14022019.txt) (14/02/2019) | Contaminants that are not (yet) regulated in the USA but are known or anticipated to occur in public water systems; from CompTox.  DOI: [10.5281/zenodo.2656716](https://doi.org/10.5281/zenodo.2656716) |
| S42 | HDXNOEX | **Hydrogen Deuterium Exchange (HDX) Standard Set** | HDXNOEX [XLSX](https://www.norman-network.com/sites/default/files/files/suspectListExchange/120219Update/HDXNOEX_14022019.xlsx), [CSV](https://www.norman-network.com/sites/default/files/files/suspectListExchange/120219Update/HDXNOEX_14022019.csv) (14/02/2019) CompTox [HDXNOEX List](https://comptox.epa.gov/dashboard/chemical_lists/hdxnoex) CompTox [HDXEXCH List](https://comptox.epa.gov/dashboard/chemical_lists/hdxexch) | HDXNOEX [InChIKeys](https://www.norman-network.com/sites/default/files/files/suspectListExchange/120219Update/HDXNOEX_InChIKeys_14022019.txt) (14/02/2019) | Environmental standard set used to investigate hydrogen deuterium exchange in small molecule HRMS (Ruttkies et al. 2019, DOI: [10.1007/s00216-019-01885-0](https://doi.org/10.1007/s00216-019-01885-0)). [HDXEXCH](https://comptox.epa.gov/dashboard/chemical_lists/hdxexch) list also contains observed deuterated species. DOI: [10.5281/zenodo.2656724](https://doi.org/10.5281/zenodo.2656724) |
| S43 | NEUROTOXINS | **Neurotoxicants Collection from Public Resources** | NEUROTOXINS [XLSX](https://www.norman-network.com/sites/default/files/files/suspectListExchange/120219Update/NEUROTOXINS_14022019.xlsx), [CSV](https://www.norman-network.com/sites/default/files/files/suspectListExchange/120219Update/NEUROTOXINS_14022019.csv) (14/02/2019)  CompTox [NEUROTOXINS List](https://comptox.epa.gov/dashboard/chemical_lists/neurotoxins) | NEUROTOXINS [InChIKeys](https://www.norman-network.com/sites/default/files/files/suspectListExchange/120219Update/NEUROTOXINS_InChIKeys_14022019.txt) (14/02/2019) | A list of neurotoxicants compiled from public resources, details on CompTox and Schymanski et al. (2019) DOI: [10.1039/C9EM00068B](https://doi.org/10.1039/C9EM00068B).  DOI: [10.5281/zenodo.2656729](https://doi.org/10.5281/zenodo.2656729) |
| S44 | STATINS | **Statins Collection from Public Resources** | STATINS [XLSX](https://www.norman-network.com/sites/default/files/files/suspectListExchange/120219Update/STATINS_14022019.xlsx), [CSV](https://www.norman-network.com/sites/default/files/files/suspectListExchange/120219Update/STATINS_14022019.csv) (14/02/2019)  CompTox [STATINS List](https://comptox.epa.gov/dashboard/chemical_lists/statins) | STATINS [InChIKeys](https://www.norman-network.com/sites/default/files/files/suspectListExchange/120219Update/STATINS_InChIKeys_14022019.txt) (14/02/2019) | A list of statins (lipid-lowering medications) compiled from public resources, details on CompTox.  DOI: [10.5281/zenodo.2656736](https://doi.org/10.5281/zenodo.2656736) |
| S45 | SYNTHCANNAB | **Synthetic Cannabinoids** | SYNTHCANNAB [XLSX](https://www.norman-network.com/sites/default/files/files/suspectListExchange/170619Update/SYNTHCANNAB_17062019.xlsx), [CSV](https://www.norman-network.com/sites/default/files/files/suspectListExchange/170619Update/SYNTHCANNAB_17062019.csv) (17/06/2019)  CompTox [SYNTHCANNAB List](https://comptox.epa.gov/dashboard/chemical_lists/synthcannab) | SYNTHCANNAB [InChIKeys](https://www.norman-network.com/sites/default/files/files/suspectListExchange/170619Update/SYNTHCANNAB_InChIKeys_17062019.txt) (17/06/2019) | A list of synthetic cannabinoids compounds assembled from public resources, from CompTox.  DOI: [10.5281/zenodo.2656740](https://doi.org/10.5281/zenodo.2656740) |
| S46 | PFASNTREV19 | **List of PFAS reported in Non-Target HRMS Studies (Liu et al 2019)** | Table 1 from Liu et al as [PDF](https://www.norman-network.com/sites/default/files/files/suspectListExchange/220319Update/Liu_etal_2019_HRMS_PFAS_1-s2.0-S0165993618306253-Table1.pdf) MS-ready form in [XLSX](https://www.norman-network.com/sites/default/files/files/suspectListExchange/080419Update/PFASNTREV19_Liu_etal_17042019.xlsx) (formatted) and [CSV](https://www.norman-network.com/sites/default/files/files/suspectListExchange/030320Update/PFASNTREV19_Liu_etal_17042019.csv) (17/04/2019)  CompTox [PFASNTREV19 List](https://comptox.epa.gov/dashboard/chemical_lists/PFASNTREV19) | PFASNTREV19 [InChIKeys](https://www.norman-network.com/sites/default/files/files/suspectListExchange/080419Update/PFASNTREV19_InChIKeys_17042019.txt) (17/04/2019) | List compiled in the non-target HRMS PFAS review by Liu et al 2019, DOI: [10.1016/j.trac.2019.02.021](https://doi.org/10.1016/j.trac.2019.02.021). MS-ready list prepared by Yanna Liu, Lisa D’Agostino, Emma Schymanski and Jon Martin. Note not all entries have structures.  DOI: [10.5281/zenodo.2656744](https://doi.org/10.5281/zenodo.2656744) |
| S47 | ECHAPLASTICS | **A list from the Plastic Additives Initiative Mapping Exercise by ECHA** | Merged ECHA Plastic Additives with Structures [XLSX](https://www.norman-network.com/sites/default/files/files/suspectListExchange/220319Update/ECHA_PlasticAdditivesInitiative_06032019.xlsx), [CSV](https://www.norman-network.com/sites/default/files/files/suspectListExchange/220319Update/ECHA_PlasticAdditivesInitiative_06032019.csv) (06/03/2019)  CompTox [ECHAPLASTICS List](https://comptox.epa.gov/dashboard/chemical_lists/echaplastics) | ECHA Plastic Additives [InChIKeys](https://www.norman-network.com/sites/default/files/files/suspectListExchange/220319Update/ECHA_Plastics_InChIKeys.txt) (06/03/2019) | List with several categories released on <https://echa.europa.eu/mapping-exercise-plastic-additives-initiative> and mapped to structures by CAS and Name by E. Schymanski.  DOI: [10.5281/zenodo.2658139](https://doi.org/10.5281/zenodo.2658139) |
| S48  S49 | CPPDBLISTA  CPPDBLISTB | **Database of Chemicals associated with Plastic Packaging (CPPdb)** | CPPdb Original File (List A and B) [XLSX](https://www.norman-network.com/sites/default/files/files/suspectListExchange/220319Update/CPPdb_ListA_ListB_181009_ZenodoV1.xlsx) (06/03/2019) Mapped Files (06/03/2019): Table 2 from Groh et al as [XLSX](https://www.norman-network.com/sites/default/files/files/suspectListExchange/220319Update/Table2_Groh_etal_stoten_mapped.xlsx), [CSV](https://www.norman-network.com/sites/default/files/files/suspectListExchange/220319Update/Table2_Groh_etal_stoten_mapped.csv)  CPPdb List A [XLSX](https://www.norman-network.com/sites/default/files/files/suspectListExchange/220319Update/CPPdb_ListA_Mapped_06032019.xlsx), [CSV](https://www.norman-network.com/sites/default/files/files/suspectListExchange/220319Update/CPPdb_ListA_Mapped_06032019.csv)  CPPdb List B [XLSX](https://www.norman-network.com/sites/default/files/files/suspectListExchange/220319Update/CPPdb_ListB_Mapped_06032019.xlsx), [CSV](https://www.norman-network.com/sites/default/files/files/suspectListExchange/220319Update/CPPdb_ListB_Mapped_06032019.csv)  CompTox [CPPDBLISTA List](https://comptox.epa.gov/dashboard/chemical_lists/cppdblista) CompTox [CPPDBLISTB List](https://comptox.epa.gov/dashboard/chemical_lists/cppdblistb) | Table 2 Groh et al. [InChIKeys](https://www.norman-network.com/sites/default/files/files/suspectListExchange/220319Update/Table2_Groh_etal_InChIKeys.txt) CPPdb List A [InChIKeys](https://www.norman-network.com/sites/default/files/files/suspectListExchange/220319Update/CPPdb_ListA_InChIKeys.txt) CPPdb List B [InChIKeys](https://www.norman-network.com/sites/default/files/files/suspectListExchange/220319Update/CPPdb_ListB_InChIKeys.txt) (all 06/03/2019) | A database of chemicals likely (List A, 903) and possibly (List B, 3353) associated with plastic packaging, with hazard data, from Groh et al 2019 DOI: [10.1016/j.scitotenv.2018.10.015](https://doi.org/10.1016/j.scitotenv.2018.10.015). Mapped to structures by CAS/Name by K. Groh &E. Schymanski. Latest version (last update Oct 2018): DOI: [10.5281/zenodo.1287773](http://doi.org/10.5281/zenodo.1287773)  List A DOI: [10.5281/zenodo.2658143](https://doi.org/10.5281/zenodo.2658143) List B DOI: [10.5281/zenodo.2658152](https://doi.org/10.5281/zenodo.2658152) |
| S50 | CCSCOMPEND | **The Unified Collision Cross Section (CCS) Compendium** | CCS as [CSV](https://www.norman-network.com/sites/default/files/files/suspectListExchange/220319Update/20190304JAP_CCSdatabase_final.csv), [XLSX](https://www.norman-network.com/sites/default/files/files/suspectListExchange/220319Update/20190304JAP_CCSdatabase_final.xlsx) (04/03/2019)  CompTox [CCSCOMPEND List](https://comptox.epa.gov/dashboard/chemical_lists/ccscompend) | CCS [InChIKeys](https://www.norman-network.com/sites/default/files/files/suspectListExchange/220319Update/CCSdatabase_InChIKeys_29032019.txt) (29/03/2019) | >3800 experimental collision cross section values (drift tube MS), provided by Jackie Picache and John McLean, Vanderbilt. Further details available here: <https://lab.vanderbilt.edu/mclean-group/collision-cross-section-database/>  DOI: [10.5281/zenodo.2658162](https://doi.org/10.5281/zenodo.2658162) |
| S51 | WRIGCHRMS | **GC-HRMS target list of WRI** | WRIGCHRMS [XLSX](https://www.norman-network.com/sites/default/files/files/suspectListExchange/080419Update/WRIGCHRMS_04042019.xlsx), [CSV](https://www.norman-network.com/sites/default/files/files/suspectListExchange/080419Update/WRIGCHRMS_04042019.csv) (04/04/2019)  CompTox [WRIGCHRMS List](https://comptox.epa.gov/dashboard/chemical_lists/WRIGCHRMS) | WRIGCHRMS [InChIKeys](https://www.norman-network.com/sites/default/files/files/suspectListExchange/080419Update/WRIGCHRMS_InChIKeys_04042019.txt) (04/04/2019) | GC-HRMS target list of WRI. The method was established by Agilent. The list was provided by Michal Kirchner (Slovak Water Research Institute, WRI) and curated by Nikiforos Alygizakis (EI/UoA)  DOI: [10.5281/zenodo.2658169](https://doi.org/10.5281/zenodo.2658169) |
| S52 | THSMOKE | **Thirdhand Smoke (THS) Compounds** | THSMOKE [XLSX](https://www.norman-network.com/sites/default/files/files/suspectListExchange/030519Update/THSMOKE_THS_Suspects_06052019.xlsx), [CSV](https://www.norman-network.com/sites/default/files/files/suspectListExchange/030519Update/THSMOKE_THS_Suspects_06052019.csv) (06/05/2019)  CompTox [THSMOKE List](https://comptox.epa.gov/dashboard/chemical_lists/thsmoke) | THSMOKE [InChIKeys](https://www.norman-network.com/sites/default/files/files/suspectListExchange/030519Update/THS_InChIKeys_06052019.txt) (06/05/2019) | Thirdhand Smoke (THS, the tobacco-related gases and particles that become embedded in materials), suspect list compiled by Sonia Torres and Noelia Ramirez (IISPV-URV) and Emma Schymanski (LCSB). DOI: [10.5281/zenodo.2669466](https://doi.org/10.5281/zenodo.2669466) |
| S53 | UFZWANATARG | **Target Compounds from UFZ WANA** | Target List as [XLSX](https://www.norman-network.com/sites/default/files/files/suspectListExchange/110819Update/UFZWANATARG_11082019.xlsx), [CSV](https://www.norman-network.com/sites/default/files/files/suspectListExchange/110819Update/UFZWANATARG_11082019.csv) (11/08/2019) CompTox [UFZWANATARG List](https://comptox.epa.gov/dashboard/chemical_lists/UFZWANATARG) | UFZWANATARG InChIKeys – [All](https://www.norman-network.com/sites/default/files/files/suspectListExchange/110819Update/UFZWANATARG_InChIKeys_all_11082019.txt), [LC](https://www.norman-network.com/sites/default/files/files/suspectListExchange/110819Update/UFZWANATARG_InChIKeys_LC_11082019.txt), [GC](https://www.norman-network.com/sites/default/files/files/suspectListExchange/110819Update/UFZWANATARG_InChIKeys_GC_11082019.txt) (11/08/2019) | List of target compounds (LC and GC) measured at WANA, UFZ (Leipzig, Germany), provided by Tobias Schulze and Martin Krauss.  DOI: [10.5281/zenodo.3365549](https://doi.org/10.5281/zenodo.3365549) |
| S54 | EFSAPRI | **European Food Safety Authority Priority Substances** | EFSAPRI Full List [XLSX](https://www.norman-network.com/sites/default/files/files/suspectListExchange/170619Update/EFSAPRI_FullList_10062019.xlsx) (10/06/2019)  EFSAPRI 212 List [XLSX](https://www.norman-network.com/sites/default/files/files/suspectListExchange/170619Update/EFSAPRI_212List_18062019.xlsx), [CSV](https://www.norman-network.com/sites/default/files/files/suspectListExchange/170619Update/EFSAPRI_212List_18062019.csv) (18/06/2019)  CompTox [EFSAPRI List](https://comptox.epa.gov/dashboard/chemical_lists/EFSAPRI) | EFSAPRI 212 [InChIKeys](https://www.norman-network.com/sites/default/files/files/suspectListExchange/170619Update/EFSAPRI_212List_InChIKeys_18062019.txt) (18/06/2019) | List of 212 REACH substances prioritized by Oltmanns et al., DOI: [10.2903/sp.efsa.2019.EN-1597](https://doi.org/10.2903/sp.efsa.2019.EN-1597), European Food Safety Authority (EFSA). 2,336 compounds were assessed for (i) environmental release, (ii) biodegradation, (iii) bioaccumulation in food/feed and (iv) toxicity, resulting in 212 priority compounds.  DOI: [10.5281/zenodo.3248993](https://zenodo.org/record/3248994#.XRM7Z-szapo) |
| S55 | ZINC15PHARMA | **>8600 Pharmaceuticals from ZINC15** | Original file with Fragments as [CSV](https://www.norman-network.com/sites/default/files/files/suspectListExchange/061120Update/ZINC15PHARMA.csv), [XLSX](https://www.norman-network.com/sites/default/files/files/suspectListExchange/061120Update/ZINC15PHARMA.xlsx) (06/11/2020)  CompTox [ZINC15PHARMA List](https://comptox.epa.gov/dashboard/chemical_lists/ZINC15PHARMA) | ZINC15PHARMA MS-ready [InChIKeys](https://www.norman-network.com/sites/default/files/files/suspectListExchange/061120Update/ZINC15PHARMA_MSready_InChIKeys.txt) (06/11/2020) | A list of >8600 pharmaceuticals retrieved from [ZINC15](http://zinc15.docking.org/substances/subsets/world-not-fda/), curated and provided by Reza Aalizadeh, University of Athens.  DOI: [10.5281/zenodo.3247749](https://doi.org/10.5281/zenodo.3247749) |
| S56 | UOATARGPHARMA | **Target Pharmaceutical/Drug List from University of Athens** | Target List [CSV](https://www.norman-network.com/sites/default/files/files/suspectListExchange/170619Update/UOATARGPHARMA_18062019.csv), [XLSX](https://www.norman-network.com/sites/default/files/files/suspectListExchange/170619Update/UOATARGPHARMA_18062019.xlsx) (18/06/2019)  CompTox [UOATARGPHARMA List](https://comptox.epa.gov/dashboard/chemical_lists/UOATARGPHARMA) | UOATARGPHARMA [InChIKeys](https://www.norman-network.com/sites/default/files/files/suspectListExchange/170619Update/UOATARGPHARMA_InChIKeys_18062019.txt) (18/06/2019) | LC-MS/MS target list of University of Athens containing illicit drugs and pharmaceuticals. Provided by Nikiforos Alygizakis (EI/UoA) and Nikolaos Thomaidis (UoA).  DOI: [10.1021/acs.est.6b02417](https://pubs.acs.org/doi/10.1021/acs.est.6b02417), [10.1016/j.scitotenv.2015.09.145](https://doi.org/10.1016/j.scitotenv.2015.09.145), [10.5281/zenodo.3248837](https://doi.org/10.5281/zenodo.3248837) |
| S57 | GREEKPHARMA | **Suspect Pharmaceuticals from the National Organization of Medicine, Greece** | Suspect List [CSV](https://www.norman-network.com/sites/default/files/files/suspectListExchange/170619Update/GREEKPHARMA_18062019.csv), [XLSX](https://www.norman-network.com/sites/default/files/files/suspectListExchange/170619Update/GREEKPHARMA_18062019.xlsx) (18/06/2019)  CompTox [GREEKPHARMA List](https://comptox.epa.gov/dashboard/chemical_lists/greekpharma) | GREEKPHARMA [InChIKeys](https://www.norman-network.com/sites/default/files/files/suspectListExchange/170619Update/GREEKPHARMA_InChIKeys_18062019.txt) (18/06/2019) | A pharmaceutical suspect list containing antibiotics, antidepressants, antipsychotics, benzodiazepines, NSAIDs, antilipidemic drugs, antiepileptic drugs, antiulcer drugs, antihypertensive and diuretic drugs, extracted from the National Organization for Medicines of Greece. This is complementary to UOATARGPHARMA. Provided by Katerina Galani (UoA), Nikiforos Alygizakis (EI/UoA) and Nikolaos Thomaidis (UoA). DOI: [10.5281/zenodo.3248883](https://doi.org/10.5281/zenodo.3248883) |
| S58 | PSYCHOCANNAB | **Synthetic Cannabinoids and Psychoactive Compounds** | PSYCHOCANNAB [XLSX](https://www.norman-network.com/sites/default/files/files/suspectListExchange/170619Update/PSYCHOCANNAB_17062019.xlsx), [CSV](https://www.norman-network.com/sites/default/files/files/suspectListExchange/170619Update/PSYCHOCANNAB_17062019.csv) (17/06/2019)  CompTox [PSCHYOCANNAB List](https://comptox.epa.gov/dashboard/chemical_lists/PSYCHOCANNAB) | PSYCHOCANNAB [InChIKeys](https://www.norman-network.com/sites/default/files/files/suspectListExchange/170619Update/PSYCHOCANNAB_InChIKeys_17062019.txt) (17/06/2019) | A list of synthetic cannabinoids and psychoactive compounds assembled from public resources, from CompTox Chemicals Dashboard.  DOI: [10.5281/zenodo.3247723](https://doi.org/10.5281/zenodo.3247723) |
| S59 | NPINESCT | **Natural Product Insecticides** | NPINSECT [XLSX](https://www.norman-network.com/sites/default/files/files/suspectListExchange/230120Update/S59_Natural_Products_Insecticides_v2.xlsx), [CSV](https://www.norman-network.com/sites/default/files/files/suspectListExchange/230120Update/S59_Natural_Products_Insecticides_v2.csv) (20/11/2019)  CompTox [NPINSECT List](https://comptox.epa.gov/dashboard/chemical_lists/NPINSECT) | NPINSECT [InChIKeys](https://www.norman-network.com/sites/default/files/files/suspectListExchange/230120Update/S59_NPI_InChIKeys.txt) (20/11/2019) | A list of 83 naturally occurring insecticides curated and provided by Reza Aalizadeh (University of Athens).  DOI: [10.5281/zenodo.3544741](https://doi.org/10.5281/zenodo.3544741) |
| S60 | SWISSPEST19 | **Swiss Pesticides and Metabolites from Kiefer et al 2019** | Original file as [XLSX](https://www.norman-network.com/sites/default/files/files/suspectListExchange/230120Update/SI_B_Kiefer_et_al_2019.xlsx) (17/11/2019)  SWISSPEST19 as [XLSX](https://www.norman-network.com/sites/default/files/files/suspectListExchange/280520Update/S60_SWISSPEST19_25042020.xlsx), [CSV](https://www.norman-network.com/sites/default/files/files/suspectListExchange/280520Update/S60_SWISSPEST19_25042020.csv) (25/04/2020)  CompTox [SWISSPEST19 List](https://comptox.epa.gov/dashboard/chemical_lists/SWISSPEST19) | SWISSPEST19 [InChIKeys](https://www.norman-network.com/sites/default/files/files/suspectListExchange/230120Update/S60_SWISSPEST19_InChIKeys.txt) (17/11/2019) | Swiss pesticides (plant protection products) and metabolites from Kiefer et al 2019 (Eawag), Tables SI-B 1 and 2. DOI: [10.1016/j.watres.2019.114972](https://doi.org/10.1016/j.watres.2019.114972), [10.5281/zenodo.3544759](https://doi.org/10.5281/zenodo.3544759) |
| S61 | UJICCSLIB | **Collision Cross Section (CCS) Library from UJI** | UJI CCS Library as [XLSX](https://www.norman-network.com/sites/default/files/files/suspectListExchange/300720Update/TableS1_CCS_RT_mz_DB_IUPA_charge.xlsx), [CSV](https://www.norman-network.com/sites/default/files/files/suspectListExchange/300720Update/TableS1_CCS_RT_mz_DB_IUPA_charge.csv) (21/11/2019)  CompTox [UJICCSLIB List](https://comptox.epa.gov/dashboard/chemical_lists/UJICCSLIB) | UJICCSLIB [InChIKeys](https://www.norman-network.com/sites/default/files/files/suspectListExchange/230120Update/UJICCSLIB_InChIKeys_21112019.txt) (21/11/2019) | A list of 970 collision cross section values from 556 compounds (both positive and negative ionization modes, and N2 as a drift gas using TWIMS-QTOF instrument) provided by Celma et al. (2020) DOI: [10.1021/acs.est.0c05713](https://pubs.acs.org/doi/10.1021/acs.est.0c05713), [10.5281/zenodo.3549476](https://zenodo.org/record/3966751#.X7eJBy9uBQI) |
| S62 | NORMANEWS2 | **NormaNEWS2: Retrospective Screening of New Emerging Contaminants** | NormaNEWS2 as [XLSX](https://www.norman-network.com/sites/default/files/files/suspectListExchange/030220Update/NORMANEWS2_Final.xlsx), [CSV](https://www.norman-network.com/sites/default/files/files/suspectListExchange/030220Update/NORMANEWS2_Final.csv) (03/02/2020)  CompTox [NORMANEWS2 List](https://comptox.epa.gov/dashboard/chemical_lists/NORMANEWS2) | NormaNEWS2 [InChIKeys](https://www.norman-network.com/sites/default/files/files/suspectListExchange/030220Update/NORMANEWS2_InChIKeys.txt) (03/02/2020) | List of suspects provided by many contributors to [NormaNEWS2](https://www.norman-network.net/?q=node/327), collated by Kevin Thomas and colleagues at UQ.  DOI: [10.5281/zenodo.3634963](http://doi.org/10.5281/zenodo.3634963) |
| S63 | UBADWGW | **Substances Detected in Drinking (DW) or Groundwater (GW)** | UBADWGW Original File Lists 5-7 as [XLSX](https://www.norman-network.com/sites/default/files/files/suspectListExchange/030220Update/ArpHale_UBA_2019_TableA1_v2.1.xlsx) (04/02/2020)  UBADWGW as [XLSX](https://www.norman-network.com/sites/default/files/files/suspectListExchange/030220Update/S63_UBADWGW_Nov2019.xlsx), [CSV](https://www.norman-network.com/sites/default/files/files/suspectListExchange/030220Update/S63_UBADWGW_Nov2019.csv) (04/20/2020)  CompTox [UBADWGW List](https://comptox.epa.gov/dashboard/chemical_lists/UBADWGW) | UBADWGW [InChIKeys](https://www.norman-network.com/sites/default/files/files/suspectListExchange/030220Update/S63_UBADWGW_Nov2019_InChIKeys.txt) (04/20/2020) | A list of REACH registered substances (Lists 5-7) detected in drinking (DW) or groundwater (GW) that do not meet PMT criteria. Derived from a research project by Norwegian Geotechnical Institute (NGI). This couples with S36 UBAPMT (Lists 1-4) in the updated version provided by Hans Peter Arp, details in this [technical note (UBA Report 126/2019)](https://www.umweltbundesamt.de/publikationen/reach-improvement-of-guidance-methods-for-the).  DOI: [10.5281/zenodo.3637629](https://doi.org/10.5281/zenodo.3637629) |
| S64 | NATOXAQ | **NaToxAq: Natural Toxins and Drinking Water Quality - From Source to Tap** | NaToxAq as [XLSX](https://www.norman-network.com/sites/default/files/files/suspectListExchange/030320Update/S64_NaToxAq_natural_toxins_in_MassBank.xlsx), [CSV](https://www.norman-network.com/sites/default/files/files/suspectListExchange/030320Update/S64_NaToxAq_natural_toxins_in_MassBank.csv) (03/03/2020)  CompTox [NATOXAQ List](https://comptox.epa.gov/dashboard/chemical_lists/NATOXAQ) | NaToxAq [InChIKeys](https://www.norman-network.com/sites/default/files/files/suspectListExchange/030320Update/S64_NaToxAq_InChIKeys_03032020.txt) (03/03/2020) | NaToxAq ([https://natoxaq.ku.dk](https://natoxaq.ku.dk/)) is a European Training Network (ETN) funded by the European Union’s Horizon 2020 research and innovation programme under the Marie Sklodowska-Curie grant agreement No 722493, to produce knowledge about natural toxins in aquatic environments. This is a list of all NaToxAq chemicals registered in MassBank within the project, provided by Tobias Schulze, UFZ.  DOI: [10.5281/zenodo.3695174](https://doi.org/10.5281/zenodo.3695174) |
| S65 | UATHTARGETSGC | **University of Athens GC-APCI-HRMS Target List** | UATHTARGETSGC [XLSX](https://www.norman-network.com/sites/default/files/files/suspectListExchange/280520Update/UoAthens_GC-HRMS_TargetList.xlsx), [CSV](https://www.norman-network.com/sites/default/files/files/suspectListExchange/280520Update/UoAthens_GC-HRMS_TargetList.csv) (15/04/2020), CompTox [UATHTARGETSGC List](https://comptox.epa.gov/dashboard/chemical_lists/UATHTARGETSGC) | UATHTARGETSGC [InChIKeys](https://www.norman-network.com/sites/default/files/files/suspectListExchange/280520Update/S65_UoA_GCHRMSTargets_InChIKeys.txt) (15/04/2020) | GC-APCI-HRMS target list of University of Athens. Provided by the research group of Prof. Nikolaos Thomaidis (<http://trams.chem.uoa.gr/>).  Dataset DOI: [10.5281/zenodo.3753371](https://doi.org/10.5281/zenodo.3753371) |
| S66 | EAWAGTPS | **Parent-Transformation Product Pairs from Eawag** | EAWAGTPS [XLSX](https://www.norman-network.com/sites/default/files/files/suspectListExchange/280520Update/EawagTPandParents.xlsx), [CSV](https://www.norman-network.com/sites/default/files/files/suspectListExchange/280520Update/EawagTPandParents.csv) (15/05/2020)  CompTox [EAWAGTPS List](https://comptox.epa.gov/dashboard/chemical_lists/EAWAGTPS) | EAWAGTPS [InChIKeys](https://www.norman-network.com/sites/default/files/files/suspectListExchange/280520Update/EawagTPandParents_InChIKeys.txt) (15/05/2020) | Parent-Transformation Product Pairs of various micropollutants from Eawag: Swiss Federal Institute for Aquatic Science and Technology (<https://www.eawag.ch/en/>), described in Schollee et al 2017 DOI: [10.1007/s13361-017-1797-6.](http://doi.org/10.1007/s13361-017-1797-6)  Dataset DOI: [10.5281/zenodo.3754448](https://doi.org/10.5281/zenodo.3754448) |
| S67 | TBUTYLPHENOLS | **List of tert-butyl phenols from KEMI** | TBUTYLPHEOLS [XLSX](https://www.norman-network.com/sites/default/files/files/suspectListExchange/280520Update/TBUTYLPHENOLS_May2020.xlsx), [CSV](https://www.norman-network.com/sites/default/files/files/suspectListExchange/280520Update/TBUTYLPHENOLS_May2020.csv) (01/05/2020)  CompTox [TBUTYLPHENOLS List](https://comptox.epa.gov/dashboard/chemical_lists/TBUTYLPHENOLS) | TBUTYLPHENOLS [InChIKeys](https://www.norman-network.com/sites/default/files/files/suspectListExchange/280520Update/TBUTYLPHENOLS_InChIKeys.txt) (01/05/2020) | A list of tert-butyl phenols from KEMI (Swedish Chemicals Agency), partner list to BISPHENOLS. Includes exposure score.  Dataset DOI: [10.5281/zenodo.3779848](https://doi.org/10.5281/zenodo.3779848) |
| S68 | HSDBTPS | **Transformation Products Extracted from HSDB Content in PubChem** | HSDBTPS Structures [CSV](https://www.norman-network.com/sites/default/files/files/suspectListExchange/280520Update/S68_HSDBTPS_StructureInfoOnly.csv) and  Transformations [CSV](https://www.norman-network.com/sites/default/files/files/suspectListExchange/280520Update/S68_HSDBTPS_TransformationTable.csv) (16/05/2020)  CompTox [HSDBTPS List](https://comptox.epa.gov/dashboard/chemical_lists/HSDBTPS) | HSDBTPS [InChIKeys](https://www.norman-network.com/sites/default/files/files/suspectListExchange/280520Update/S68_HSDBTPS_InChIKeys.txt) (28/05/2020) | HSDBTPS is a list of metabolites / transformation products (plus parents) extracted from the "Metabolites/Metabolism" section from HSDB (Hazardous Substance Data Bank) in PubChem (<https://pubchem.ncbi.nlm.nih.gov/source/11933>). Details in Krier et al. (2022) DOI: [10.1016/j.envint.2021.106885](https://doi.org/10.1016/j.envint.2021.106885)  Dataset DOI: [10.5281/zenodo.3827487](https://doi.org/10.5281/zenodo.3827487) |
| S69 | LUXPEST | **Pesticide Screening List for Luxembourg** | LUXPEST [XLSX](https://www.norman-network.com/sites/default/files/files/suspectListExchange/100921Update/S69_LUXPEST.xlsx), [CSV](https://www.norman-network.com/sites/default/files/files/suspectListExchange/100921Update/S69_LUXPEST.csv) (29/07/2021)  CompTox [LUXPEST List](https://comptox.epa.gov/dashboard/chemical_lists/LUXPEST) | LUXPEST [InChIKeys](https://www.norman-network.com/sites/default/files/files/suspectListExchange/280520Update/S69_LUXPEST_InChIKeys.txt) (28/05/2020) | A pesticide screening list for Luxembourg, compiled from multiple sources by Jessy Krier, described in Krier et al. (2022) DOI: [10.1016/j.envint.2021.106885](https://doi.org/10.1016/j.envint.2021.106885)  Dataset DOI: [10.5281/zenodo.3862688](https://doi.org/10.5281/zenodo.3862688) |
| S70 | EISUSGCEIMS | **Environmental Institute GC-EI-MS suspect list** | EISUSGCEIMS [XLSX](https://www.norman-network.com/sites/default/files/files/suspectListExchange/150620Update/S70_EISUSGCEIMS.xlsx), [CSV](https://www.norman-network.com/sites/default/files/files/suspectListExchange/150620Update/S70_EISUSGCEIMS.csv) (15/06/2020)  CompTox [EISUSGCEIMS List](https://comptox.epa.gov/dashboard/chemical_lists/EISUSGCEIMS) | EISUSGCEIMS [InChIKeys](https://www.norman-network.com/sites/default/files/files/suspectListExchange/150620Update/S70_EISUSGCEIMS_InChIKeys.txt) (15/06/2020) | GC-EI-MS suspect list of Environmental Institute. Provided by Peter Oswald, Nikiforos Alygizakis, Martina Oswaldova, Jaroslav Slobodnik.  Dataset DOI: [10.5281/zenodo.3894827](https://doi.org/10.5281/zenodo.3894827) |
| S71 | CECSCREEN | **HBM4EU CECscreen: Screening List for Chemicals of Emerging Concern Plus Metadata and Predicted Phase 1 Metabolites** | CECscreen as [XLSX](https://www.norman-network.com/sites/default/files/files/suspectListExchange/220720Update/HBM4EU_CECscreen_DB_v3.1.xlsx), [CSV](https://www.norman-network.com/sites/default/files/files/suspectListExchange/220720Update/HBM4EU_CECscreen_DB_v3.1.csv), [README](https://www.norman-network.com/sites/default/files/files/suspectListExchange/220720Update/README_CECscreen_DataDictionary_v3.1.xlsx) (1/7/2020)  CECscreen Metabolite DB as [XLSX](https://www.norman-network.com/sites/default/files/files/suspectListExchange/220720Update/HBM4EU_CECscreen_MetabolitesDB_v1.0.xlsx), CSV (1/7/2020)  CECscreen CompTox DB as [XLSX](https://www.norman-network.com/sites/default/files/files/suspectListExchange/220720Update/HBM4EU_CECscreen_USEPACompToxDB_v2.0.xlsx), [CSV](https://www.norman-network.com/sites/default/files/files/suspectListExchange/220720Update/HBM4EU_CECscreen_USEPACompToxDB_v2.0.csv) (1/7/2020)  CECscreen OPERA Predictions as [XLSX](https://www.norman-network.com/sites/default/files/files/suspectListExchange/220720Update/HBM4EU_CECscreen_OPERAPredDB_v1.0.xlsx), [CSV](https://www.norman-network.com/sites/default/files/files/suspectListExchange/220720Update/HBM4EU_CECscreen_OPERAPredDB_v1.0.csv), [README](https://www.norman-network.com/sites/default/files/files/suspectListExchange/220720Update/README_CECscreen_OPERA2.0_models.xlsx) (1/7/2020)  CompTox [CECSCREEN List](https://comptox.epa.gov/dashboard/chemical_lists/CECSCREEN) | CECscreen [InChIKeys](https://www.norman-network.com/sites/default/files/files/suspectListExchange/220720Update/HBM4EU_CECscreen_DB_InChIKeys_v3.1.txt) (1/7/2020)  CECscreen Metabolite [InChIKeys](https://www.norman-network.com/sites/default/files/files/suspectListExchange/220720Update/HBM4EU_CECscreen_MetabolitesDB_InChIKeys_v1.0.txt) (1/7/2020) | CECScreen is part of the HBM4EU project (coord. UBA) > WP16 "emerging chemicals" (lead INRA, JP Antignac/L Debrauwer) > Task 16.1 (lead IRAS, J Vlanderen / R Vermeulen) > Main contributor (J Meijer) >Involved Partners (M Lamoree, T Hamers, S Hutinet, A, Covaci, C Huber, M Krauss, DI Walker, EL Schymanski). Meijer et al (2021) DOI: [10.1016/j.envint.2021.106511](https://linkinghub.elsevier.com/retrieve/pii/S0160412021001367).  Dataset DOI: [10.5281/zenodo.3956586](https://doi.org/10.5281/zenodo.3956586). |
| S72 | NTUPHTW | **Pharmaceutically Active Substances Suspect List from National Taiwan University** | NTUPHTW as [XLSX](https://www.norman-network.com/sites/default/files/files/suspectListExchange/220720Update/NTUPHTW_20200708.xlsx), [CSV](https://www.norman-network.com/sites/default/files/files/suspectListExchange/220720Update/NTUPHTW_20200708.csv) (22/7/2020)  CompTox [NTUPHTW List](https://comptox.epa.gov/dashboard/chemical_lists/NTUPHTW) | NTUPHTW [InChIKeys](https://www.norman-network.com/sites/default/files/files/suspectListExchange/220720Update/NTUPHTW_InChIKeys.txt) (22/7/2020) | A suspect list based on Agilent PCDLs containing pharmaceutically active substances from the College of Public Health, National Taiwan University, kindly provided by Wen-Ling Chen, details in Chen et al (2021) DOI: [10.1016/j.scitotenv.2020.141519](https://doi.org/10.1016/j.scitotenv.2020.141519)  Dataset DOI: [10.5281/zenodo.3955664](https://doi.org/10.5281/zenodo.3955664). |
| S73 | METXBIODB | **Metabolite Reaction Database from BioTransformer** | MetXBioDB substances as [CSV](https://www.norman-network.com/sites/default/files/files/suspectListExchange/061120Update/MetXBioDB_substances.csv) (06/11/2020)  MetXBioDB Transformations as [CSV](https://www.norman-network.com/sites/default/files/files/suspectListExchange/061120Update/MetXBioDB_Transformations.csv) (06/11/2020)  CompTox [METXBIODB List](https://comptox.epa.gov/dashboard/chemical_lists/METXBIODB) | MetXBioDB [InChIKeys](https://www.norman-network.com/sites/default/files/files/suspectListExchange/061120Update/MetXBioDB_InChIKeys.txt) (06/11/2020) | The Metabolite Reaction Database, MetXBioDB, is a biotransformation database used to improve the knowledge- and machine learning-based systems of BioTransformer (<http://biotransformer.ca/>) by Djoumbou-Feunang et al (2019), DOI: [10.1186/s13321-018-0324-5](https://doi.org/10.1186/s13321-018-0324-5).  Dataset DOI: [10.5281/zenodo.4056560](https://doi.org/10.5281/zenodo.4056560) |
| S74 | REFTPS | **Transformation Products and Reactions from Literature** | REFTPS substances as [CSV](https://www.norman-network.com/sites/default/files/files/suspectListExchange/010322Update/S74_REFTPS_Substances%20%282%29.csv) (25/01/2022)  REFTPS Transformations as [CSV](https://www.norman-network.com/sites/default/files/files/suspectListExchange/010322Update/S74_REFTPS_Transformations%20%282%29.csv) (25/01/2022)  CompTox REFTPS List (coming soon) | REFTPS [InChIKeys](https://www.norman-network.com/sites/default/files/files/suspectListExchange/010322Update/S74_REFTPS_InChIKeys%20%281%29.txt) (25/01/2022) | This dataset is for users to contribute transformation products and reactions in the literature to the NORMAN SLE, SusDat and the PubChem Transformations section.  Dataset DOI: [10.5281/zenodo.4318838](https://doi.org/10.5281/zenodo.4318838) |
| S75 | CYANOMETDB | **Comprehensive database of secondary metabolites from cyanobacteria** | CyanoMetDB as [XSLX](https://www.norman-network.com/sites/default/files/files/suspectListExchange/190221Update/CyanoMetDB_WR_Feb2021.xlsx), [CSV](https://www.norman-network.com/sites/default/files/files/suspectListExchange/190221Update/CyanoMetDB_WR_Feb2021.csv)  CyanoMetDB References as [XLSX](https://www.norman-network.com/sites/default/files/files/suspectListExchange/190221Update/CyanoMetDB_References_v02_WR_Feb2021.xlsx), [CSV](https://www.norman-network.com/sites/default/files/files/suspectListExchange/190221Update/CyanoMetDB_References_v02_WR_Feb2021.csv)  CyanoMetDB MetFrag [CSV](https://www.norman-network.com/sites/default/files/files/suspectListExchange/190221Update/CyanoMetDB_MetFrag_Feb2021.csv) (19/02/2021)  CompTox [CYANOMETDB](https://comptox.epa.gov/dashboard/chemical_lists/CYANOMETDB) List (19/02/2021) | CyanoMetDB [InChIKeys](https://www.norman-network.com/sites/default/files/files/suspectListExchange/190221Update/CyanoMetDB_v02_InChIKeys.txt) (19/02/2021) | CyanoMetDB is a comprehensive database of secondary metabolites from cyanobacteria manually curated from primary references described in Jones et al (2021), DOI: [10.1016/j.watres.2021.117017](https://www.sciencedirect.com/science/article/abs/pii/S0043135421002153?via%3Dihub).  Dataset DOI: [10.5281/zenodo.4551528](https://doi.org/10.5281/zenodo.4551528) |
| S76 | LUXPHARMA | **Pharmaceuticals Marketed in Luxembourg** | LUXPHARMA as [XSLX](https://www.norman-network.com/sites/default/files/files/suspectListExchange/060321Update/LUXPHARMA.xlsx), [CSV](https://www.norman-network.com/sites/default/files/files/suspectListExchange/060321Update/LUXPHARMA.csv) (06/03/2021)  CompTox [LUXPHARMA](https://comptox.epa.gov/dashboard/chemical_lists/LUXPHARMA) List | LUXPHARMA [InChIKeys](https://www.norman-network.com/sites/default/files/files/suspectListExchange/060321Update/LUXPHARMA_InChIKeys.txt) (06/03/2021) | Pharmaceuticals marketed in Luxembourg from the [CNS](https://cns.public.lu/en.html), provided by R. Singh et al. (2021) DOI: [10.1021/acsenvironau.1c00008](https://doi.org/10.1021/acsenvironau.1c00008)). List downloaded from [here](https://cns.public.lu/en/legislations/textes-coordonnes/liste-med-comm.html).  Dataset DOI: [10.5281/zenodo.4587355](https://doi.org/10.5281/zenodo.4587355). |
| S77 | FCCDB | **Food Contact Chemicals Database v5.0** | FCCdb as [XSLX](https://www.norman-network.com/sites/default/files/files/suspectListExchange/020421Update/FCCdb_201130_v5_Zenodo_wStructures%20%282%29.xlsx), [CSV](https://www.norman-network.com/sites/default/files/files/suspectListExchange/020421Update/FCCdb_FINAL_LIST_v5_wStructures%20%282%29.csv) (02/04/2021)  CompTox [FOODCONTACTSDB](https://comptox.epa.gov/dashboard/chemical_lists/FOODCONTACTSDB) List (20/02/2021) | FCCdb [InChIKeys](https://www.norman-network.com/sites/default/files/files/suspectListExchange/020421Update/FCCdb_v5_InChIKeys%20%281%29.txt) (02/04/2021) | FCCdb contains information on intentionally added food contact chemicals as described by Groh et al. (2020) DOI: [10.1016/j.envint.2020.106225](http://doi.org/10.1016/j.envint.2020.106225) and DOI: [10.5281/zenodo.4296944](http://doi.org/10.5281/zenodo.4296944), structures added by P. Chirsir (LCSB).  Dataset DOI: [10.5281/zenodo.4625495](https://doi.org/10.5281/zenodo.4625495). |
| S78 | SLUPESTTPS | **Pesticides and TPs from SLU, Sweden** | SLUPestTPs as [XLSX](https://www.norman-network.com/sites/default/files/files/suspectListExchange/140421Update/SLUPestTPs_MengerBostr%C3%B6m.xlsx), [CSV](https://www.norman-network.com/sites/default/files/files/suspectListExchange/140421Update/SLUPestTPs_suspects.csv) (14/04/2021)  SLUPestTPs Transformations as [CSV](https://www.norman-network.com/sites/default/files/files/suspectListExchange/140421Update/SLUPestTPs_transformations.csv) (14/04/2021)  CompTox [SLUPESTTPS](https://comptox.epa.gov/dashboard/chemical_lists/SLUPESTTPS) List | SLUPestTPs [InChIKeys](https://www.norman-network.com/sites/default/files/files/suspectListExchange/140421Update/SLUPESTTPS_InChIKeys.txt) (14/04/2021) | Suspect list of pesticides and pesticide transformation products (TPs) from SLU, created based on Sweden’s national monitoring program and the pesticide properties database (PPDB) described in Frank Menger et al (2021) DOI: [10.1021/acs.est.1c00466](https://pubs.acs.org/doi/10.1021/acs.est.1c00466).  Dataset DOI: [10.5281/zenodo.4687924](https://doi.org/10.5281/zenodo.4687924). |
| S79 | UACCSCEC | **Collision Cross Section (CCS) Library from UAntwerp** | UACCSCEC as [XLSX](https://www.norman-network.com/sites/default/files/files/suspectListExchange/200421Update/UACCSCEC_ac1c00142_si_002_TS-5.xlsx), [CSV](https://www.norman-network.com/sites/default/files/files/suspectListExchange/200421Update/UACCSCEC_ac1c00142_si_002_TS-5.csv) (20/04/2021)  CompTox [UACCSCEC](https://comptox.epa.gov/dashboard/chemical_lists/UACCSCEC) List | UACCSCEC [InChIKeys](https://www.norman-network.com/sites/default/files/files/suspectListExchange/200421Update/UACCSCEC_InChIKeys.txt) (20/04/2021) | Collision cross section (CCS) values of 311 adducts of 148 contaminants of emerging concern (CECs) and their metabolites measured with drift tube ion mobility high resolution mass spectrometry (positive and negative ionization, N2 drift gas) as described in Belova et al (2021) DOI: [10.1021/acs.analchem.1c00142](https://doi.org/10.1021/acs.analchem.1c00142).  Dataset DOI: [10.5281/zenodo.4704648](https://doi.org/10.5281/zenodo.4704648). |
| S80 | PFASGLUEGE | **Overview of PFAS Uses** | Original Files as XLSM [ESI-2](https://www.norman-network.com/sites/default/files/files/suspectListExchange/062521Update/ESI-2.xlsm), [ESI-3](https://www.norman-network.com/sites/default/files/files/suspectListExchange/062521Update/ESI-3.xlsm)  PFAS Substances as [XLSX](https://www.norman-network.com/sites/default/files/files/suspectListExchange/062521Update/PFASGLUEGE_Substances.xlsx), [CSV](https://www.norman-network.com/sites/default/files/files/suspectListExchange/062521Update/PFASGLUEGE_Substances.csv)  PFAS Use Information (single sheet) as [XLSX](https://www.norman-network.com/sites/default/files/files/suspectListExchange/062521Update/ESI3_all_uses_wStruct.xlsx), [CSV](https://www.norman-network.com/sites/default/files/files/suspectListExchange/062521Update/ESI3_all_uses_wStruct.csv) (24/06/2021)  CompTox [PFASGLUEGE](https://comptox.epa.gov/dashboard/chemical_lists/PFASGLUEGE) List | PFASGLUEGE [InChIKeys](https://www.norman-network.com/sites/default/files/files/suspectListExchange/062521Update/PFASGLUEGE_InChIKeys.txt) (24/06/2021) | An overview of the uses of per- and polyfluoroalkyl substances (PFAS) from Glüge et al (2020) DOI: [10.1039/D0EM00291G](https://doi.org/10.1039/D0EM00291G).  Dataset DOI: [10.5281/zenodo.5029173](https://doi.org/10.5281/zenodo.5029173). |
| S81 | THSTPS | **Thirdhand Smoke Specific Metabolites** | THSTPS in [XLSX](https://www.norman-network.com/sites/default/files/files/suspectListExchange/100921Update/S81_THSTPS_20210831.xlsx), [CSV](https://www.norman-network.com/sites/default/files/files/suspectListExchange/100921Update/S81_THSTPS_20210831.csv) (02/09/2021)  THSTPS Transformations in [CSV](https://www.norman-network.com/sites/default/files/files/suspectListExchange/100921Update/S81_THSTPS_Transformations.csv) (02/09/2021)  CompTox [THSTPS](https://comptox.epa.gov/dashboard/chemical-lists/THSTPS) List | THSTPS [InChIKeys](https://www.norman-network.com/sites/default/files/files/suspectListExchange/100921Update/S81_THSTPS_InChIKeys.txt) (02/09/2021) | A list of Thirdhand Smoke (THS) specific metabolites compiled by Carla Merino, Noelia Ramirez and colleagues (IISPV-URV).  Dataset DOI: [10.5281/zenodo.5394629](https://doi.org/10.5281/zenodo.5394629) |
| S82 | EAWAGPMT | **PMT Suspect List from Eawag** | EAWAGPMT in [XLSX](https://www.norman-network.com/sites/default/files/files/suspectListExchange/100921Update/EAWAGPMT_SuspectList_Kiefer_et_al2021.xlsx), [CSV](https://www.norman-network.com/sites/default/files/files/suspectListExchange/100921Update/EAWAGPMT_SuspectList_Kiefer_et_al2021.csv) (10/09/2021)  CompTox [EAWAGPMT](https://comptox.epa.gov/dashboard/chemical-lists/EAWAGPMT) List | EAWAGPMT [InChIKeys](https://www.norman-network.com/sites/default/files/files/suspectListExchange/100921Update/EAWAGPMT_InChIKeys.txt) (10/09/2021) | A PMT suspect screening list containing >1100 entries from Kiefer et al (2021), DOI: [10.1016/j.watres.2021.116994](https://doi.org/10.1016/j.watres.2021.116994).  Dataset DOI: [10.5281/zenodo.5500131](https://doi.org/10.5281/zenodo.5500131) |
| S83 | CCL5 | **Contaminant Candidate List CCL 5 (Draft)** | CCL5 in [XLSX](https://www.norman-network.com/sites/default/files/files/suspectListExchange/241121Update/CCL5.xlsx), [CSV](https://www.norman-network.com/sites/default/files/files/suspectListExchange/241121Update/CCL5.csv) (23/11/2021)  CompTox CCL5 List (coming soon…) | CCL5 [InChIKeys](https://www.norman-network.com/sites/default/files/files/suspectListExchange/241121Update/CCL5_InChIKeys.txt) (23/11/2021) | The CCL 5 is a (currently draft) list of contaminants that are currently not subject to any proposed or promulgated US primary drinking water regulations, but are known or anticipated to occur in public water systems. Microcystins are listed in S85. From: <https://www.epa.gov/ccl/contaminant-candidate-list-5-ccl-5>.  Dataset DOI: [10.5281/zenodo.5533801](https://doi.org/10.5281/zenodo.5533801) |
| S84 | UFZHSFPMT | **PMT Suspect List from UFZ and HSF** | UFZHSFPMT in [XLSX](https://www.norman-network.com/sites/default/files/files/suspectListExchange/290921Update/UFZ_HSF_SuspectList_PM_candidates_210909.xlsx), [CSV](https://www.norman-network.com/sites/default/files/files/suspectListExchange/290921Update/UFZ_HSF_SuspectList_PM_candidates_210909.csv) (29/09/2021)  CompTox [UFZHSFPMT](https://comptox.epa.gov/dashboard/chemical-lists/UFZHSFPMT) List | UFZHSFPMT [InChIKeys](https://www.norman-network.com/sites/default/files/files/suspectListExchange/290921Update/UFZHSFPMT_InChIKeys.txt) (29/09/2021) | A suspect screening list of 1310 potentially persistent and mobile chemicals from UFZ and HSF, described in Neuwald, Muschket et al. (2021) DOI: [10.1016/j.watres.2021.117645](https://doi.org/10.1016/j.watres.2021.117645) & [10.5281/zenodo.5503379](https://doi.org/10.5281/zenodo.5503379)  Dataset DOI: [10.5281/zenodo.5535287](https://doi.org/10.5281/zenodo.5535287) |
| S85 | MICROCYSTINS | **MICROCYSTINS from CyanoMetDB** | MICROCYSTINS in [XLSX](https://www.norman-network.com/sites/default/files/files/suspectListExchange/241121Update/MICROCYSTINS.xlsx), [CSV](https://www.norman-network.com/sites/default/files/files/suspectListExchange/241121Update/MICROCYSTINS.csv) (10/11/2021)  CompTox MICROCYSTINS List (coming soon…) | MICROCYSTINS [InChIKeys](https://www.norman-network.com/sites/default/files/files/suspectListExchange/241121Update/MICROCYSTINS_InChIKeys.txt) (10/11/2021) | A list of over 300 microcystins extracted from CyanoMetDB (latest version), mostly of natural origin. Provided by L. Janssen, Eawag.  Dataset DOI: [10.5281/zenodo.5665355](https://doi.org/10.5281/zenodo.5665355) |
| S86 | TATTOOINK | **TATTOOINK as per EU regulation 2020/2081** | TATTOOINK in [XLSX](https://www.norman-network.com/sites/default/files/files/suspectListExchange/241121Update/TATTOOINK.xlsx), [CSV](https://www.norman-network.com/sites/default/files/files/suspectListExchange/241121Update/TATTOOINK.csv) (18/11/2021)  CompTox [TATTOOINK](https://comptox.epa.gov/dashboard/chemical-lists/TATTOOINK) List | TATTOOINK [InChIKeys](https://www.norman-network.com/sites/default/files/files/suspectListExchange/241121Update/TATTOOINK_InChIKeys.txt) (18/11/2021) | A list of regulated ingredients for tattoo ink and permanent make up, Appendix 13 added to Commission Regulation (EU) [2020/2081](https://www.norman-network.com/sites/default/files/files/suspectListExchange/241121Update/Tattoo%20inks_%28EU%292020_2081.pdf), 14 December 2020 amending [Annex XVII of REACH](https://eur-lex.europa.eu/eli/reg/2008/1272/oj).  Dataset DOI: [10.5281/zenodo.5710243](https://doi.org/10.5281/zenodo.5710243) |
| S87 | CHLORINETPS | **List of chlorination byproducts of 137 CECs and small disinfection byproducts** | CHLORINETPS in [XLSX](https://www.norman-network.com/sites/default/files/files/suspectListExchange/091221Update/CHLORINETPS.xlsx), [CSV](https://www.norman-network.com/sites/default/files/files/suspectListExchange/091221Update/CHLORINETPS.csv) (09/12/2021)  CompTox [CHLORINETPS](https://comptox.epa.gov/dashboard/chemical-lists/CHLORINETPS) List | CHLORINETPS [InChIKeys](https://www.norman-network.com/sites/default/files/files/suspectListExchange/091221Update/CHLORINETPS_InChIKeys.txt) (09/12/2021) | A list of chlorination byproducts of 137 contaminants of emerging concern (CECs) and small molecular weight disinfection byproducts from the CHLORINE_TPs database, described in Postigo et al DOI: [10.1016/j.teac.2021.e00148](https://doi.org/10.1016/j.teac.2021.e00148). 91% are amenable to LC-ESI-HRMS.  Dataset DOI: [10.5281/zenodo.5767356](https://doi.org/10.5281/zenodo.5767356) |
| S88 | UBABIOCIDES | **List of Prioritized Biocides from UBA** | UBABIOCIDES in [XLSX](https://www.norman-network.com/sites/default/files/files/suspectListExchange/091221Update/UBABIOCIDES.xlsx), [CSV](https://www.norman-network.com/sites/default/files/files/suspectListExchange/091221Update/UBABIOCIDES.csv) (09/12/2021)  CompTox [UBABIOCIDES](https://comptox.epa.gov/dashboard/chemical-lists/UBABIOCIDES) List | UBABIOCIDES [InChIKeys](https://www.norman-network.com/sites/default/files/files/suspectListExchange/091221Update/UBABIOCIDES_InChIKeys.txt) (09/12/2021) | A merged list of prioritized biocidal active substances provided by UBA (German Environment Agency) and relevant transformation products, released Sept. 2021 ([PDF](https://www.umweltbundesamt.de/sites/default/files/medien/1410/publikationen/addendum_priolisten_final_0.pdf)) and updating [UBA Texte 114/2017](https://www.umweltbundesamt.de/en/publikationen/are-biocide-emissions-into-the-environment-already).  Dataset DOI: [10.5281/zenodo.5767494](https://doi.org/10.5281/zenodo.5767494) |
| S89 | PRORISKPFAS | **List of PFAS Compiled from NORMAN SusDat** | PRORISKPFAS in [XLSX](https://www.norman-network.com/sites/default/files/files/suspectListExchange/091221Update/PRORISKPFAS.xlsx), [CSV](https://www.norman-network.com/sites/default/files/files/suspectListExchange/091221Update/PRORISKPFAS.csv) (09/12/2021)  CompTox [PRORISKPFAS](https://comptox.epa.gov/dashboard/chemical-lists/PRORISKPFAS) List | PRORISKPFAS [InChIKeys](https://www.norman-network.com/sites/default/files/files/suspectListExchange/091221Update/PRORISKPFAS_InChIKeys.txt) (09/12/2021) | A compiled list of 4777 PFAS occurring in NORMAN SusDat, created by merging SLE lists S9, S14, S25, S46, and S80 and searching for additional fluorinated content in SusDat. Provided by Kelsey Ng, EI (manuscript in prep.).  Dataset DOI: [10.5281/zenodo.5769582](https://doi.org/10.5281/zenodo.5769582) |
| S90 | ZEROPMBOX1 | **ZeroPM Box 1 Substances** | ZeroPM Box 1 as [XLSX](https://www.norman-network.com/sites/default/files/files/suspectListExchange/180122Upate/ZeroPM_Box1.xlsx), [CSV](https://www.norman-network.com/sites/default/files/files/suspectListExchange/180122Upate/ZeroPM_Box1.csv) (15/01/2022)  CompTox [ZEROPMBOX1](https://comptox.epa.gov/dashboard/chemical-lists/ZEROPMBOX1) List | ZeroPM Box1 [InChIKeys](https://www.norman-network.com/sites/default/files/files/suspectListExchange/180122Upate/ZeroPM_Box1_InChIKeys.txt) (15/01/2022) | This list contains representative PFAS, triazoles and triazines, the "Box 1" substances to kick-off the H2020 project ZeroPM ([https://zeropm.eu](https://zeropm.eu/)).  Dataset DOI: [10.5281/zenodo.5854251](https://doi.org/10.5281/zenodo.5854251) |
| S91 | CECTOYS | **Chemicals of Emerging Concern (CECs) in plastic toys** | CECTOYS in [XLSX](https://www.norman-network.com/sites/default/files/files/suspectListExchange/290322Update/CECTOYS.xlsx), [CSV](https://www.norman-network.com/sites/default/files/files/suspectListExchange/290322Update/CECTOYS.csv) (31/01/2022) | CECTOYS [InChIKeys](https://www.norman-network.com/sites/default/files/files/suspectListExchange/290322Update/CECTOYS_InChIKey.txt) (31/01/2022) | A list of 126 Chemicals of EmergingConcern (CECs) found in plastic toys described in Aurisano et al. The list is categorized into four based on being included in regulatory lists of concern as well as reported hazard index (HI) and child cancer risk (CCR) based criteria. DOI: [10.1016/j.envint.2020.106194](https://doi.org/10.1016/j.envint.2020.106194).  Dataset DOI: [10.5281/zenodo.5933614](https://doi.org/10.5281/zenodo.5933614) |
| S92 | FLUOROPHARMA | **List of 340 ATC classified fluoro-pharmaceuticals** | FLUOROPHARMA in [XLSX](https://www.norman-network.com/sites/default/files/files/suspectListExchange/010322Update/FLUOROPHARMA.xlsx), [CSV](https://www.norman-network.com/sites/default/files/files/suspectListExchange/010322Update/FLUOROPHARMA.csv) (05/02/2022)  CompTox List FLUOROPHARMA (coming soon…) | FLUOROPHARMA [InChIKeys](https://www.norman-network.com/sites/default/files/files/suspectListExchange/010322Update/FLUOROPHARMA_InChIKey.txt) (05/02/2022) | A list of 340 fluoro pharmaceuticals classified as per WHO's Anatomical Therapeutic Chemical (ATC) classification based on their medical application, described in Inoue et al DOI: [10.1021/acsomega.0c00830](https://pubs.acs.org/doi/10.1021/acsomega.0c00830).  Dataset DOI: [10.5281/zenodo.5979647](https://doi.org/10.5281/zenodo.5979647) |
| S93 | CECMOUTHING | **Chemicals of Emerging Concern (CECs) in children's mouthing exposure** | CECMOUTHING Substances in [XLSX](https://www.norman-network.com/sites/default/files/files/suspectListExchange/290322Update/CECMOUTHING_Substances.xlsx), [CSV](https://www.norman-network.com/sites/default/files/files/suspectListExchange/290322Update/CECMOUTHING_Substances.csv) (05/02/2022)  CECMOUTHING Values in [XLSX](https://www.norman-network.com/sites/default/files/files/suspectListExchange/290322Update/CECMOUTHING_values.xlsx), [CSV](https://www.norman-network.com/sites/default/files/files/suspectListExchange/290322Update/CECMOUTHING_values.csv) (05/02/2022) | CECMOUTHING [InChIKeys](https://www.norman-network.com/sites/default/files/files/suspectListExchange/290322Update/CECMOUTHING_InChIKey.txt) (05/02/2022) | A dataset of 60 Chemicals of Emerging Concern (CECs) and their chemical migration rate into saliva due to mouthing exposure in children studied in Aurisano et. al DOI: [10.1038/s41370-021-00354-0](https://www.nature.com/articles/s41370-021-00354-0).  Dataset DOI: [10.5281/zenodo.5979673](https://doi.org/10.5281/zenodo.5979673) |
| S94 | FLUOROPEST | **List of 423 FRAC/HRAC/IRAC classified fluoro-agrochemicals** | FLUOROPEST in [XLSX](https://www.norman-network.com/sites/default/files/files/suspectListExchange/010322Update/FLUOROPEST.xlsx), [CSV](https://www.norman-network.com/sites/default/files/files/suspectListExchange/010322Update/FLUOROPEST.csv) (21/02/2022)  CompTox List FLUOROPEST (coming soon…) | FLUOROPEST [InChIKeys](https://www.norman-network.com/sites/default/files/files/suspectListExchange/010322Update/FLUOROPEST_InChIKey.txt) (21/02/2022) | A list of 423 Fungicide Resistance Action Committee (FRAC), Herbicide Resistance Action Committee (HRAC) or Insecticide Resistance Action Committee (IRAC) classified fluoro-agrochemicals based on their chemotype and mode of action, described in Ogawa et al DOI: [10.1016/j.isci.2020.101467](https://www.cell.com/iscience/fulltext/S2589-0042(20)30659-3?_returnURL=https%3A%2F%2Flinkinghub.elsevier.com%2Fretrieve%2Fpii%2FS2589004220306593%3Fshowall%3Dtrue).  Dataset DOI: [10.5281/zenodo.6201559](https://doi.org/10.5281/zenodo.6201559) |
| S95 | PFASANEXCH | **PFAS List from the NORMAN PFAS Analytical Exchange Activity** | PFASANEXCH in [XLSX](https://www.norman-network.com/sites/default/files/files/suspectListExchange/290322Update/PFASANEXCH.xlsx), [CSV](https://www.norman-network.com/sites/default/files/files/suspectListExchange/290322Update/PFASANEXCH.csv) (25/03/2022) | PFASANEXCH [InChIKeys](https://www.norman-network.com/sites/default/files/files/suspectListExchange/290322Update/PFASANEXCH_InChIKey.txt) (25/03/2022) | A list from the PFAS Analytical Exchange Activity, part of NORMAN Joint Programme of Activities (JPA) 2021 coordinated by UK Environment Agency.  This activity aimed to gain an understanding of the current analytical capability of PFAS as Limit of Detection (LOD) in participating international laboratories.  Dataset DOI: [10.5281/zenodo.6384309](https://doi.org/10.5281/zenodo.6384309) |
| S96 | ECIPFAS | **Updatable List to add PFAS Structures to Public Resources from ECI (UniLu)** | ECIPFAS in [XLSX](https://www.norman-network.com/sites/default/files/files/suspectListExchange/290322Update/S96_ECIPFAS_Substances.xlsx), [CSV](https://www.norman-network.com/sites/default/files/files/suspectListExchange/290322Update/S96_ECIPFAS_Substances.csv) (28/03/2022) | ECIPFAS [InChIKeys](https://www.norman-network.com/sites/default/files/files/suspectListExchange/290322Update/S96_ECIPFAS_InChIKeys.txt) (28/03/2022) | This dataset is for users to contribute PFAS from the literature or other documents to the NORMAN SLE, SusDat and PubChem (to fill database gaps).  Dataset DOI: [10.5281/zenodo.6389740](https://doi.org/10.5281/zenodo.6389740) |
| S97 | UBABPAALT | **List of Bisphenol A Alternatives from UBA** | UBABPAALT as [XLSX](https://www.norman-network.com/sites/default/files/files/suspectListExchange/290422Update/UBABPAALT.xlsx), [CSV](https://www.norman-network.com/sites/default/files/files/suspectListExchange/290422Update/UBABPAALT.csv) (01/04/2022) | UBABPAALT [InChIKeys](https://www.norman-network.com/sites/default/files/files/suspectListExchange/290422Update/UBABPAALT_InChIKeys.txt) (01/04/2022) | A list of Bisphenol A alternatives provided by UBA (German Environment Agency) from [UBA Report 123/2019](https://www.umweltbundesamt.de/en/publikationen/bewertung-des-endokrinen-potenzials-von-bisphenol).  Dataset DOI: [10.5281/zenodo.6405325](https://doi.org/10.5281/zenodo.6405325) |
| S98 | TIRECHEM | **Tire-Related Chemicals in Environment from Literature** | TIRECHEM as [XLSX](https://www.norman-network.com/sites/default/files/files/suspectListExchange/290422Update/TIRECHEM.xlsx), [CSV](https://www.norman-network.com/sites/default/files/files/suspectListExchange/290422Update/TIRECHEM.csv) (01/04/2022) | TIRECHEM [InChIKeys](https://www.norman-network.com/sites/default/files/files/suspectListExchange/290422Update/TIRECHEM_InChIKeys.txt) (01/04/2022) | This list includes tire-related chemicals found in the environment plus analytical data from literature studies and will be expanded as new data is published.  Dataset DOI: [10.5281/zenodo.6405358](https://doi.org/10.5281/zenodo.6405358) |

## About NORMAN-SLE (header at <https://www.norman-network.com/nds/SLE/>)

The NORMAN Suspect List Exchange (NORMAN-SLE) was established in 2015 as a central access point for NORMAN members (and others) to find suspect lists relevant for their environmental monitoring questions. The NORMAN-SLE documents all individual collections that form a part of the merged collection [NORMAN SusDat](http://www.norman-network.com/nds/susdat/). The original SLE lists should be consulted to verify SusDat information if necessary (see Source column in SusDat). NORMAN-SLE versions are tracked on [Zenodo](https://zenodo.org/communities/norman-sle).

Comments and contributions are welcome - please email us at [suspects@normandata.eu](mailto:suspects@normandata.eu).

Please refer to our [documentation](https://gitlab.lcsb.uni.lu/eci/NORMAN-SLE/-/tree/master/docs) pages for: [citation](https://gitlab.lcsb.uni.lu/eci/NORMAN-SLE/-/blob/master/docs/CitingSLE.md) instructions, [credits](https://gitlab.lcsb.uni.lu/eci/NORMAN-SLE/-/blob/master/docs/CreditsSLE.md), [updates](https://gitlab.lcsb.uni.lu/eci/NORMAN-SLE/-/blob/master/docs/UpdatesSLE.md), [license](https://gitlab.lcsb.uni.lu/eci/NORMAN-SLE/-/blob/master/docs/LicenseSLE.md) details, [SDFs](https://gitlab.lcsb.uni.lu/eci/NORMAN-SLE/-/blob/master/docs/SLEtoSDF.md) and other useful tips!
